# Supplementary figures and images for: Beyond the main function: An experimental study of the use of hardwood boomerangs in retouching activities
Source: PLoS One. 2022 Aug 16;17(8):e0273118. doi: 10.1371/journal.pone.0273118 (PMC9380927; doi:10.1371/journal.pone.0273118)

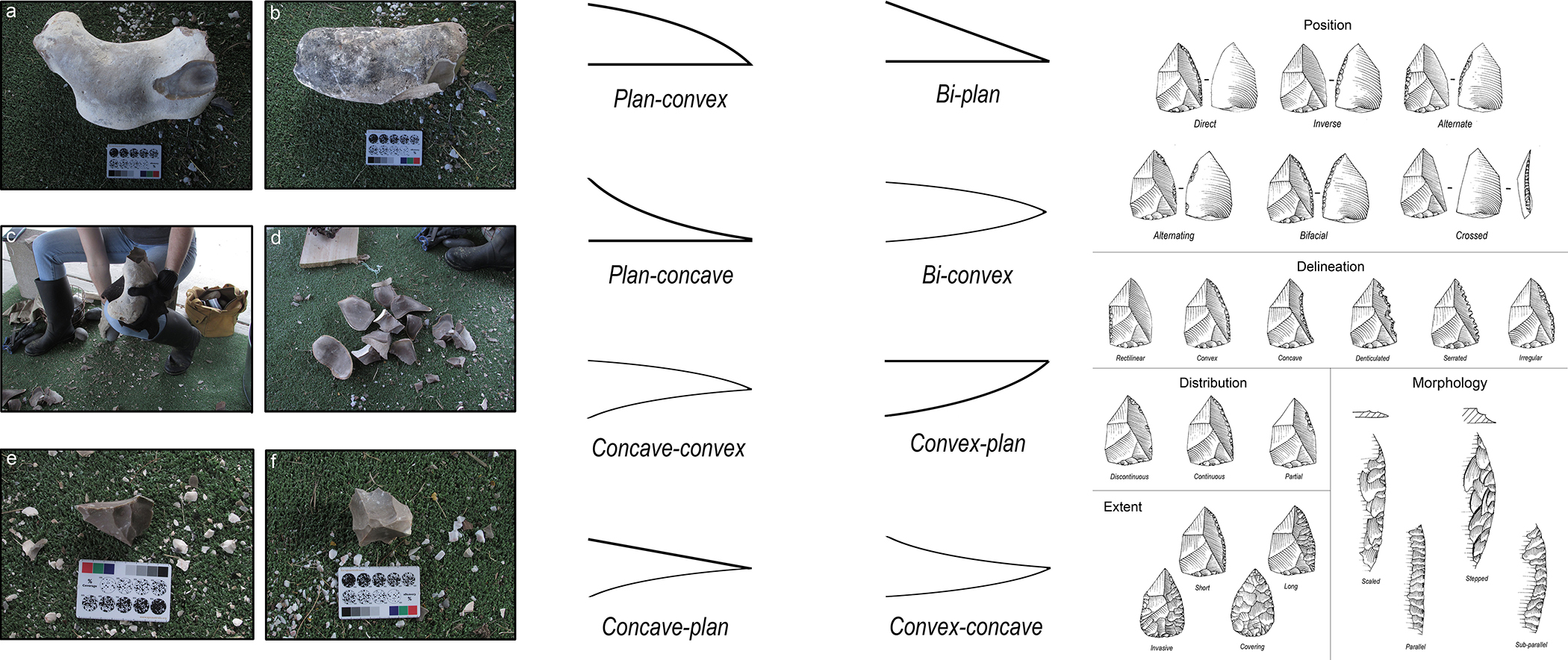

Supplement: S1 Fig — A. Knapping of experimental lithic flakes. (a) cobble_1 before knapping; (b) cobble_2 before knapping; (c) bifacial edge reduction; (d) sample of produced flake blanks; (e) exhausted core after knapping of cobble_1; (f) exhausted core after knapping of cobble_2. B. Types of cross-sections of functional edges of lithic tools. From [28]. C. Description of retouched edges. From [39]. (JPG) [file pone.0273118.s001.jpg]

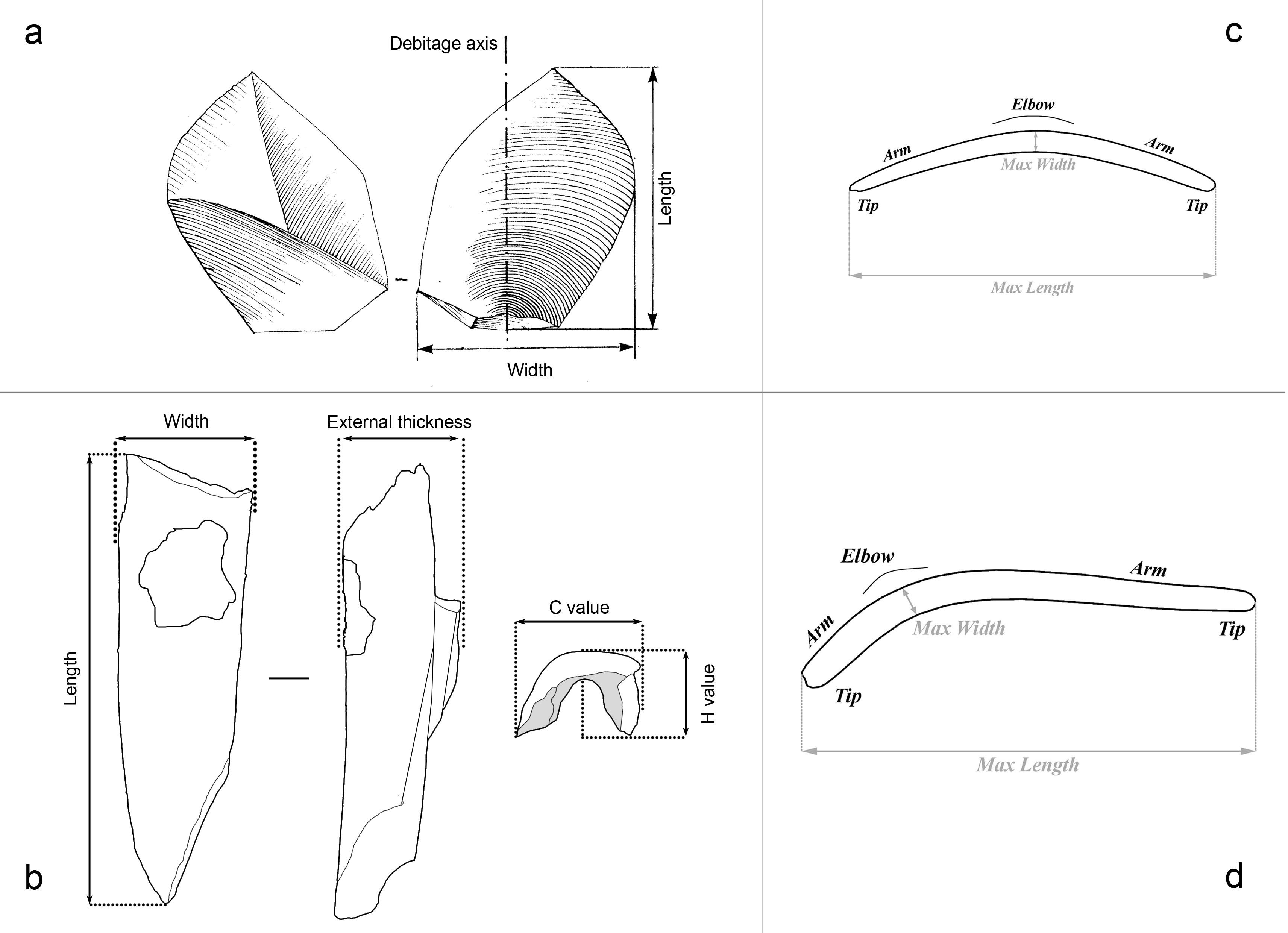

Supplement: S2 Fig — (a) measurements of lithic flakes according to their debitage axis; (b) measurement of bone retouchers; recording of ‘C’ and ‘H’ values follows Neruda and Lázničková-Galetová (2018); (c) measurement of symmetrical boomerangs; (d) measurement of asymmetrical boomerangs. Drawings by E. F. Martellotta. (JPG) [file pone.0273118.s002.jpg]

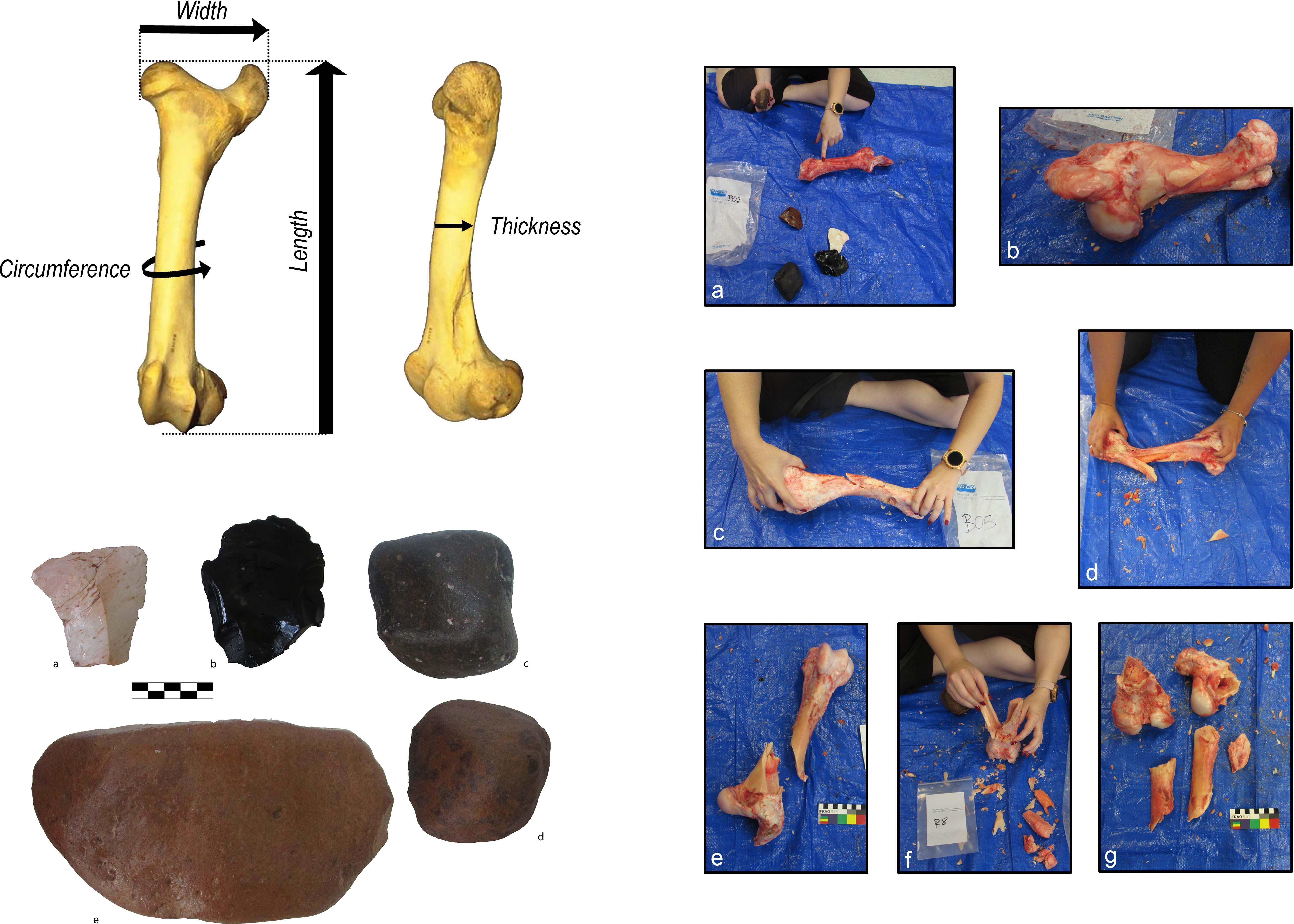

Supplement: S3 Fig — A. Measurement method applied to bones before breaking. Drawing by E. F. Martellotta. B. Tools used during the bone breaking session. (a, b) flint flakes occasionally used to remove periosteum; (c) hammerstone 2, i.e., H2; (d) hammerstone 1, i.e., H1; (e) anvil. C. Bone breaking session. (a) choice of the impact point; (b) incipient fracture; (c-d) the bone is completely fractured through flexion applied to the incipient fracture; (e) spiral fracture; (f) detachment of bone retouchers from fractured diaphysis; (g) obtained bone retouchers and discarded epiphyses. (JPG) [file pone.0273118.s003.jpg]

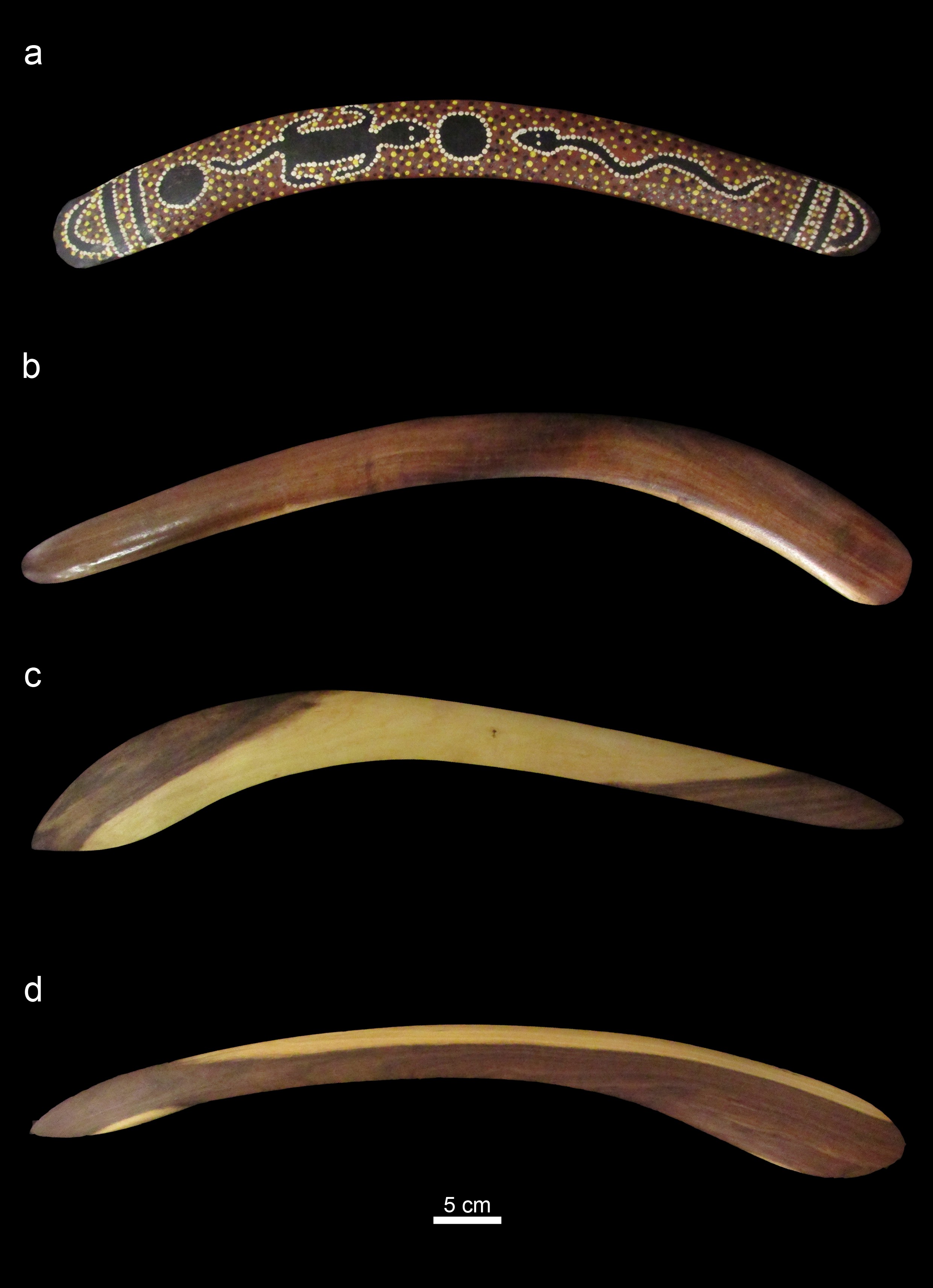

Supplement: S4 Fig — (a) B1; (b) B2; (c) B3; (d) B4. (JPG) [file pone.0273118.s004.jpg]

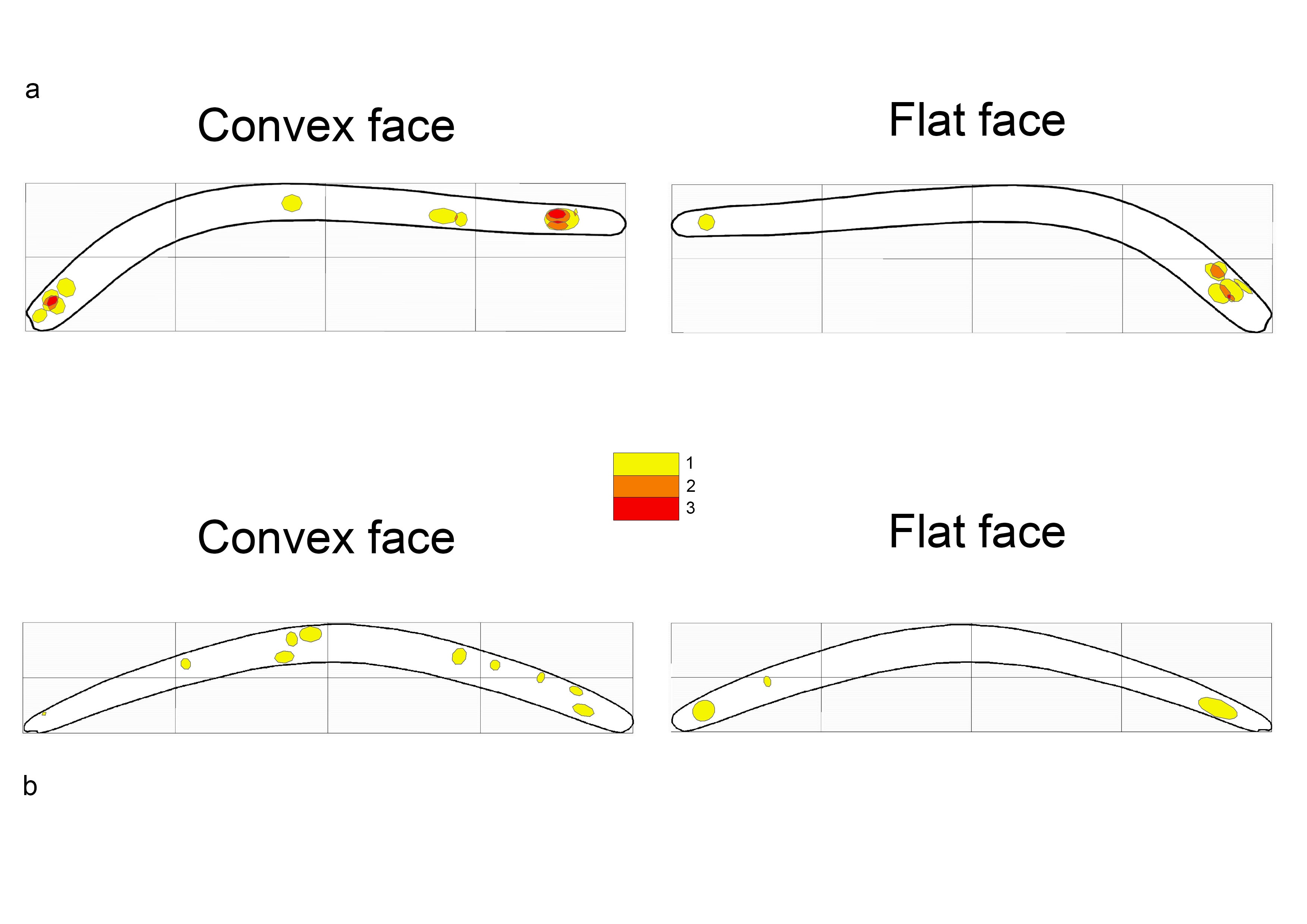

Supplement: S5 Fig — (a) asymmetrical shape; (b) symmetrical shape. From: Martellotta EF, Wilkins J, Brumm A, Langley MC. New data from old collections: Retouch-induced marks on Australian hardwood boomerangs. J Archaeol Sci Reports. 2021;37: 102967. doi:10.1016/j.jasrep.2021.102967. (JPG) [file pone.0273118.s005.jpg]
