# Supplementary material for: Beyond the main function: An experimental study of the use of hardwood boomerangs in retouching activities
Source: PLoS One. 2022 Aug 16;17(8):e0273118. doi: 10.1371/journal.pone.0273118 (PMC9380927; doi:10.1371/journal.pone.0273118)
Supplement: S6 Fig — A. Metric data on use areas identified on boomerangs (top) and bone retouchers (bottom). Statistical significance of data—Length of use areas: R = 0.21; p = 0.43. Width of use areas: R = -0.24; p = 0.36. Surface of use areas: R = 0.12, p = 0.64. Perimeter of use areas: R = 0.4; p = 0.88. B. Representation of retouch intensity on boomerangs and retouchers. (a) retouch intensity of use areas on boomerangs; (b) retouch intensity of use areas on bone retouchers. (c) different intensity of retouch produced by the two knappers during retouching session 1; note that T.R.M. produced a greater variability of impact traces distribution than Y.L.P. C. Distribution of the two main categories of impact traces (linear and punctiform impressions) on boomerangs and retouchers. Numbers on the x axis indicate the frequency of observed impact traces in a single use area. ‘NA’ stands for ‘use areas not showing the impressions category’. D. Scatter plot of liner and punctiform impressions on boomerangs and retouchers. Correlation coefficient with Pearson’s method and confidence of intervals (95%) applied. E. Scatter plot showing negative correlation between notches and linear impressions on boomerangs. Correlation coefficient with Pearson’s method and confidence of intervals (95%) applied. F. Linear and punctiform impressions metric data: Comparison between boomerangs and bone retouchers. Note how the difference in frequencies makes a proper comparison difficult. G. Metric data for linear and punctiform impressions: Comparison between a sample of measurements form boomerangs and bone retouchers (N = 256). The sample of 256 measurements was randomly generated in the R software using the ‘sample()’ function. H. Scatter plot showing correlations among the length measurements for linear impressions on boomerangs and bone retouchers. Correlation coefficient with Pearson’s method and confidence of intervals (95%) applied. I. Scatter plot showing correlations among the surface measurements for [file pone.0273118.s006.pdf]

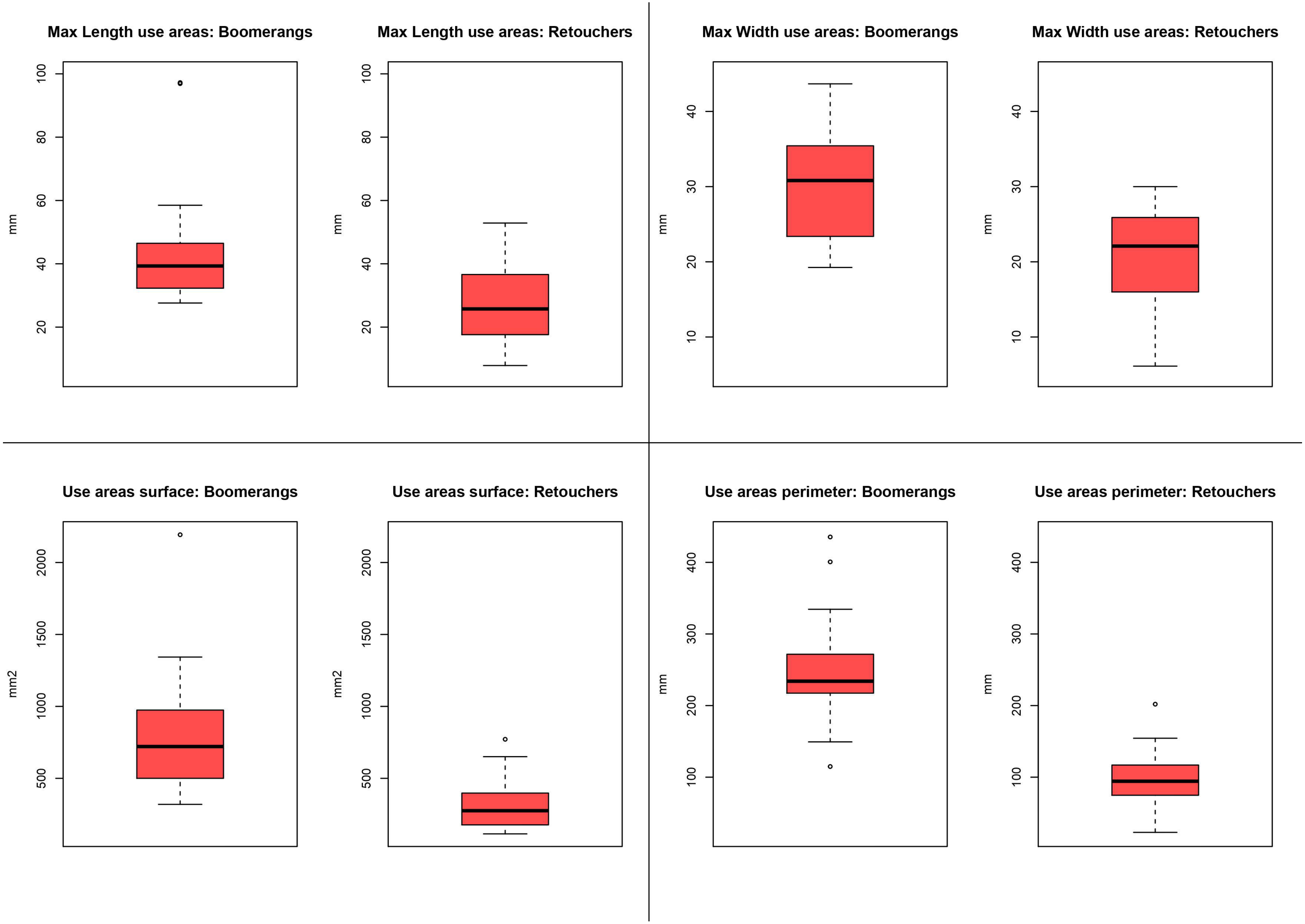

Max Length use areas: Boomerangs
Max Length use areas: Retouchers
Max Width use areas: Boomerangs
Max Width use areas: Retouchers
Use areas surface: Boomerangs
Use areas surface: Retouchers
Use areas perimeter: Boomerangs
Use areas perimeter: Retouchers
mm
mm2
20 40 60 80 100
10 20 30 40
500 1000 1500 2000
100 200 300 400

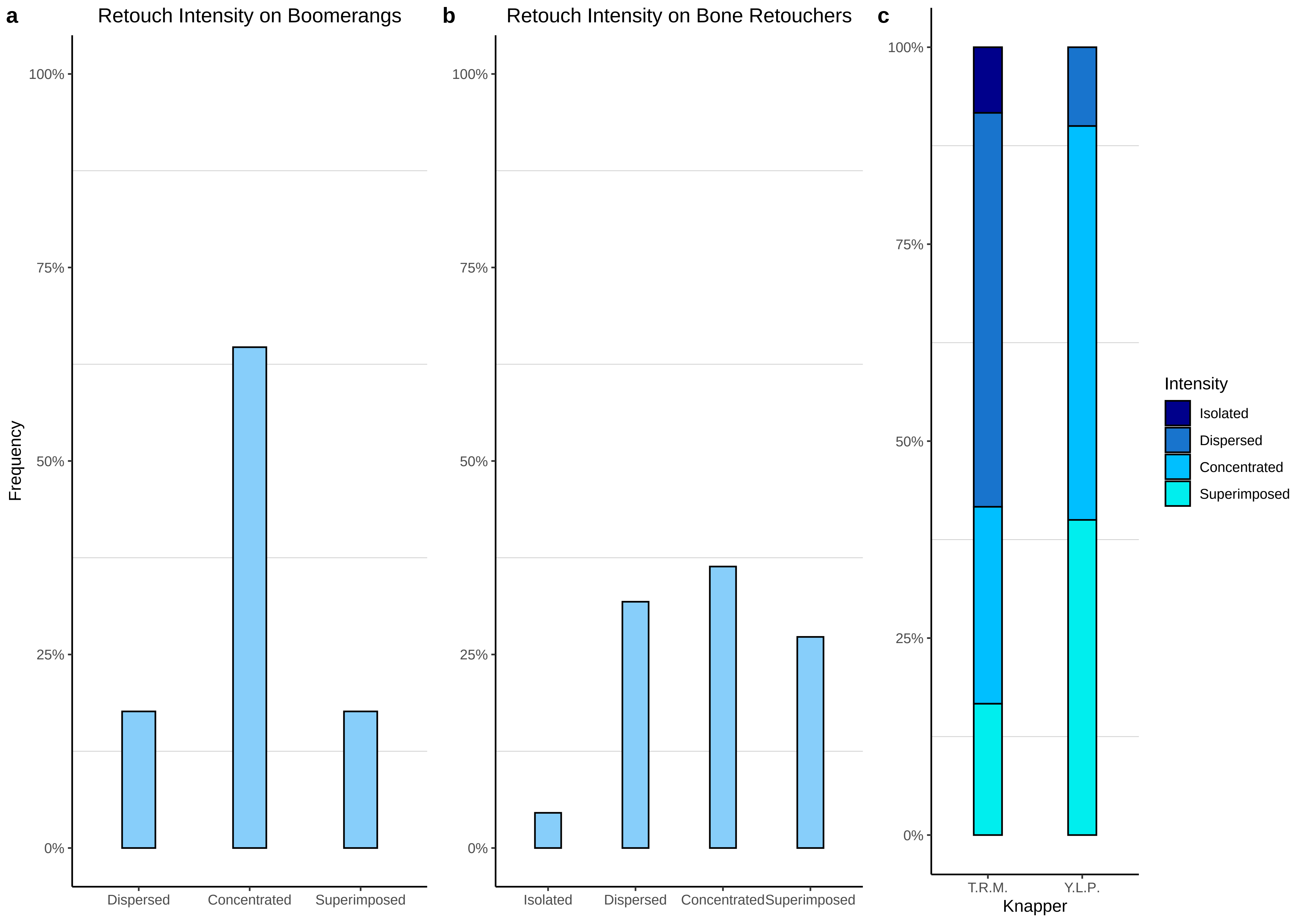
a
Retouch Intensity on Boomerangs
b
Retouch Intensity on Bone Retouchers
c
Frequency
100%
75%
50%
25%
0%
Dispersed
Concentrated
Superimposed
Isolated
T.R.M.
Y.L.P.
Knapper
Intensity
Isolated
Dispersed
Concentrated
Superimposed

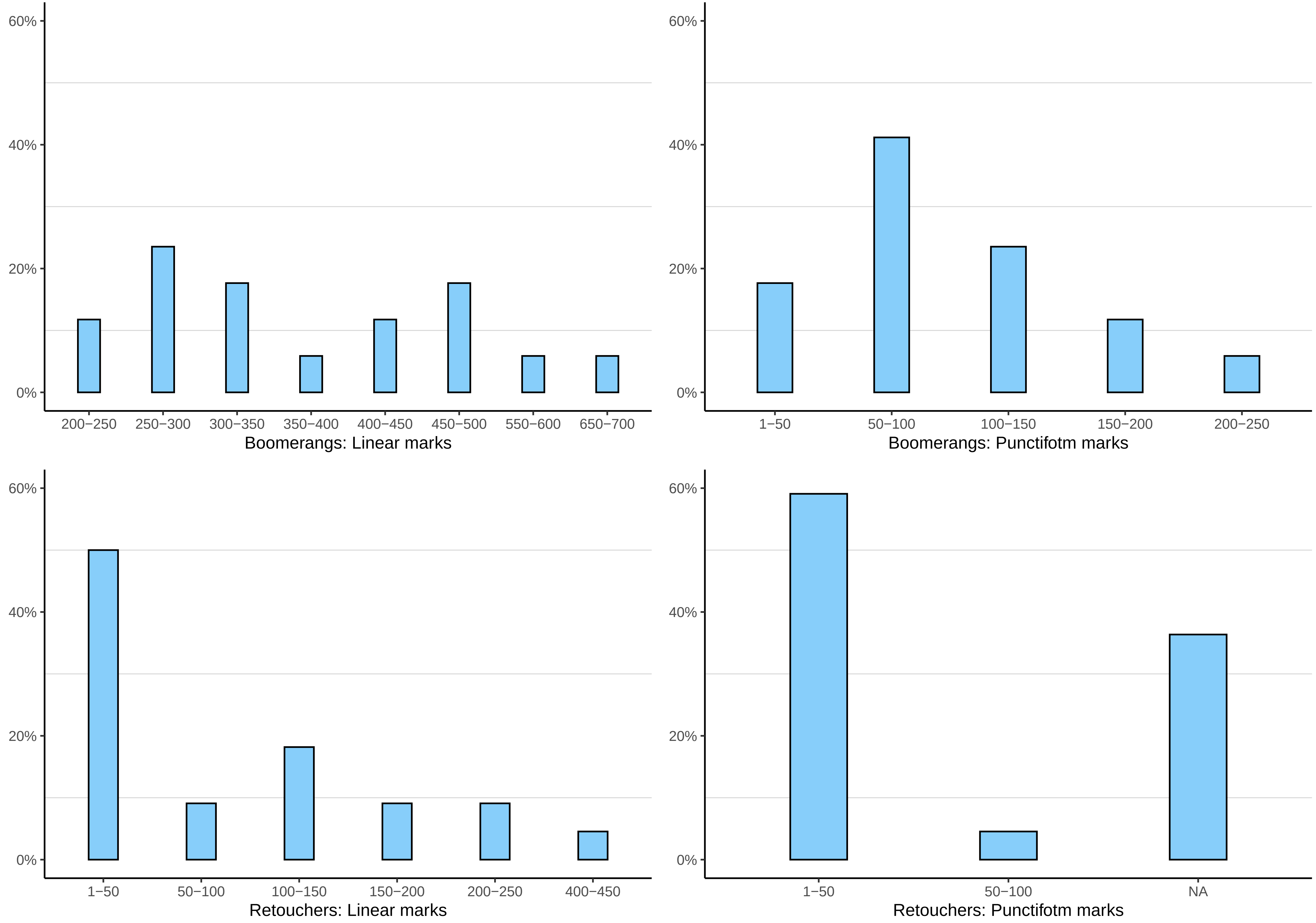

60%
40%
20%
0%
200−250
250−300
300−350
350−400
400−450
450−500
550−600
650−700
Boomerangs: Linear marks
1−50
50−100
100−150
150−200
200−250
Boomerangs: Punctifotm marks
1−50
50−100
100−150
150−200
200−250
400−450
Retouchers: Linear marks
1−50
50−100
NA
Retouchers: Punctifotm marks

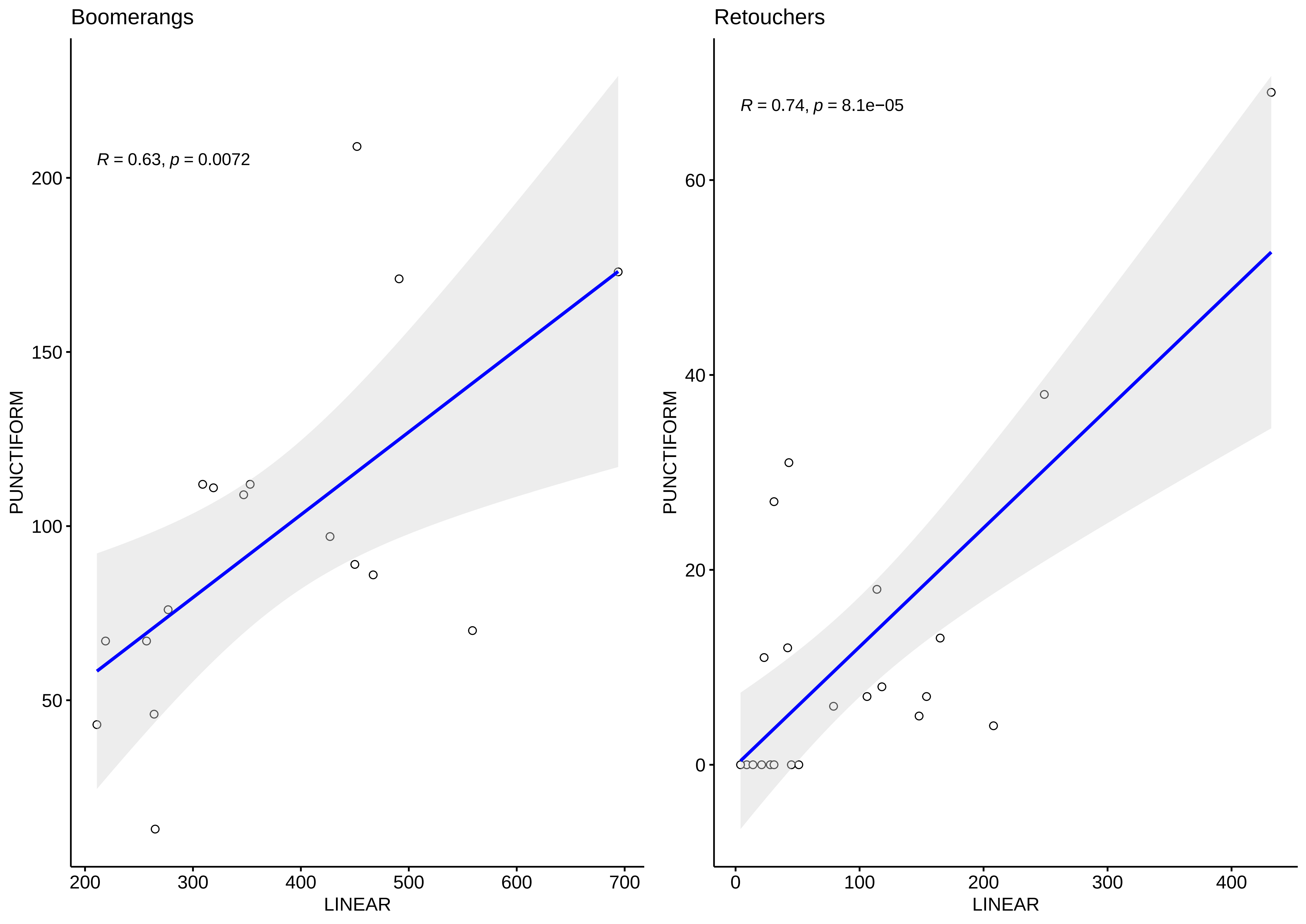
Boomerangs
R = 0.63, p = 0.0072
PUNCTIFORM
LINEAR
200
150
100
50
200
300
400
500
600
700
Retouchers
R = 0.74, p = 8.1e−05
PUNCTIFORM
LINEAR
60
40
20
0
0
100
200
300
400

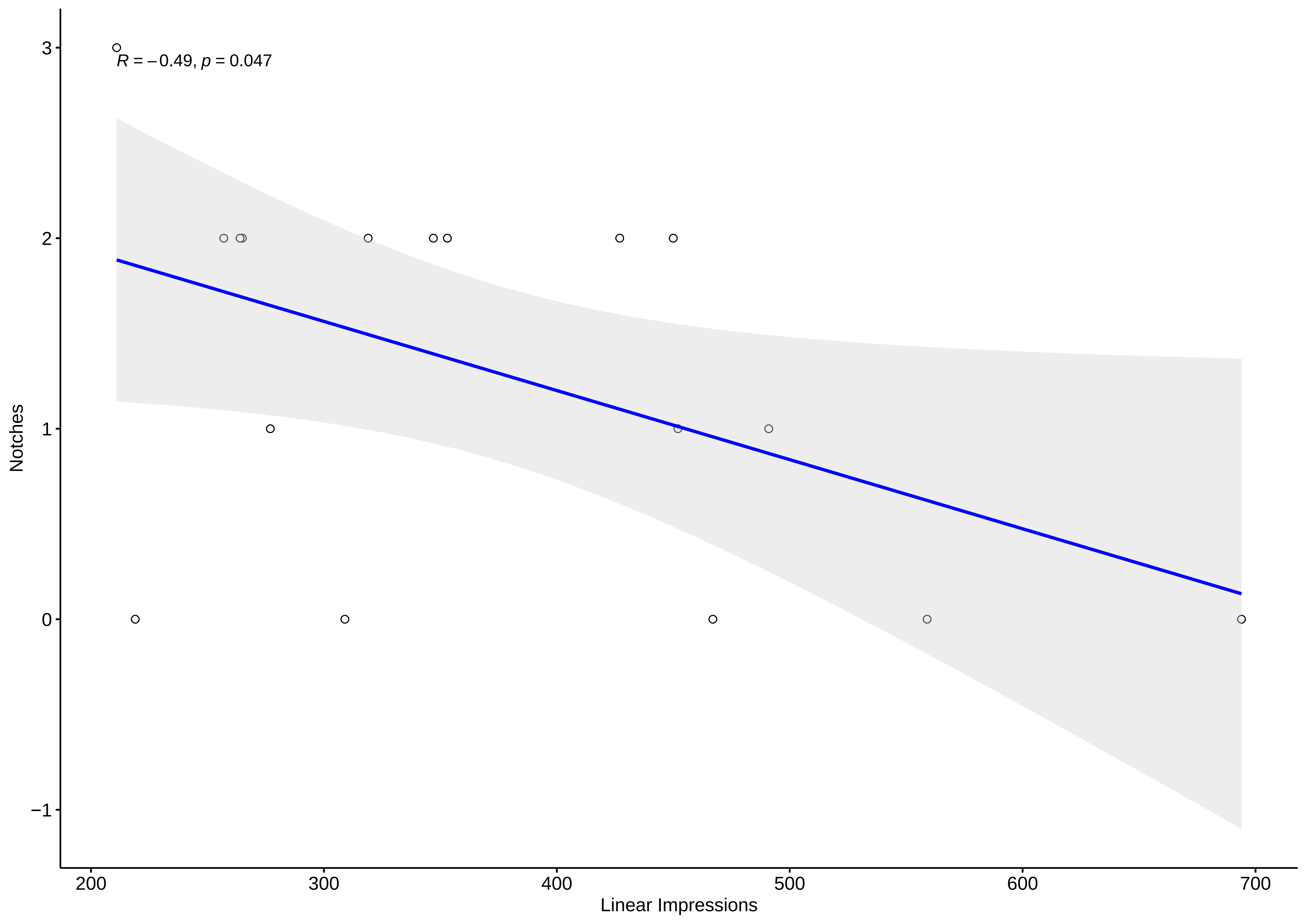

R = −0.49, p = 0.047
3
2
1
0
−1
Notches
200
300
400
500
600
700
Linear Impressions

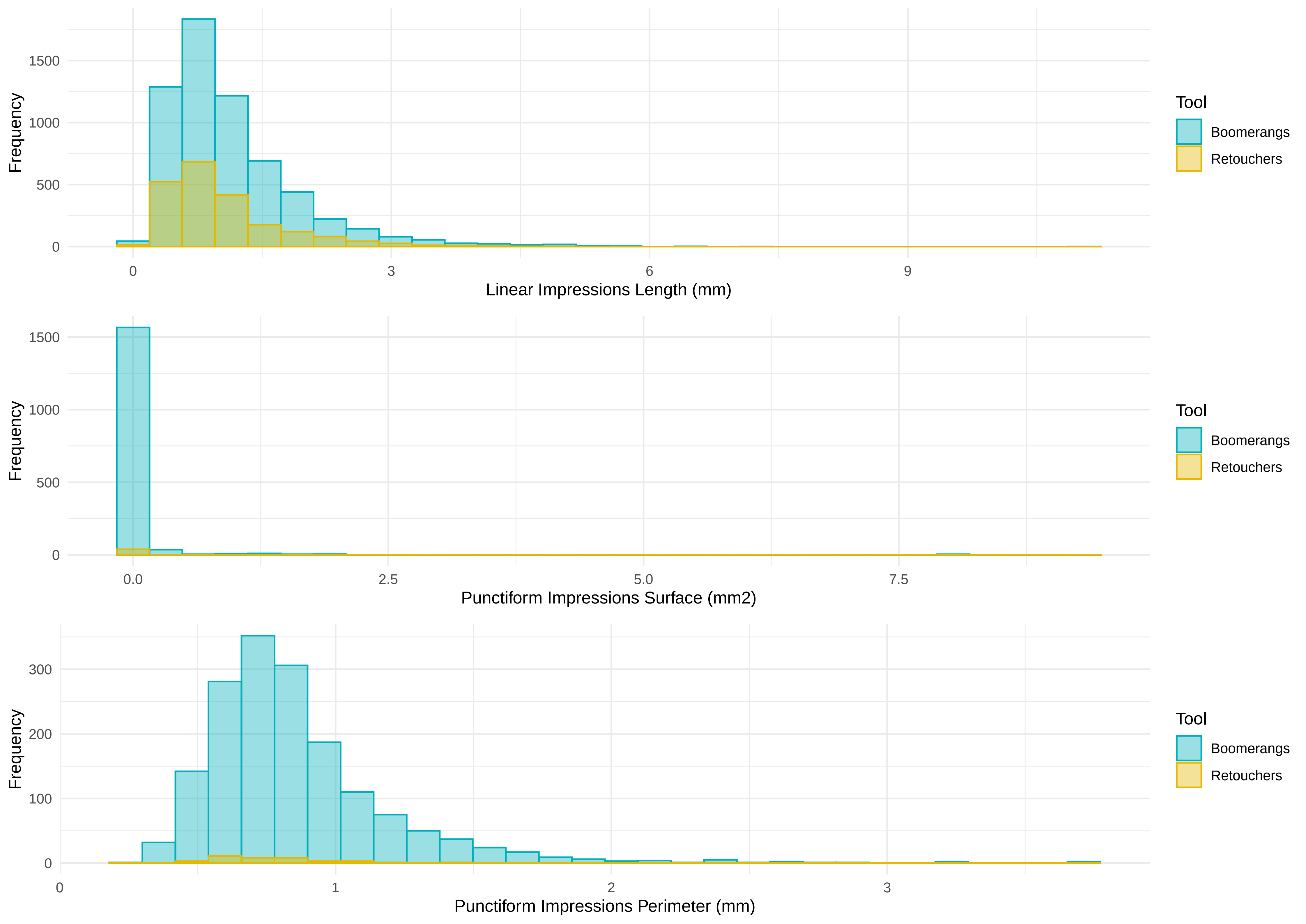

Frequency
1500
1000
500
0
0
3
6
9
Linear Impressions Length (mm)
Tool
Boomerangs
Retouchers
Frequency
1500
1000
500
0
0.0
2.5
5.0
7.5
Punctiform Impressions Surface (mm2)
Tool
Boomerangs
Retouchers
Frequency
300
200
100
0
0
1
2
3
Punctiform Impressions Perimeter (mm)
Tool
Boomerangs
Retouchers

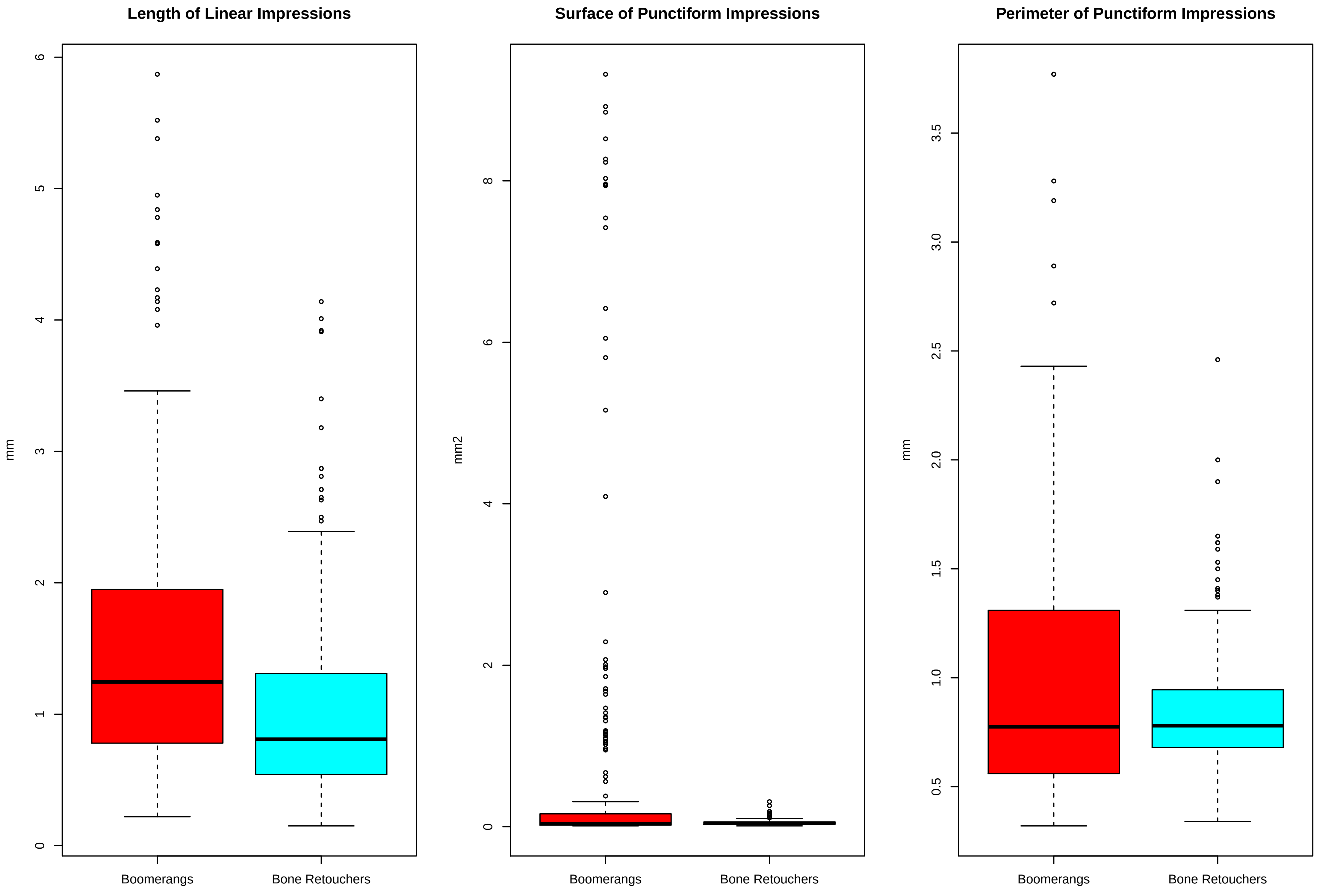
Length of Linear Impressions
Surface of Punctiform Impressions
Perimeter of Punctiform Impressions
mm
mm2
mm
0
1
2
3
4
5
6
0
2
4
6
8
0.5
1.0
1.5
2.0
2.5
3.0
3.5
Boomerangs
Bone Retouchers
Boomerangs
Bone Retouchers
Boomerangs
Bone Retouchers

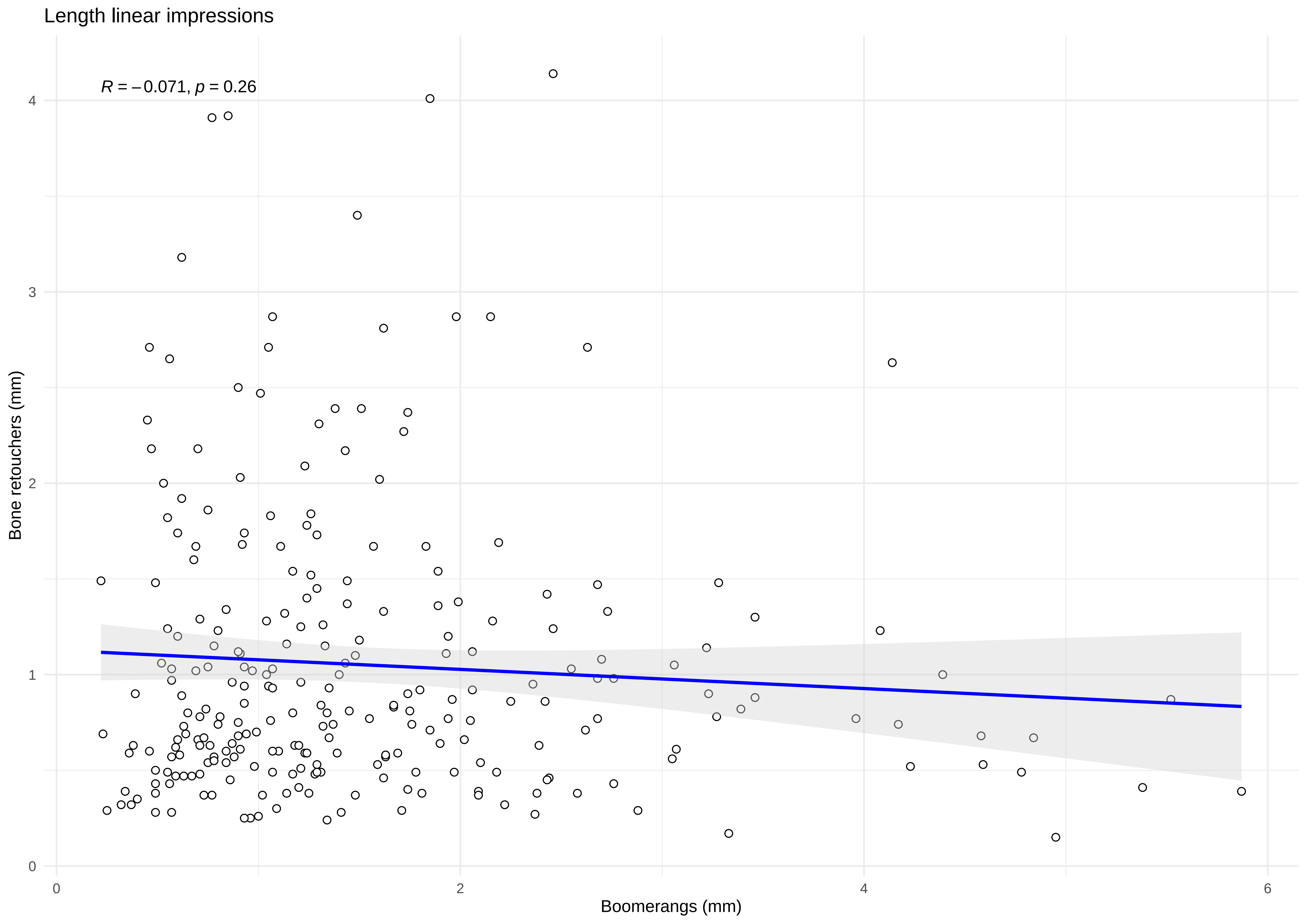

Length linear impressions
R = −0.071, p = 0.26
Bone retouchers (mm)
Boomerangs (mm)
0
1
2
3
4
0
2
4
6

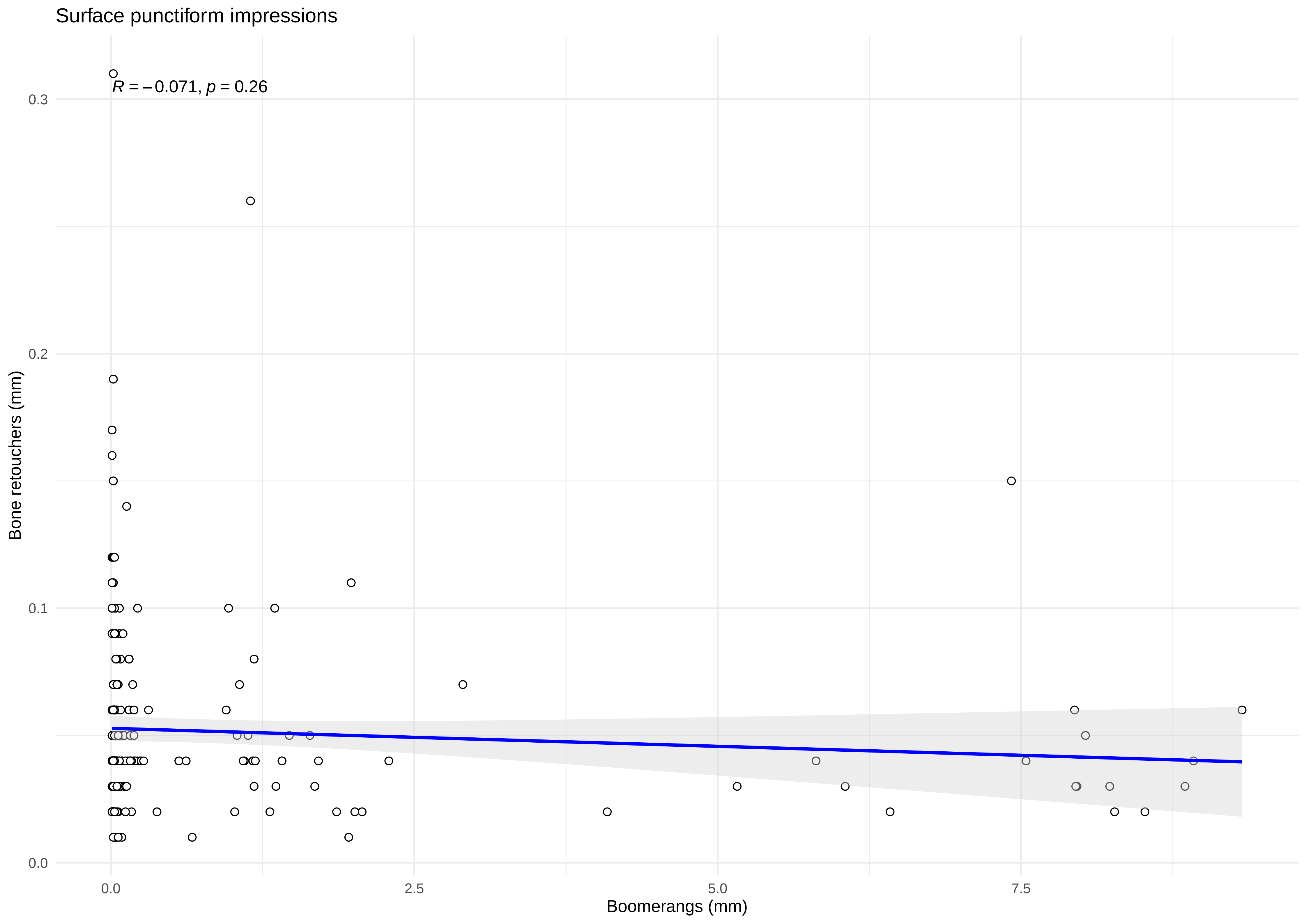

Surface punctiform impressions
R = −0.071, p = 0.26
0.3
0.2
0.1
0.0
Bone retouchers (mm)
0.0
2.5
5.0
7.5
Boomerangs (mm)

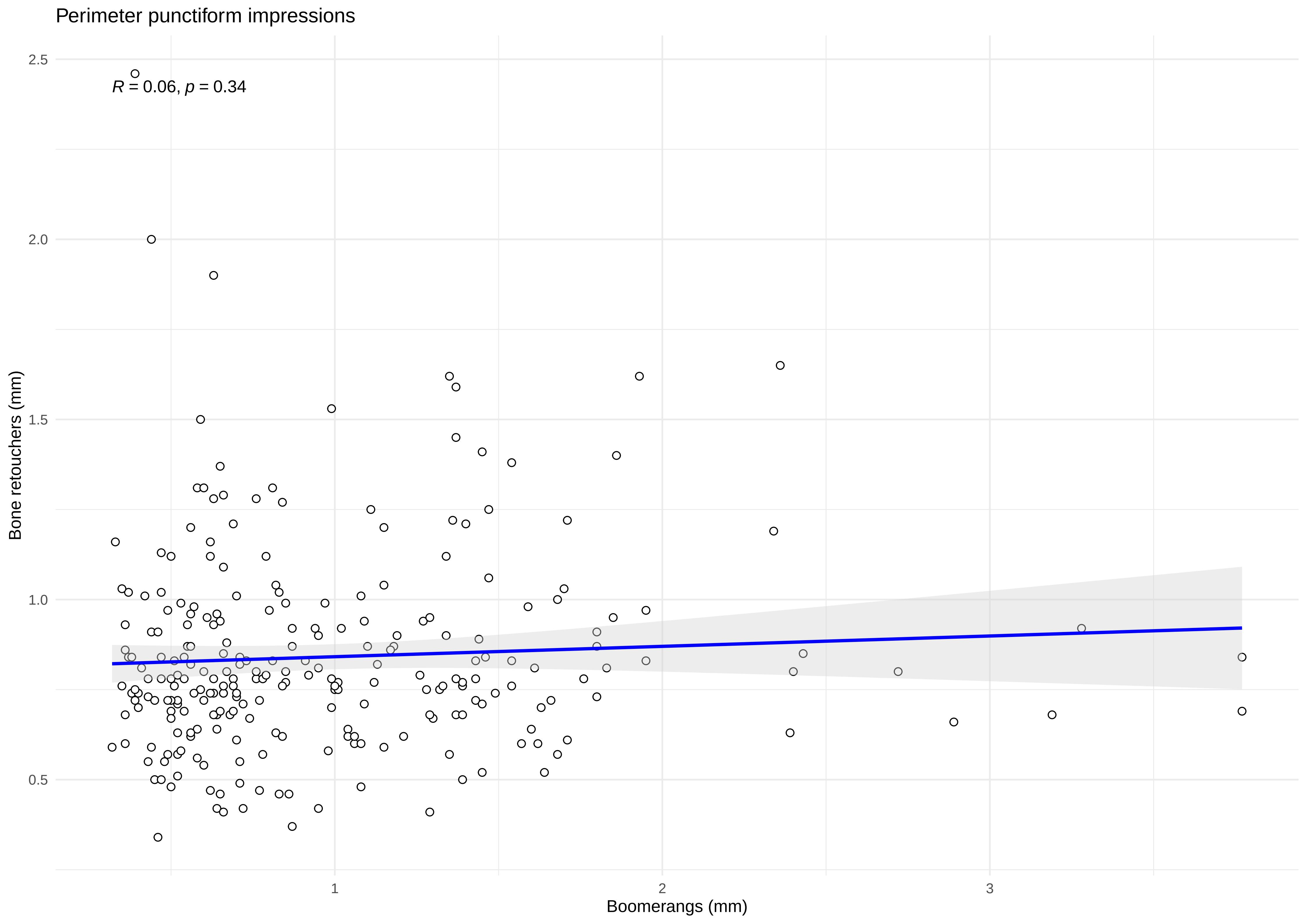

Perimeter punctiform impressions
R = 0.06, p = 0.34
2.5
2.0
1.5
1.0
0.5
Bone retouchers (mm)
1
2
3
Boomerangs (mm)

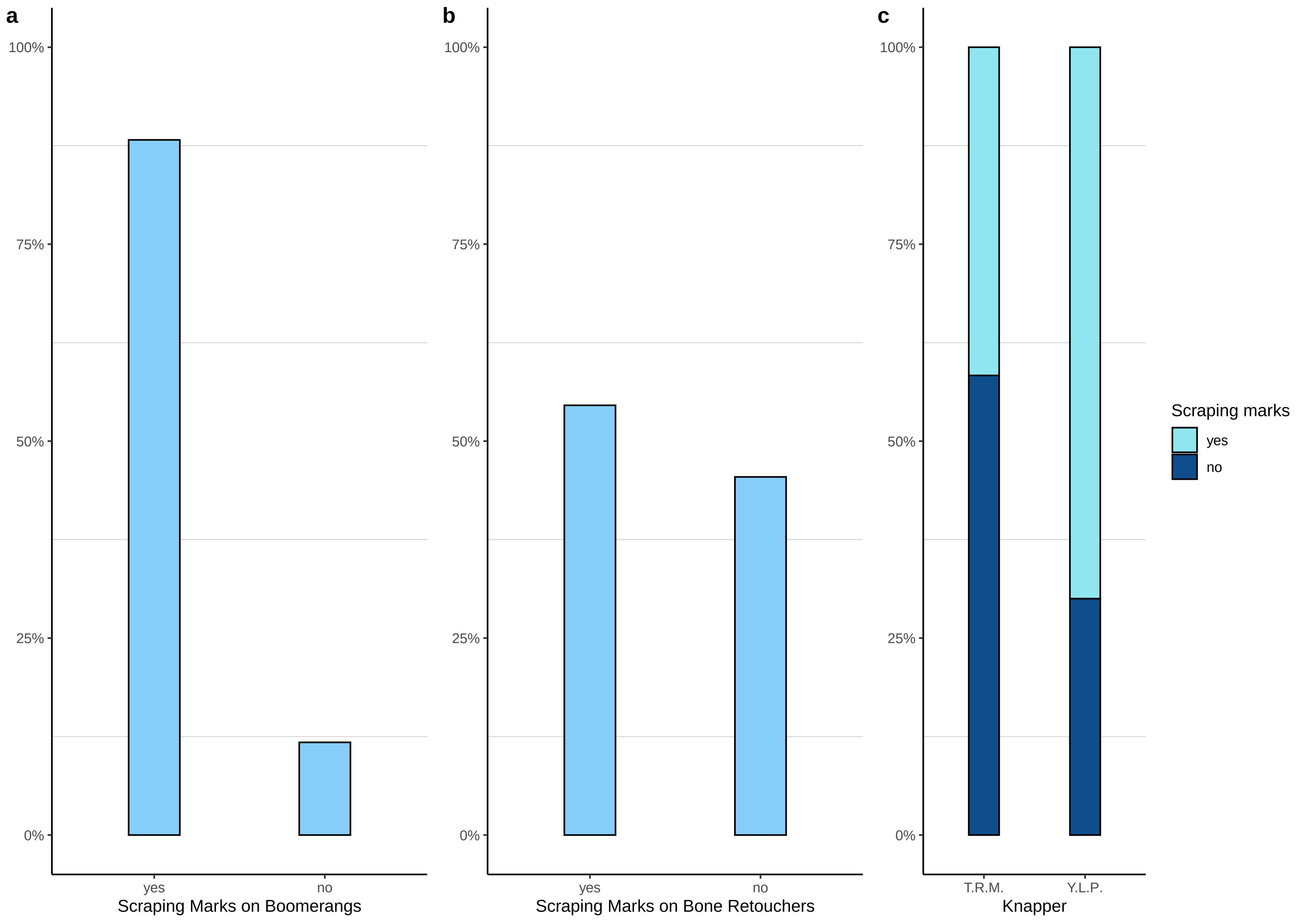

a
b
c
100%
75%
50%
25%
0%
yes
no
Scraping Marks on Boomerangs
Scraping Marks on Bone Retouchers
T.R.M.
Y.L.P.
Knapper
Scraping marks
yes
no
