# Supplementary material for: Beyond the main function: An experimental study of the use of hardwood boomerangs in retouching activities
Source: PLoS One. 2022 Aug 16;17(8):e0273118. doi: 10.1371/journal.pone.0273118 (PMC9380927; doi:10.1371/journal.pone.0273118)
Supplement: S1 Table — Among them, 32 were selected to be retouched (see Table 3 within text). ‘NA’ stands for ‘Not Applicable’. (PDF) [file pone.0273118.s007.pdf]

ID	Raw material	Length (mm)	Width (mm)	Thickness (mm)	Weight (g)	Proximal cross-section	Distal cross-section	Right cross-section	Left cross-section	Proximal angle (°)	Distal angle (°)	Right angle (°)	Left angle (°)	Cortex
**F01**	cobble_2	53.5	56.3	11.1	42.1	abrupt	plan-convex	bi-plan	abrupt	>90	25	25	>90	<20%
**F02**	cobble_2	37	58.1	13	32.4	abrupt	plan-convex	abrupt	bi-plan + plano-concave	90	55	90	50	none
**F03**	cobble_2	89	60.1	22.8	56.7	abrupt	abrupt	bi-plan	plano-convex	>90	90	55	50	>50%
**F04**	cobble_2	49.8	37.6	9.4	20.7	abrupt	bi-plan	bi-plan	bi-plan	>90	50	25	50	>50%
**F05**	cobble_2	44	29.6	12.5	17.4	abrupt	concave-convex	plan-convex	bi-plan	90	30	40	20	20%
**F06**	cobble_2	82.8	61.7	20.2	78.5	abrupt	plan-convex	plan-concave	bi-plan	>90	30	40	40	20%
**F07**	cobble_2	68.9	64.6	18.1	70.9	abrupt	plan-convex	convex-concave	abrupt	>90	40	30	>90	20%
**F08**	cobble_2	76.1	42.5	13.1	28.8	abrupt	bi-plan	plan-convex	concave-plan	>90	40	50	30	>50%
**F09**	cobble_2	29.6	42.3	13.6	9.5	abrupt	bi-plan	convex-plan	bi-plan	80	30	20	40	none
**F10**	cobble_2	55	46.4	14	35	abrupt	bi-plan	bi-plan	bi-plan	>90	65	35	60	none
**F11**	cobble_2	39.6	35.2	9.9	13.5	abrupt	bi-plan	bi-plan	abrupt	>90	35	20	30	none
**F12**	cobble_2	41.7	73.6	21.7	54.7	abrupt	bi-plan	bi-convex	bi-convex	>90	40	60	60	>50%
**F13**	cobble_2	82.5	80.5	21.8	81.1	abrupt	concave-convex	plan-convex	bi-plan	>90	30	75	65	>50%
**F14**	cobble_2	72.7	62.9	15.9	66.6	abrupt	plan-convex	bi-plan	plano-convex	>90	40	35	30	>50%
**F15**	cobble_2	35.6	22.8	6.2	6.4	abrupt	bi-plan	bi-plan	bi-plan	>90	40	25	15	none

ID	Raw material	Length (mm)	Width (mm)	Thickness (mm)	Weight (g)	Proximal cross-section	Distal cross-section	Right cross-section	Left cross-section	Proximal angle (°)	Distal angle (°)	Right angle (°)	Left angle (°)	Cortex
**F16**	cobble_2	59.9	52.5	14.2	32.7	abrupt	bi-plan	convex-plan	concave-plan	>90	30	45	25	none
**F17**	cobble_2	64.9	52.8	29.6	53.3	abrupt	bi-plan	bi-plan	bi-plan	>90	30	40	90	50%
**F18**	cobble_2	53.8	31.4	10.5	11.6	abrupt	bi-plan	bi-plan	bi-plan	>90	10	40	15	50%
**F19**	cobble_2	77.2	53.2	7.3	40.3	convex-concave	bi-plan	bi-plan	bi-plan	90	25	55	10	<20%
**F20**	cobble_2	31	32.3	13.6	11.1	abrupt	bi-plan	bi-plan	bi-plan	>90	25	60	45	none
**F21**	cobble_2	53.7	36.3	11.4	24.6	abrupt	plan-convex	bi-plan + plan-concave	bi-plan	>90	65	60	40	none
**F22**	cobble_2	62.7	26.6	11.4	19.5	abrupt	bi-plan	concave-plan	convex-plan	>90	40	45	60	<20%
**F23**	cobble_2	98.6	45.8	22.3	113.9	abrupt	plan-convex	plan-convex	plano-convex	>90	45	60	70	>50%
**F24**	cobble_2	73.3	47.1	18.3	56.9	abrupt	concave-plan	bi-plan	plano-concave	>90	35	50	60	>50%
**F25**	cobble_2	56	51.4	11.8	27.5	abrupt	convex-plan	convex-plan	convex-plan	>90	45	50	40	none
**F26**	cobble_2	89.6	71.6	17.7	142.2	abrupt	bi-plan	bi-plan	plan-concave	>90	75	50	50	<20%
**F27**	cobble_2	117.4	75.8	25.8	168.4	abrupt	plan-convex	bi-plan	bi-plan	>90	75	55	35	50%
**F28**	cobble_2	52.8	71.8	15.2	49.4	abrupt	bi-plan	bi-plan	bi-convex	>90	30	45	40	<20%
**F29**	cobble_2	78.8	41.4	15.5	63.8	abrupt	plan-convex	plan-convex	bi-plan	>90	45	60	40	20%
**F30**	cobble_2	92.4	67.3	24.3	133	abrupt	abrupt	plan-convex	bi-plan	>90	90	40	30	<20%
**F31**	cobble_2	65.4	58.1	12	48.6	abrupt	bi-convex	plan-convex	plano-convex	>90	35	70	50	<20%

ID	Raw material	Length (mm)	Width (mm)	Thickness (mm)	Weight (g)	Proximal cross-section	Distal cross-section	Right cross-section	Left cross-section	Proximal angle (°)	Distal angle (°)	Right angle (°)	Left angle (°)	Cortex
**F32**	cobble_2	53.9	52.4	13.5	38	abrupt	plan-convex	plan-convex	bi-plan	>90	45	90	40	>50%
**F33**	cobble_2	31.9	40	11.6	12.3	abrupt	concave-plan	convex-plan	plan-convex	>90	20	40	35	20%
**F34**	cobble_2	91.5	95	39	159.6	abrupt	plan-convex	plan-convex	concave-plan	>90	55	70	50	<20%
**F35**	cobble_2	61.4	58.3	28.6	93.6	abrupt	bi-plan	plan-convex	plano-concave	>90	55	60	40	50%
**F36**	cobble_2	109.6	54.2	21.9	118.2	abrupt	bi-convex	plan-convex	bi-plan	>90	>90	45	35	50%
**F37**	cobble_2	99	56.3	25.2	120.4	abrupt	bi-convex	bi-plan	concave-convex	>90	65	40	55-75	20%
**F38**	cobble_2	27.9	54.1	9.9	16	abrupt	convex-plan	bi-convex	bi-plan	90	85	35	30	>50%
**F39**	cobble_2	95.6	66.6	26.1	144.3	abrupt	bi-convex	convex-concave	plan-concave	>90	40	45	55	20%
**F40**	cobble_2	97.6	76.3	26.8	191.7	abrupt	plan-convex	bi-plan	plan-convex	90	35	30	75	>50%
**F41**	cobble_2	82	66.7	30.3	181	abrupt	bi-convex	bi-plan	convex-concave	>90	90	90	50	>20%
**F42**	cobble_2	94.7	75.8	33.9	252.8	abrupt	plan-convex	plan-convex	bi-plan	>90	55	65	90-55	>50%
**F43**	cobble_2	49.9	40.6	11	23.4	abrupt	convex-plan	bi-plan	convex-concave	>90	40	55	25	20%
**F44**	cobble_2	51.2	28	11	19.1	abrupt	abrupt	concave-convex	plan-concave	>90	>90	50	40	none
**F45**	cobble_2	41.4	19.4	7.3	4.7	abrupt	bi-plan	bi-plan	abrupt	90	35	25	45	none
**F46a**	cobble_2	66.4	30.4	11.7	22.3	abrupt	plan-convex	abrupt	concave-convex	>90	60	90	70	20%

ID	Raw material	Length (mm)	Width (mm)	Thickness (mm)	Weight (g)	Proximal cross-section	Distal cross-section	Right cross-section	Left cross-section	Proximal angle (°)	Distal angle (°)	Right angle (°)	Left angle (°)	Cortex
**F46b**	cobble_2	68.9	41.5	11.3	24.1	abrupt	bi-convex	plan-concave	concave-convex	90	75	>90	60	20%
**F47a**	cobble_2	86.7	37.4	13.7	43.9	abrupt	convex-plan	bi-plan	abrupt	>90	35	40	90	>20%
**F47b**	cobble_2	67.2	28.4	12.9	22.3	abrupt	abrupt	abrupt	plano-convex	90	90	90	50	>50%
**Debris_2**	cobble_2	-	-	-	61.9	-	-	-	-	-	-	-	-	-
**Core_2**	cobble_2	60.5	58.4	40.3	108.5	-	-	-	-	-	-	-	-	-
**F48**	cobble_1	76.6	68.6	20	98	abrupt	plan-convex	plan-convex	bi-plan	>90	20	40	45	50%
**F49**	cobble_1	93	53.9	14.7	64.5	abrupt	bi-convex	bi-plan	concave-convex	>90	30	25	25	20%
**F50**	cobble_1	136	118	24.4	429	abrupt	plan-convex	plan-convex	plano-convex	>90	55	70	65	50%
**F51**	cobble_1	100.5	78	20.9	138.8	abrupt	plan-convex	concave-convex	plano-convex	>90	20	25	55	>50%
**F52**	cobble_1	110.4	64.9	25	199.3	abrupt	abrupt	bi-plan	abrupt	90	>90	50	80	>20%
**F53**	cobble_1	91.9	67.4	34.6	145.2	abrupt	bi-plan	bi-plan	convex-plan	>90	75	80	30	<20%
**F54**	cobble_1	80.2	131	30.6	206.4	abrupt	plan-convex	concave-convex	bi-plan	80	55	75	60	>50%
**F55**	cobble_1	144	76.1	31	341.8	abrupt	plan-convex	plan-convex	plano-convex	90	75	70	70	>50%
**F56**	cobble_1	90.6	76.4	29.6	170.7	abrupt	bi-plan	bi-plan	bi-convex	>90	40	30	65	>20%
**F57**	cobble_1	92.3	95	28.9	181.5	abrupt	bi-plan	bi-plan	bi-plan	>90	50	35	65	20%
**F58**	cobble_1	83	79	29.6	170.4	abrupt	plan-convex	plan-convex	plan-concave	>90	35	65	65	50%
**F59**	cobble_1	81	74.9	20.8	117	abrupt	convex-plan	bi-plan	bi-plan	>90	25	20	75	20%

ID	Raw material	Length (mm)	Width (mm)	Thickness (mm)	Weight (g)	Proximal cross-section	Distal cross-section	Right cross-section	Left cross-section	Proximal angle (°)	Distal angle (°)	Right angle (°)	Left angle (°)	Cortex
**F60**	cobble_1	87	84.1	25.1	155.3	abrupt	bi-plan	convex-concave	plan-concave	>90	20	65	75	20%
**F61**	cobble_1	86.3	83.8	20.7	134.1	abrupt	convex-plan	plan-convex	bi-plan	>90	65	75	50	>50%
**F62**	cobble_1	93	74.9	49.4	274.6	abrupt	bi-plan	bi-plan	plan-concave	90	50	40	70	<20%
**F63**	cobble_1	47	54.6	24.9	52.8	plan-concave	bi-plan	bi-plan	concave-plan	70	25	40	35	none
**F64**	cobble_1	31.2	41.3	11.7	17.8	abrupt	abrupt	plan-concave	bi-plan	>90	90	65	75	none
**F65**	cobble_1	68.2	50.8	21.4	74.3	abrupt	concave-plan	convex-concave	convex-concave	>90	70	60	30	>20%
**F66**	cobble_1	70.6	55.7	17.4	48.5	abrupt	plan-convex	bi-plan	plan-convex	>90	20	15	40	<20%
**F67**	cobble_1	56.3	17.1	7.9	8.5	abrupt	abrupt	convex-concave + plan-convex	bi-plan	>90	90	40	35	<20%
**F68**	cobble_1	87.3	44.9	16.3	44.5	abrupt	abrupt	bi-plan	bi-plan	>90	90	35	30	none
**F69**	cobble_1	44.7	81.6	23.9	78.7	plan-concave	bi-plan	abrupt	plan-convex	80	50	90	30	>50%
**F70**	cobble_1	50	45.6	12	25.5	abrupt	abrupt	bi-plan	abrupt	>90	90	60	85	none
**F71**	cobble_1	56.3	53.7	22.7	69.9	abrupt	convex-concave	bi-plan	bi-plan	>90	55	75	30	20%
**F72**	cobble_1	32.7	45.7	9.7	19.9	abrupt	abrupt	abrupt	convex-concave	>90	90	>90	15	<20%
**F73**	cobble_1	104	40.6	14.1	62.5	abrupt	convex-plan	bi-plan	bi-plan	>90	75	25	30	20%

ID	Raw material	Length (mm)	Width (mm)	Thickness (mm)	Weight (g)	Proximal cross-section	Distal cross-section	Right cross-section	Left cross-section	Proximal angle (°)	Distal angle (°)	Right angle (°)	Left angle (°)	Cortex
**F74**	cobble_1	66.4	68.8	24.9	115.5	plano-convex	concave-plan	plan-convex	bi-plan	90	50	80	45	50%
**F75**	cobble_1	69.5	39.7	18.6	42.8	plan-convex	bi-plan	plan-convex	bi-plan	65	55	65	30	>20%
**F76**	cobble_1	69.5	66.5	24.4	112	abrupt	bi-plan	bi-plan	bi-plan	>90	50	35	40	<20%
**F77**	cobble_1	55.2	77.7	14.1	62.2	abrupt	bi-plan	bi-plan	bi-plan	90	40	55	40	<20%
**F78**	cobble_1	60	47.3	16.4	70.7	abrupt	plan-convex	bi-plan	plan-concave	90	45	35	65	>50%
**F79**	cobble_1	62.7	45.2	20.1	63.2	abrupt	plan-convex	abrupt	plan-concave	>90	65	>90	35	20%
**F80**	cobble_1	89.2	58.1	20.7	102.4	abrupt	bi-plan	abrupt	plan-convex	>90	60	90	60	<20%
**F81**	cobble_1	60.7	46.4	17.3	42.9	abrupt	plan-convex	bi-plan	plan-concave	>90	60	65	35	<20%
**F82**	cobble_1	58.7	30.6	6.4	12.7	abrupt	bi-plan	bi-plan	bi-plan	>90	20	50	30	<20%
**F83**	cobble_1	71.3	26.4	15.6	22.6	abrupt	bi-plan	bi-plan	bi-plan	>90	50	35	40	none
**F84**	cobble_1	55.6	65	19.1	28.5	abrupt	bi-plan	bi-plan	abrupt	>90	45	35	90	none
**F85**	cobble_1	58.5	40	13.2	17.3	plan-convex	bi-plan	concave-convex	bi-plan	70	30	45	20	50%
**F86**	cobble_1	53.8	31.8	12.8	18.2	abrupt	plan-convex	bi-plan	abrupt	90	55	25	75	20%
**F87**	cobble_1	59.2	52.4	24.9	49.8	abrupt	bi-plan	abrupt	bi-plan	>90	25	>90	60	<20%
**F88**	cobble_1	88	72.3	13.5	59.3	abrupt	plan-convex	bi-plan	bi-plan	90	60	70	20	<20%
**F89**	cobble_1	75.7	43.6	22.7	43.4	abrupt	bi-plan	plan-convex	bi-plan	>90	35	30	30	50%
**F90**	cobble_1	64	48.6	18.2	55.8	abrupt	bi-plan	bi-plan	bi-convex	>90	20	65	50	>20%
**F91**	cobble_1	91.9	49	91.5	60.2	abrupt	bi-plan	bi-plan	bi-plan	>90	40	25	20	50%

ID	Raw material	Length (mm)	Width (mm)	Thickness (mm)	Weight (g)	Proximal cross-section	Distal cross-section	Right cross-section	Left cross-section	Proximal angle (°)	Distal angle (°)	Right angle (°)	Left angle (°)	Cortex
**F92**	cobble_1	38.4	33.5	8	14.9	abrupt	bi-plan	plan-convex	bi-plan	>90	30	50	45	20%
**F93**	cobble_1	36.7	27.2	5.3	5.5	abrupt	bi-plan	bi-plan	abrupt	90	20	40	90	<20%
**F94**	cobble_1	55.8	39.6	9	15.5	abrupt	plan-convex	plan-concave	plan-convex	90	45	40	60	<20%
**F95**	cobble_1	37.7	26	3.7	3.6	convex-concave	bi-plan	bi-plan	bi-plan	20	10	15	25	none
**F96**	cobble_1	22	50.7	7	9.3	bi-convex	bi-convex	abrupt	bi-plan	65	75	80	40	none
**F97**	cobble_1	57.6	32	15.4	28.6	abrupt	plan-convex	bi-plan	plan-convex	>90	60	65	70	50%
**F98**	cobble_1	52.5	40.2	19.4	39.5	abrupt	bi-plan	abrupt	plan-convex	>90	25	90	40	<20%
**F99**	cobble_1	56	27.1	8.9	9	concave-plan	bi-plan	bi-plan	bi-plan	40	30	35	25	none
**F100**	cobble_1	51.4	64	21.2	51	abrupt	bi-plan	bi-plan	abrupt	>90	35	30	>90	none
**F101**	cobble_1	25.4	50.2	7.6	8.8	plan-convex	plan-convex	bi-plan	bi-plan	85	35	35	50	50%
**F102**	cobble_1	54.7	32	10.5	14	abrupt	bi-plan	bi-plan	bi-plan	>90	20	25	60	none
**F103**	cobble_1	49.2	27.5	7.2	11.6	abrupt	bi-convex	bi-plan	bi-plan	>90	70	75	30	20%
**F104**	cobble_1	59.1	49.7	15	34.3	abrupt	bi-plan	bi-plan	bi-plan	>90	15	20	65	50%
**F105**	cobble_1	57.4	42.3	10.8	28	abrupt	bi-plan	bi-plan	abrupt	>90	40	20	>90	none
**F106**	cobble_1	37.8	26.8	5.8	4.9	abrupt	bi-plan	bi-plan	bi-plan	85	20	15	30	<20%
**F107**	cobble_1	58.9	37.2	10.4	20.3	abrupt	bi-convex	bi-plan	bi-plan	90	65	25	15	20%
**F108**	cobble_1	39.6	39.8	13	12	convex-plan	abrupt	concave-plan	bi-plan	65	>90	45	30	none

ID	Raw material	Length (mm)	Width (mm)	Thickness (mm)	Weight (g)	Proximal cross-section	Distal cross-section	Right cross-section	Left cross-section	Proximal angle (°)	Distal angle (°)	Right angle (°)	Left angle (°)	Cortex
**F109**	cobble_1	38	22	3.7	5	bi-plan	bi-convex	bi-plan	bi-plan	20	70	40	25	none
**F110**	cobble_1	38.6	38.8	11.6	11	abrupt	bi-plan	bi-plan	bi-plan	>90	25	40	15	none
**F111**	cobble_1	59.5	50	19.9	45.5	abrupt	bi-plan	bi-plan	plan-convex	>90	40	75	40	none
**F112**	cobble_1	31.7	45.5	8.3	13.3	abrupt	abrupt	abrupt	bi-plan	>90	90	90	30	none
**F113**	cobble_1	69.8	45.9	12.6	26.8	abrupt	plan-convex	plan-convex	bi-plan	>90	35	50	35	>20%
**core_1**	cobble_1	70.1	43.7	27.7	61.6	NA	NA	NA	NA	NA	NA	NA	NA	20%
**debris_1**	cobble_1	NA	NA	NA	91.2	NA	NA	NA	NA	NA	NA	NA	NA	none
