# Supplementary material for: Beyond the main function: An experimental study of the use of hardwood boomerangs in retouching activities
Source: PLoS One. 2022 Aug 16;17(8):e0273118. doi: 10.1371/journal.pone.0273118 (PMC9380927; doi:10.1371/journal.pone.0273118)
Supplement: S2 Table — (PDF) [file pone.0273118.s008.pdf]

**Bone ID**	**Species**	**Sex**	**Age**	**Skeletal element**	**Bone laterality**	**Bone state**	**Cleaning**				**Size**					**Breakage**						
							Defleshing	Tendons removal	Disarticulation	Periosteum removal	Length (cm)	Width (cm)	Thickness (cm)	Circumference (cm)	Weight (g)	Operator	Hammer	Anvil	Selected blanks	Discarded blanks	Total blanks	Notes
**B01**	*Bos taurus*	female	sub-adult	humerus	R	fresh		x		x	30	14	5	17	1377	E.F.M.	H1		2	10	12	periosteum removal info
**B02**	*Bos taurus*	female	sub-adult	radius/ulna	R	fresh		x		x	37	12	2.9	16	1109	T.D.	H2	x	1	9	10	flake used for separating ulna
**B03**	*Bos taurus*	female	sub-adult	tibia	L	fresh		x	x	x	34	14	3.2	19	1122	E.F.M.	H1	x	6	5	11	periosteum removal info
**B04**	*Bos taurus*	female	sub-adult	femur	L	frozen		x		x	35	15	4.3	16	1407	E.F.M.	H2		4	5	9	periosteum removal info
**B05**	*Bos taurus*	female	sub-adult	tibia	R	frozen		x			36	13.5	3.2	15	1120	T.D.	H2		4	5	9	
**B06**	*Bos taurus*	female	sub-adult	humerus	R	frozen		x			31	13	5.2	16	1300	E.F.M.	H1		2	6	8	cutting epiphysis
**B07**	*Bos taurus*	female	sub-adult	femur	R	frozen		x			40	16	4.4	16	1900	E.F.M.	H1		3	5	8	
**B08**	*Bos taurus*	female	sub-adult	radius/ulna	R	frozen		x		x	36	9	4.2	16	1100	Eva	H1	x	1	6	7	light periosteum removal; anvil used only occasionally
