# Supplementary material for: Beyond the main function: An experimental study of the use of hardwood boomerangs in retouching activities
Source: PLoS One. 2022 Aug 16;17(8):e0273118. doi: 10.1371/journal.pone.0273118 (PMC9380927; doi:10.1371/journal.pone.0273118)
Supplement: S1 File — In each template, the first picture is a photo of bones before the breaking; the second is a 360° view of the bone, with indication of the impact points (in chronological order) and the portion used for retouchers (in red); the third picture is a photo of the bone blanks selected to be used as retouchers (photo and drawings by E. F. Martellotta). To facilitate the identification of the selected blanks, each bone was divided in 20 portions following: Romandini M. Analisi archeozoologica, tafonomica, paleontologica e spaziale dei livelli Uluzziani e tardo-Musteriani della Grotta di Fumane (VR). Variazioni e continuità strategico-comportamentali umane in Italia Nord Occidentale: i casi di Grotta del Col della Stria. Dipartimento di Biologia ed Evoluzione. Università degli Studi di Ferrara. 2012. Available: https://iris.unife.it/handle/11392/2389242#.XaaWxugzaUk. (PDF) [file pone.0273118.s009.pdf]

BONE ID: B01

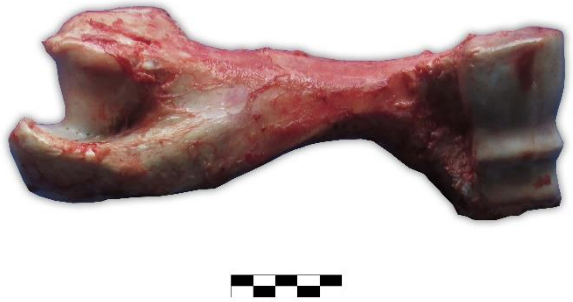

Operator: E.F.M. **Hammer**: H1 **Anvil**: no

Description:

The first four tentative of impact (impacts #1-4) were unsuccessful. Impact #5 creates a transversal fracture, with oblique angle, curved profile and denticulated edge. Impact #6 was unsuccessful. Impact #7 created a longitudinal fracture, with oblique angle, intermediate edge and a mainly smooth edge; from impact #7, the bone retoucher R21 was extracted by flexion, and it required the scraping of periosteum mixed with tendons to help the detachment. Impact #8 created a transversal fracture, with oblique angle, transversal profile and denticulated edge; after some scraping of periosteum/tendons, R22 was extracted by flexion. In general, some parts of the periosteum were lightly scraped to facilitate the fracture.

Obtained blanks: selected= 2 discarded= 10 (64.1 g) total= 12

Retouchers code: R21 **Bone Portion**: d17-d18

R22 d7-d8

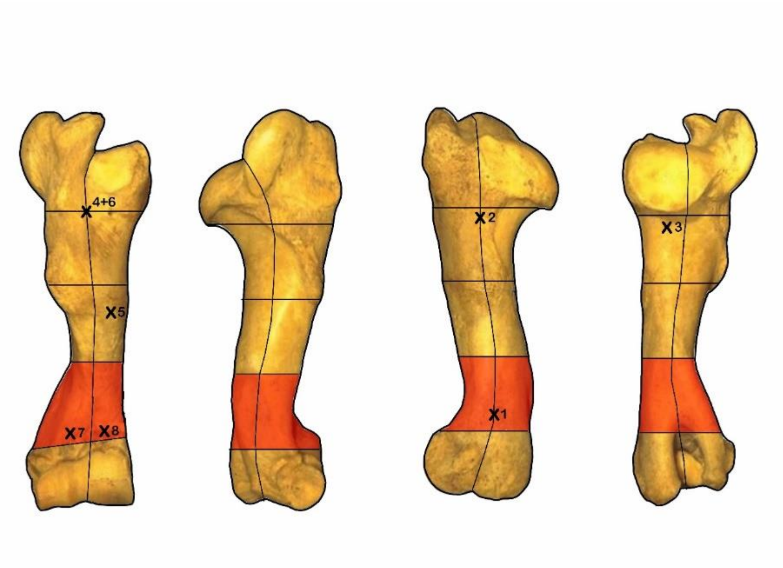
4+6
5
7
8
2
1
3

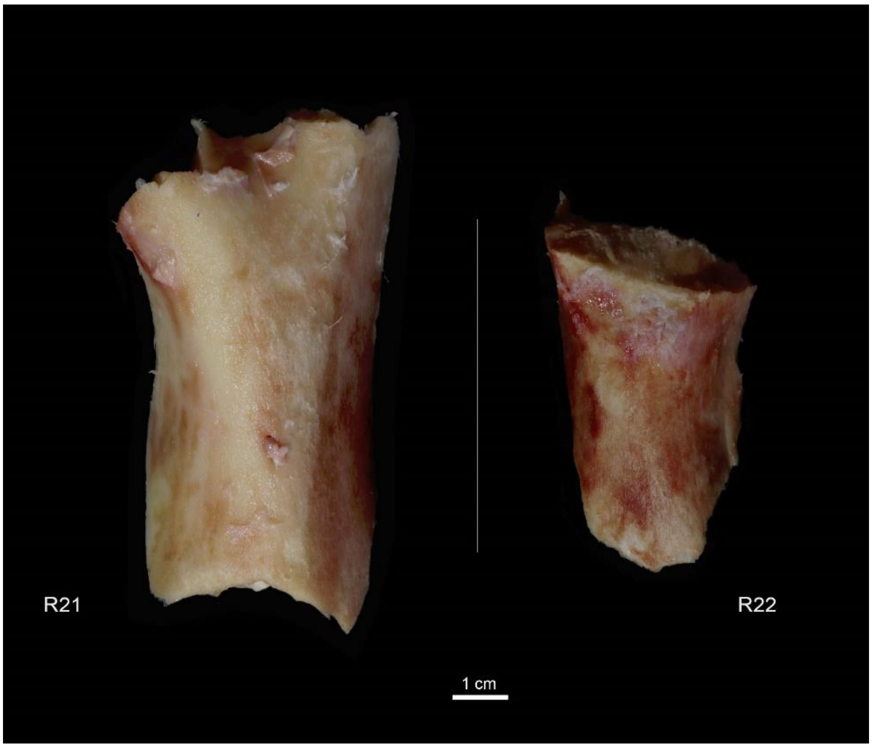
R21
R22
1 cm

BONE ID: B02

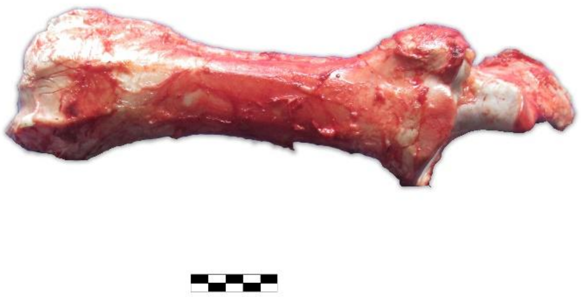

Operator: T.D. **Hammer**: H2 **Anvil**: yes

Description:

Impacts #1-4 were unsuccessful. The operator insisted for long time on impact #3, resulting in the collapse of the cortical bone, before passing to the next impact point. Impact #5 created a spiral fracture, with abrupt angle, intermediate profile and denticulated edge; as a result, the epiphysis was removed. Impact 6 resulted in a longitudinal fracture, with an oblique angle, a curved profile and a smooth edge; it resulted in R15. During the entire process, the operator used the flint flake trying to separate the ulna from the radium.

Obtained blanks: selected= 1 discarded= 9 (35.2 g) total= 10

Retouchers code: R15 **Bone Portion**: d15-d16

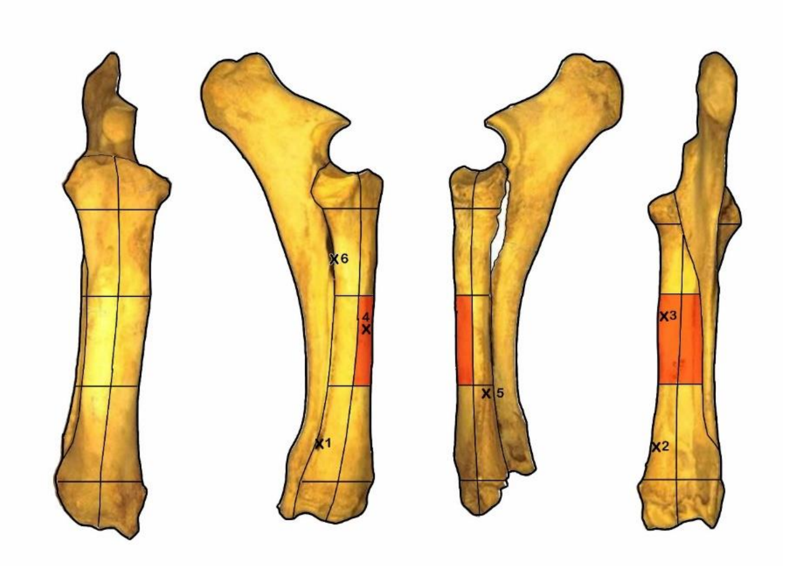

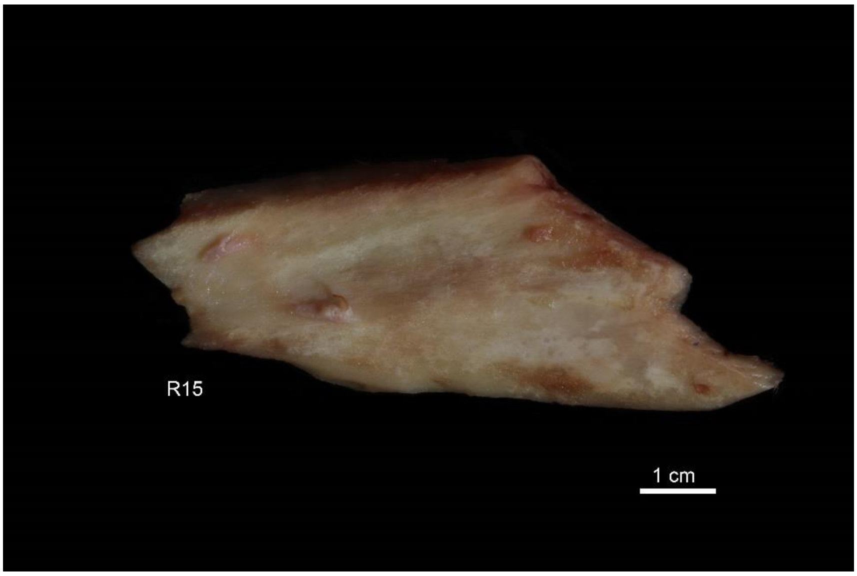
R15
1 cm

BONE ID: B03

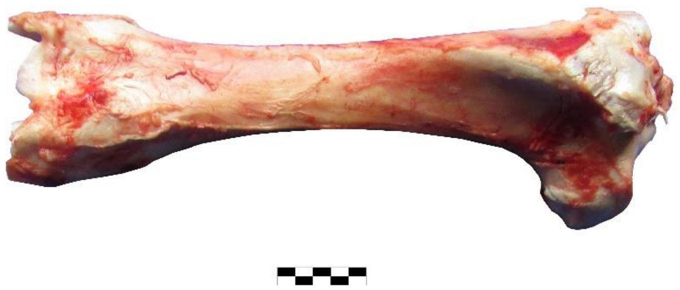

Operator: E.F.M. **Hammer**: H1 **Anvil**: yes

Description:

Impact #1 was unsuccessful and resulted in the collapse of the cortical bone. Impact #2 created a longitudinal fracture, with oblique angle, curved profile and smooth edge. Impact #3 resulted in a transversal fracture, with oblique angle, transversal profile and smooth edge; R1 was obtained from this fracture. Impact #4 created a longitudinal fracture with oblique angle, curved profile and smooth edge, resulting in R2. Impact #5 created two simultaneous longitudinal fractures in the diaphysis, both with an oblique angle, an intermediate profile and some smooth some denticulated edges. These two fractures resulted in R3, R4, R6 and R10.

Obtained blanks: selected= 6 discarded= 5 (24.1 g) total= 11

**Retouchers code**:	R1	**Bone Portion**:	d8
	R2		d7-d5
	R3		d4-e2
	R4		d13
	R6		d14
	R10		d3

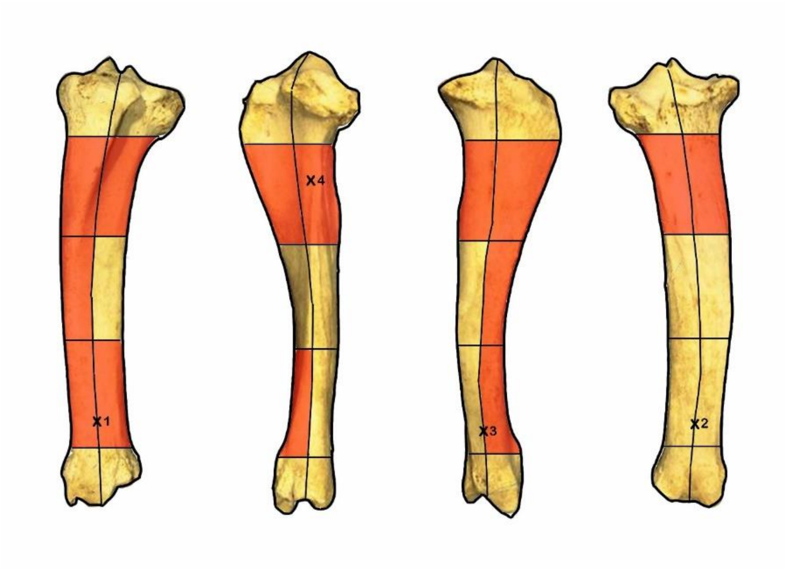
X4
X1
X3
X2

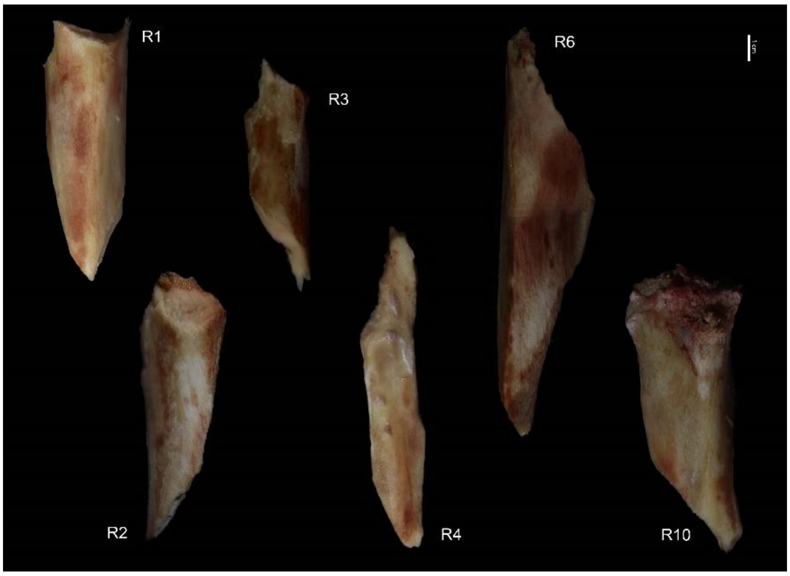
R1
R3
R6
R2
R4
R10

BONE ID: B04

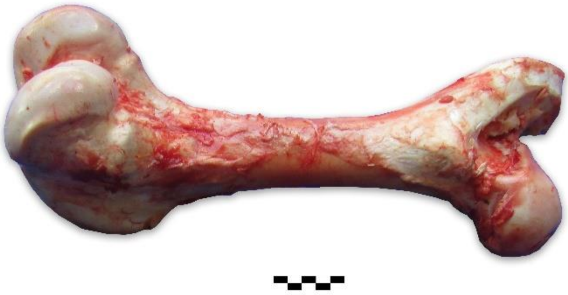

Operator: E.F.M. **Hammer**: H2 **Anvil**: no

Description:

Impact #1 created a transversal fracture, with oblique angle, intermediate profile and mostly smooth edge; it resulted in the detachment of the distal epiphysis. Another impact in the same point created a longitudinal fracture all along the diaphysis: it has oblique angle, curved profile and smooth edge. Towards the end the fracture slightly deviated laterally, resulting in the detachment of R16; the flexion of the other side of the fractured bone resulted in R17. Impact #2 created a transversal fracture, with abrupt angle, intermediate profile and denticulated edge; through flexion, it resulted in R18.

Obtained blanks: selected= 4 discarded= 5 (5.3 g) total= 9

**Retouchers code**	**Bone Portion**
R16	d16
R17	d13-d14
R18	d14-d3

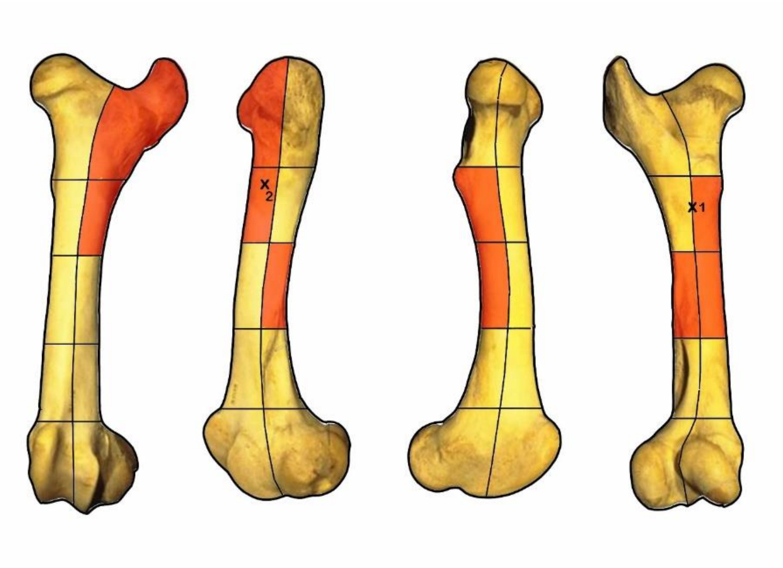
X
2
X1

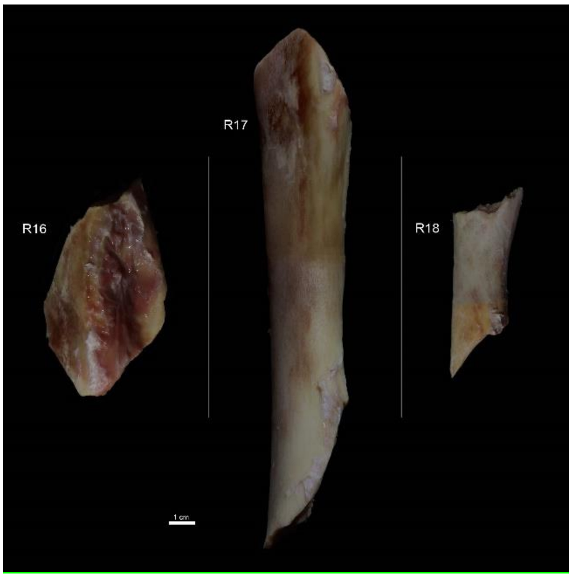
R17
R16
R18
1 cm

BONE ID: B05

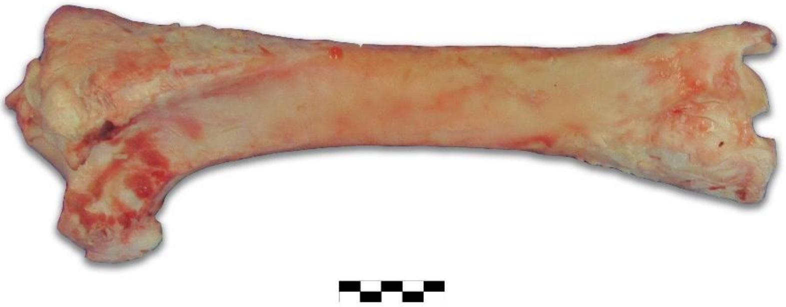

Operator: T.D. **Hammer**: H2 **Anvil**: no

Description:

Impact #1 was insisted and resulted in a light collapse of the cortical bone. At the end, however, it created a spiral fracture, with oblique angle, curved profile and smooth edge. Impacts #2-3 were unsuccessful. Impact #4 created a spiral fracture, with oblique angle, curved profile and smooth edge; through flexion, R5 was detached. Impact #5 created a transversal fracture, with abrupt angle, intermediate profile and denticulated edge; it resulted in R7. Impact #6 created a longitudinal fracture, with abrupt angle, curved profile and smooth edge; it resulted in R8 and R9.

Obtained blanks: selected= 4 discarded= 5 (12 g) total= 9

**Retouchers code**	**Bone Portion**
R5	d14
R7	d13-d14
R8	d18
R9	d17

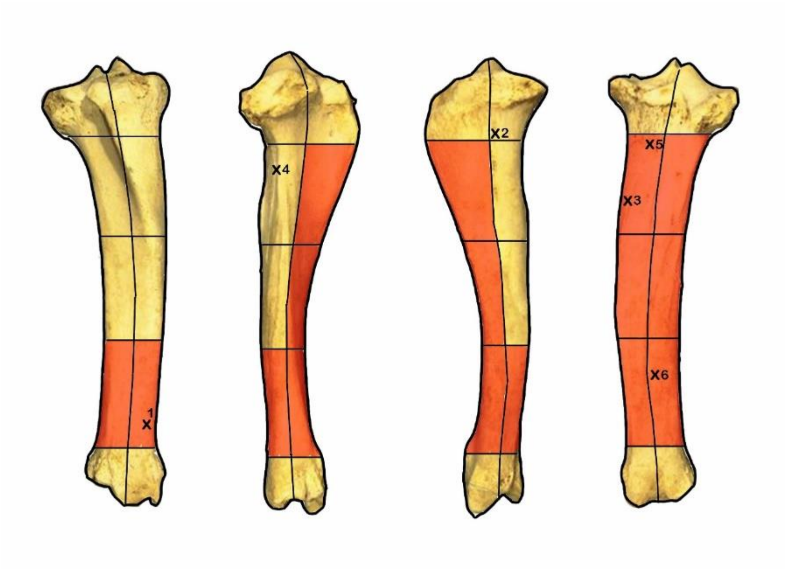
X1
X4
X2
X5
X3
X6

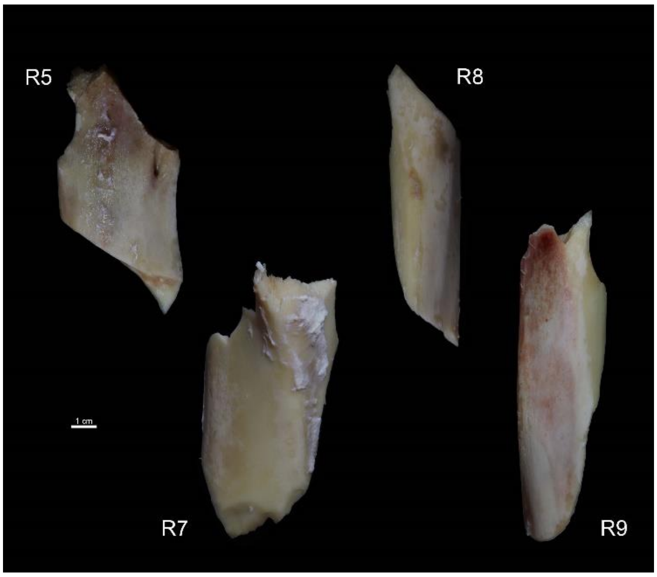
R5
R8
R7
R9
1 cm

BONE ID: B06

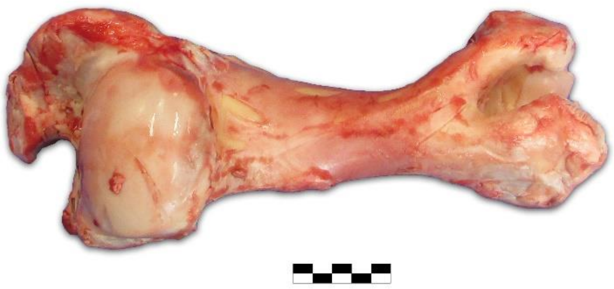

Operator: E.F.M. **Hammer**: H1 **Anvil**: no

Description:

Impact #1 resulted in a spiral fracture, with oblique angle, curved profile and denticulated edge. Impact #2 created a transversal fracture, with variable angle, intermediated profile and denticulated edge; R19 was detached. Impact #3 created a small, longitudinal fracture, with oblique angle, transverse profile, smooth edge; however, it did not fracture the epiphyseal part, which consisted in a very spongy tissue and several tendons. R20 was detached after strong flexion and cutting of the tendons.

Obtained blanks: selected= 2 discarded= 6 (19.3 g) total= 8

Retouchers code: R19 **Bone Portion**: d14-d3

R20 e1-e2-d3-d4

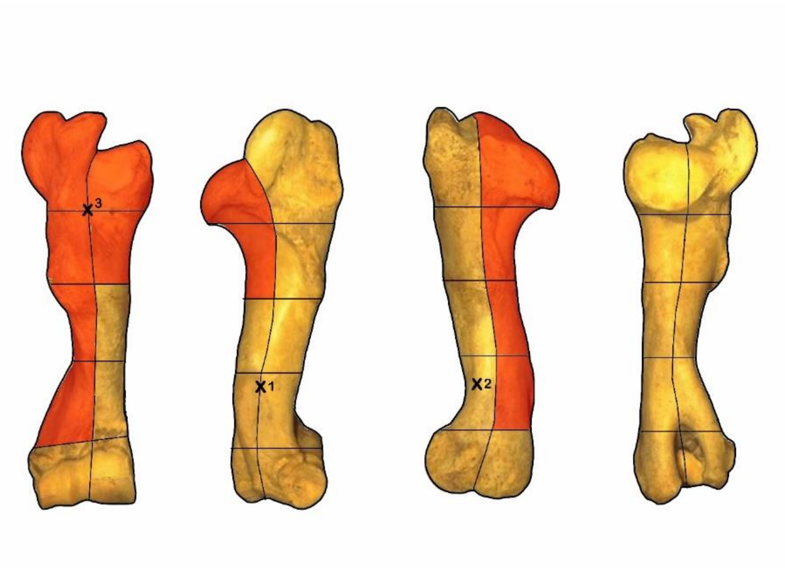
X3
X1
X2

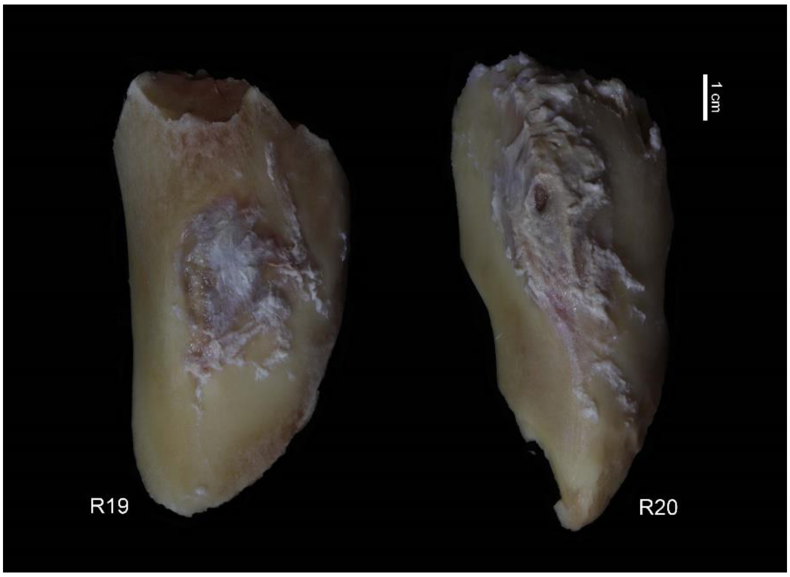
1 cm
R19
R20

BONE ID: B07

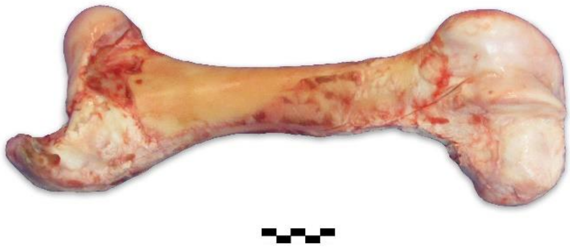

Operator: E.F.M. **Hammer**: H1 **Anvil**: no

Description:

Impact #1 created a spiral fracture, with oblique angle, curved profile and smooth edge. Impacts #2-3 were unsuccessful. Impact #4 created a transversal fracture in proximity of the proximal epiphysis, with abrupt angle, transversal profile and smooth edge, which resulted in the detachment of the distal epiphysis. Impact #5 created a longitudinal fracture of the diaphysis, which resulted in R11 and R12. Impact #6 a transversal fracture, with abrupt angle, transversal profile and denticulated edge; after flexion and scraping periosteum/tenons, R13 was detached.

Obtained blanks: selected= 3 discarded= 5 (36.9 g) total= 8

Retouchers code: R11 **Bone Portion**: d13-d14

R12 d3

R13 d4-e2

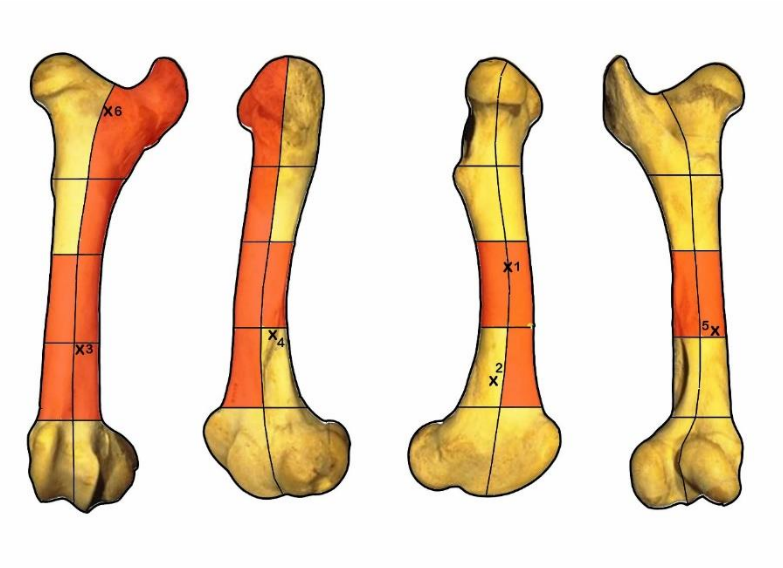
X6
X3
X4
X1
2
X
5X

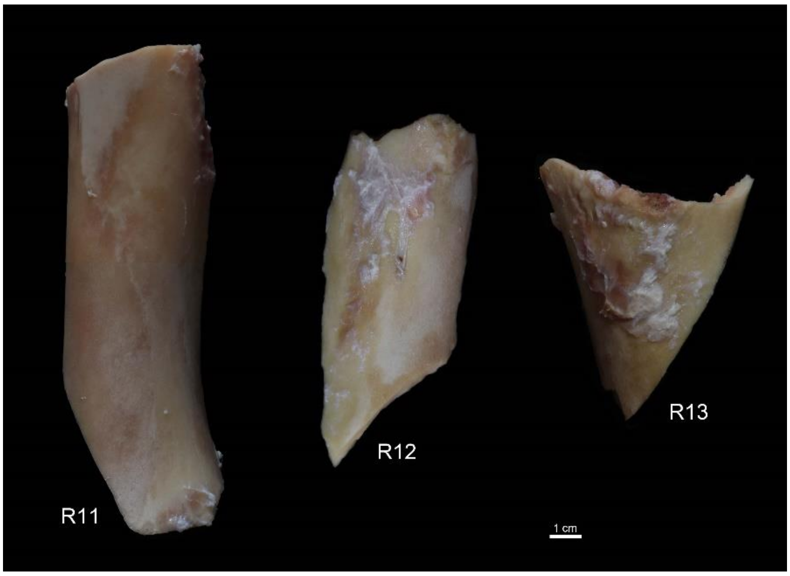
R11
R12
R13
1 cm

BONE ID: B08

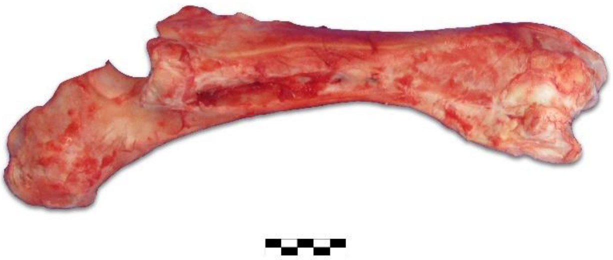

Operator: E.F.M. **Hammer**: H1 **Anvil**: yes

Description:

Impacts #1-4 were unsuccessful. Impact #5 created a spiral fracture only of the radius, and with an impact on the opposite side, the ulna collapsed. Impact #6 was unsuccessful. Impact #7 created a longitudinal fracture, with oblique angle, curved profile and sooth edge; after flexion, it resulted in R14.

Obtained blanks: selected= 1 discarded= 6 (41.2 g) total= 7

Retouchers code: R14 **Bone Portion**: d7

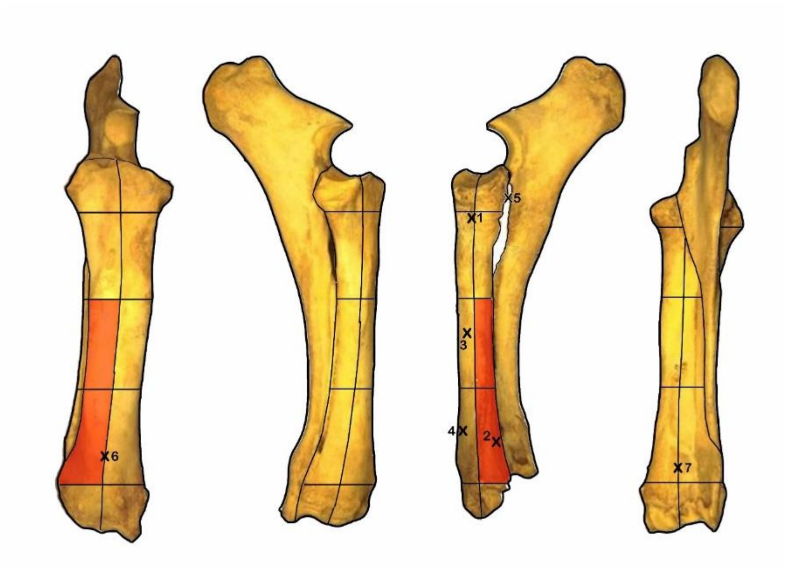

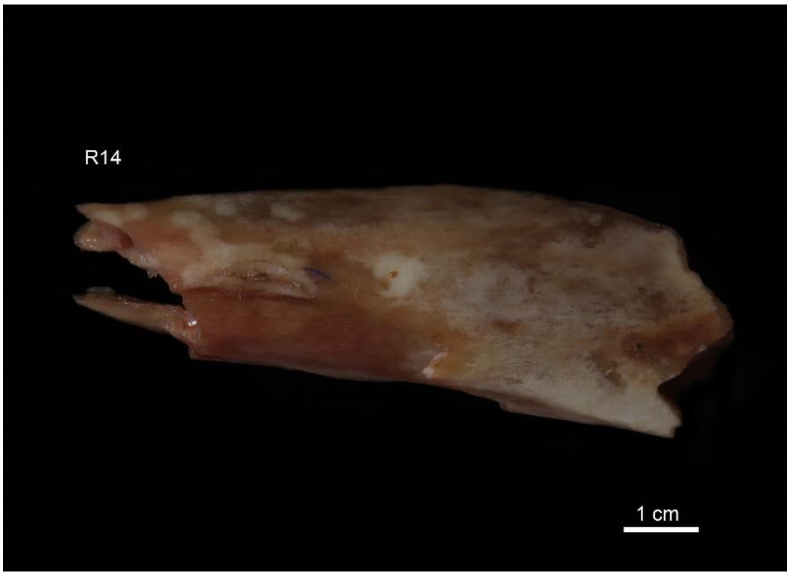
R14
1 cm
