# Supplementary material for: Beyond the main function: An experimental study of the use of hardwood boomerangs in retouching activities
Source: PLoS One. 2022 Aug 16;17(8):e0273118. doi: 10.1371/journal.pone.0273118 (PMC9380927; doi:10.1371/journal.pone.0273118)

S6 File: Details of retouch session 1. In each template, the first picture represents a 360° view of the bone retoucher and indication of the use area(s); the second picture is a photo of the retouched lithic flake. Retouchers described within the text (i.e., R6, R8, R12, R21) are excluded from this set (drawings and pictures by E. F. Martellotta).

Retoucher ID: R1

Knapper: Y.L.P.

Use area #1

N. of blows: 136

Notes: none

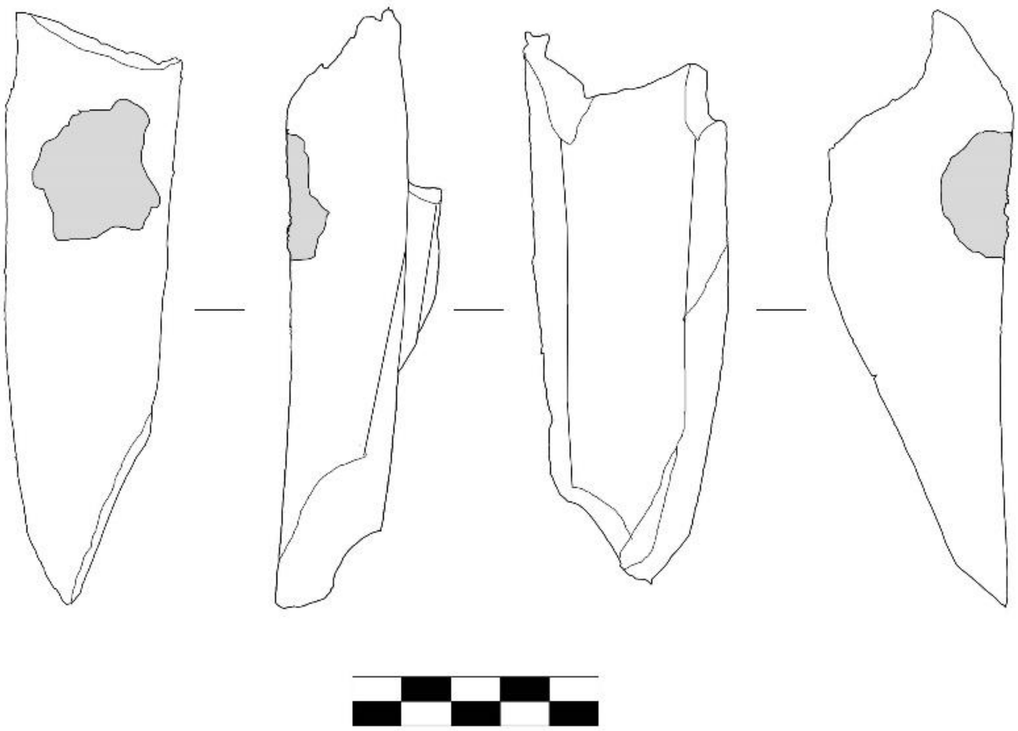

Flake code: F07

Description of the retouch:

Position: ☒direct ☒inverse ☐other

Delineation: ☒rectilinear ☒convex ☐concave ☐denticulated ☐other

Morphology: ☐scaled ☒stepped ☐parallel ☐sub-parallel

Localisation: ☒distal ☐mesial ☐proximal ☒right ☒left ☐basal

Distribution: ☒continuous ☐discontinuous ☒partial

Extent: ☒short ☐long ☐invasive

Angle: ☒abrupt (~90°) ☐semi-abrupt (~45°) ☐low (<30°)

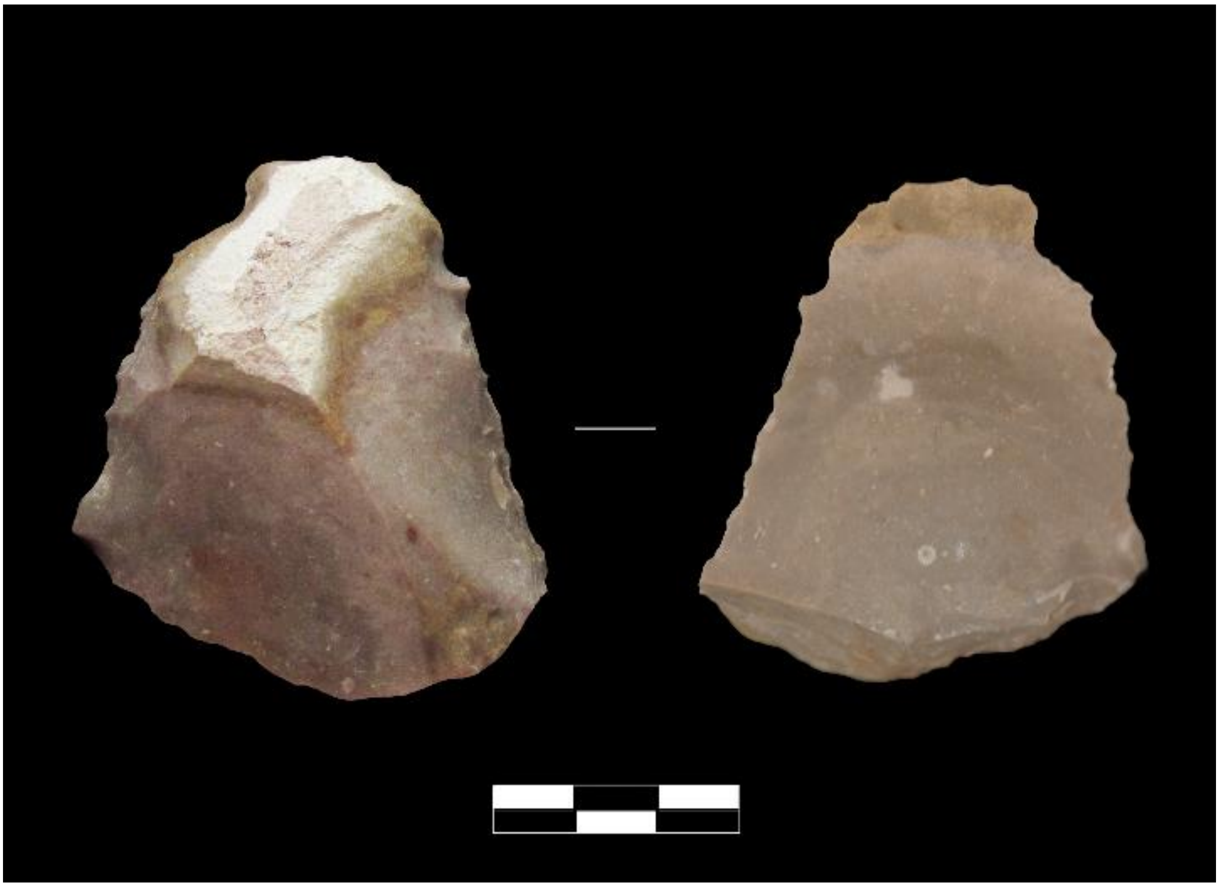

Retoucher ID: R6 **Knapper:** Y.L.P.

Use area #1 **N. of blows:** 28

Notes: long, thin flakes were removed, using the two areas complementary.

Use area #2 **N. of blows**: 61

Notes: long, thin flakes were removed, using the two areas complementary.

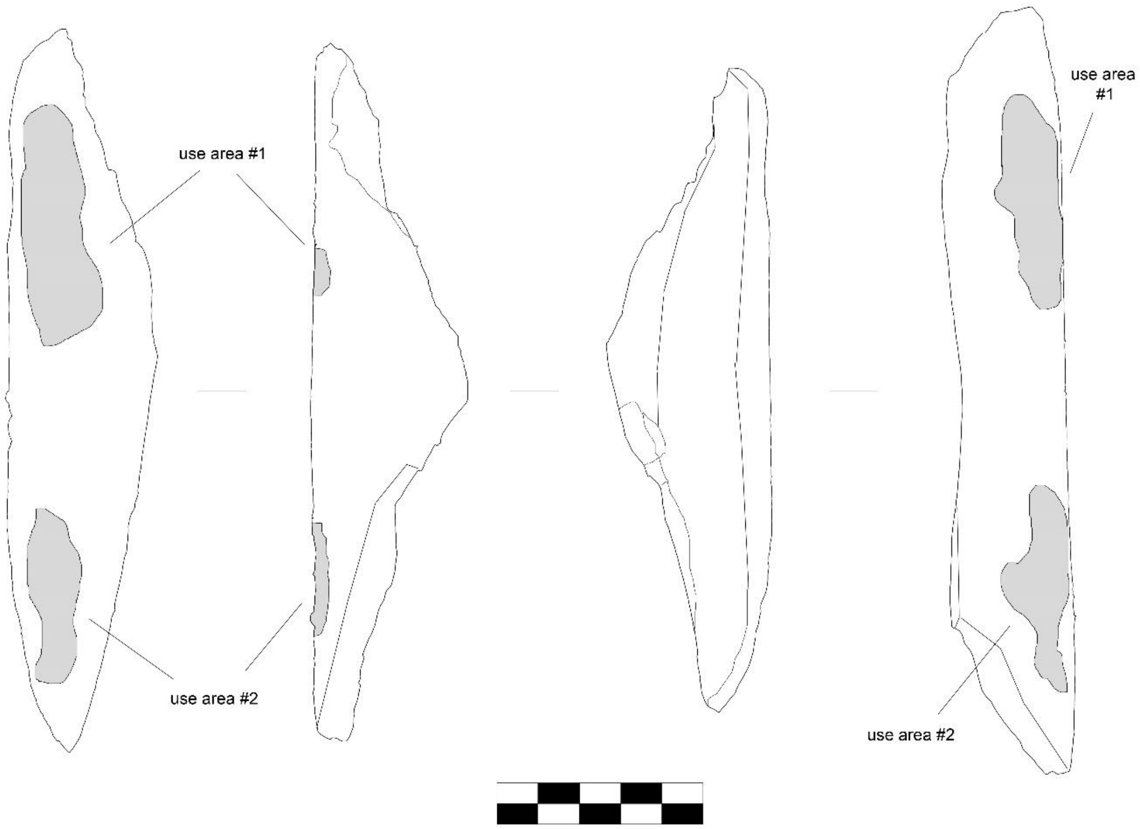

Flake code: F074

Description of the retouch:

Position: ☒direct ☐inverse ☐other

Delineation: ☐rectilinear ☒convex ☐concave ☐denticulated ☐other

Morphology: ☐scaled ☒stepped ☐parallel ☐sub-parallel

Localisation: ☒distal ☐mesial ☐proximal ☒right ☒left ☐basal

Distribution: ☒continuous ☐discontinuous ☐partial

Extent: ☒short ☐long ☐invasive

Angle: ☒abrupt (~90°) ☒semi-abrupt (~45°) ☐low (<30°)

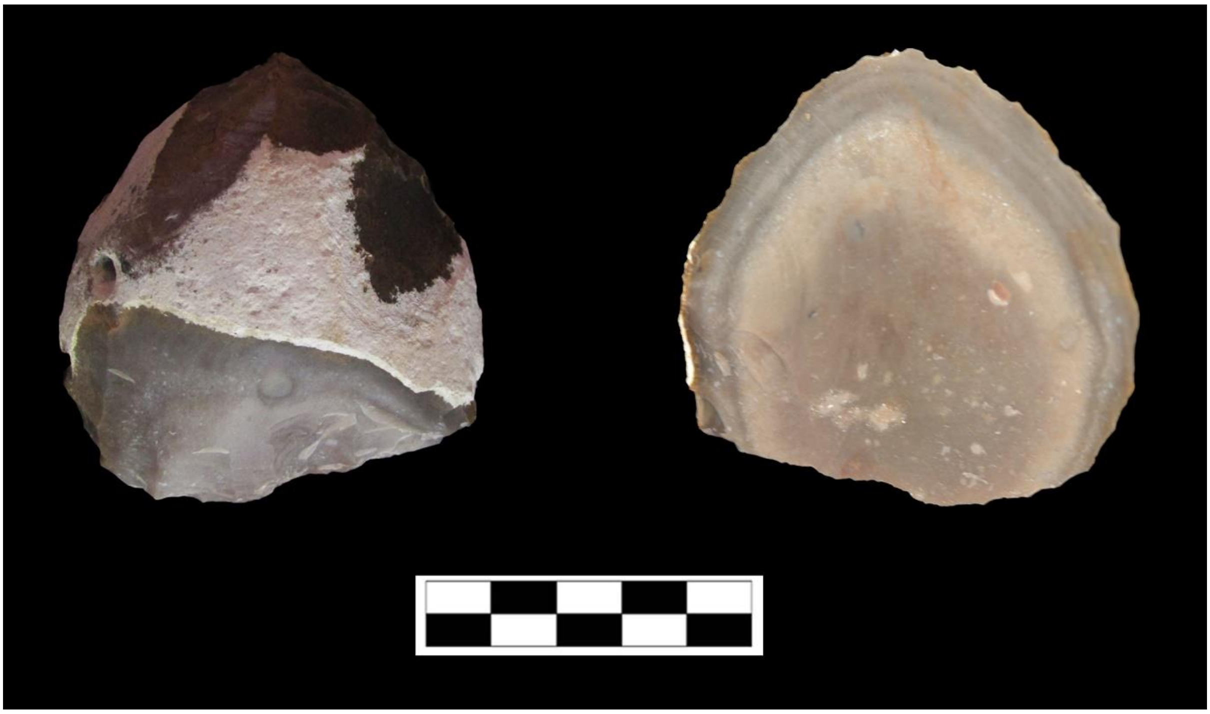

Retoucher ID: R7

Knapper: T.R.M.

Use area #1

N. of blows: 7

Notes: ‘bifacial’ technique of retouching. Two small bone fragments detach from the apical portion of the retoucher. The operator decides of looking for a better portion of the bone tool.

Use area #2

N. of blows: 53

Notes: this area seems to work better; the operator feels that a more pronounced convexity of the surface improves the retouch. The operator often abrades the lithic edge with the edge of the bone retoucher.

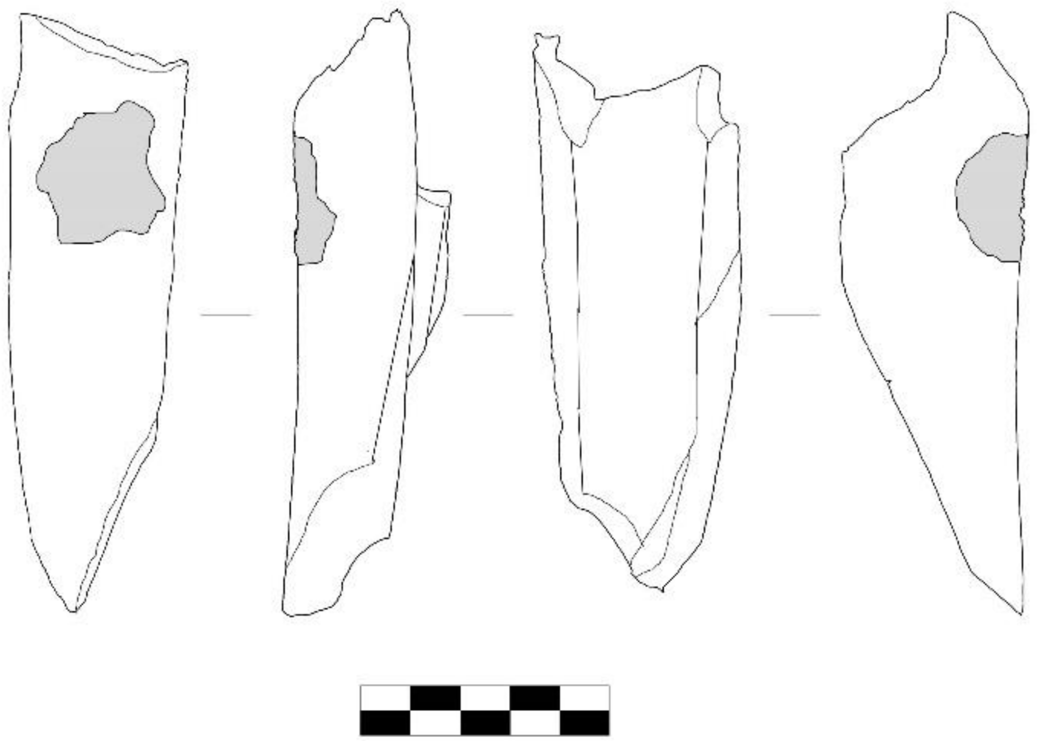

Flake code: F37

Description of the retouch:

Position: ☒direct ☐inverse ☐other

Delineation: ☒rectilinear ☒convex ☐concave ☐denticulated ☐other

Morphology: ☒scaled ☒stepped ☐parallel ☐sub-parallel

Localisation: ☐distal ☐mesial ☐proximal ☒right ☒left ☐basal

Distribution: ☒continuous ☐discontinuous ☒partial

Extent: ☒short ☐long ☐invasive

Angle: ☒abrupt (~90°) ☒semi-abrupt (~45°) ☐low (<30°)

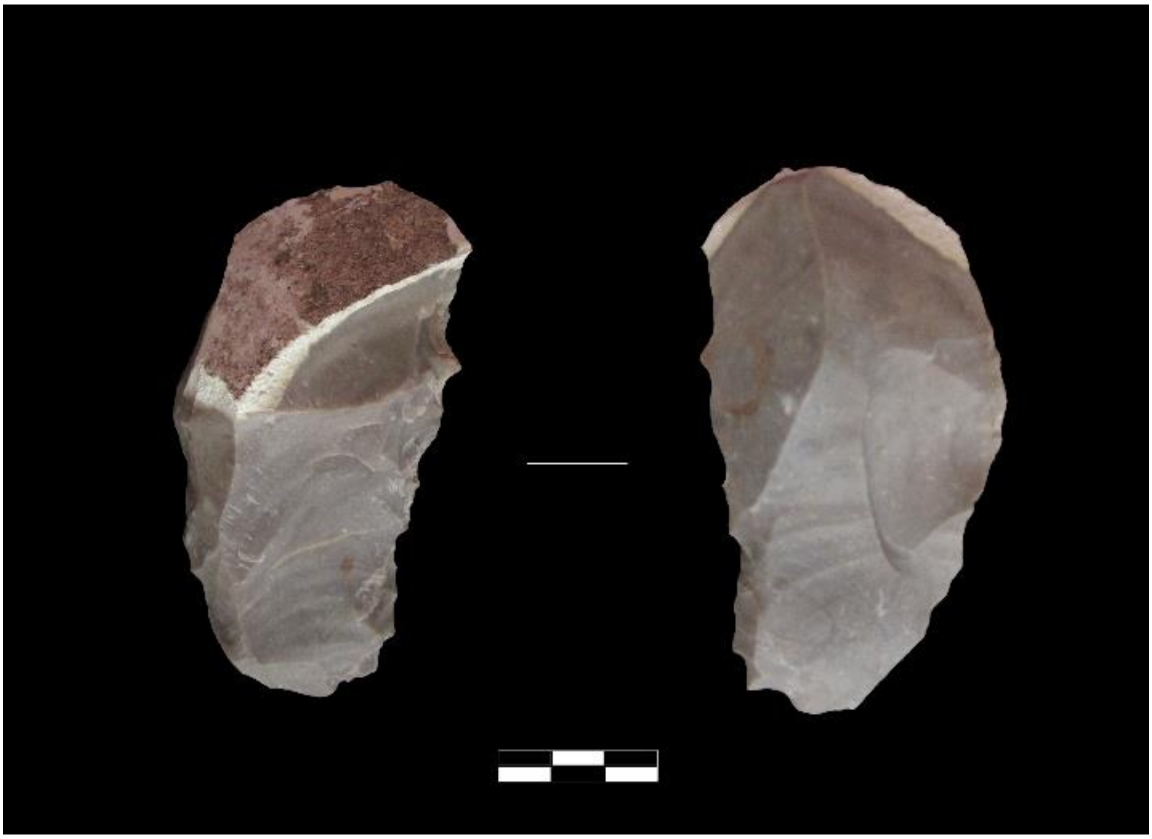

Retoucher ID: R8 **Knapper:** Y.L.P.

Use area #1 **N. of blows:** 189

Notes: This is the most exploited area. the elliptical trajectory is emphasised by the operator while using this area of the tool. The movement is sometimes interrupted to regularise the lithic edge by means of gentle taps against the bone surface.

Use area #2 **N. of blows**: 9

Notes: The operator tested this portion of the tool but it was discarded after few blows because considered less comfortable than use area #1

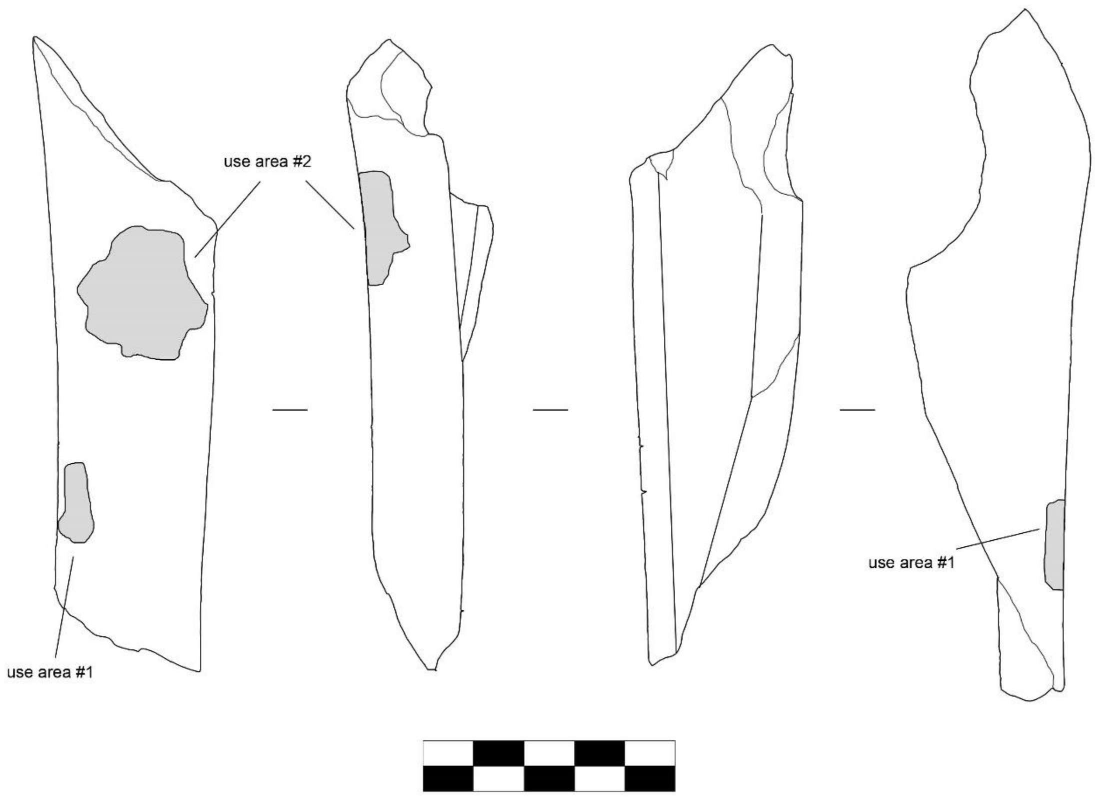

Flake code: F50

Description of the retouch:

Position: ☒direct ☐inverse ☐other

Delineation: ☒rectilinear ☒convex ☐concave ☐denticulated ☐other

Morphology: ☒scaled ☐stepped ☒parallel ☒sub-parallel

Localisation: ☒distal ☐mesial ☐proximal ☒right ☒left ☐basal

Distribution: ☒continuous ☐discontinuous ☐partial

Extent: ☐short ☒long ☐invasive

Angle: ☒abrupt (~90°) ☒semi-abrupt (~45°) ☐low (<30°)

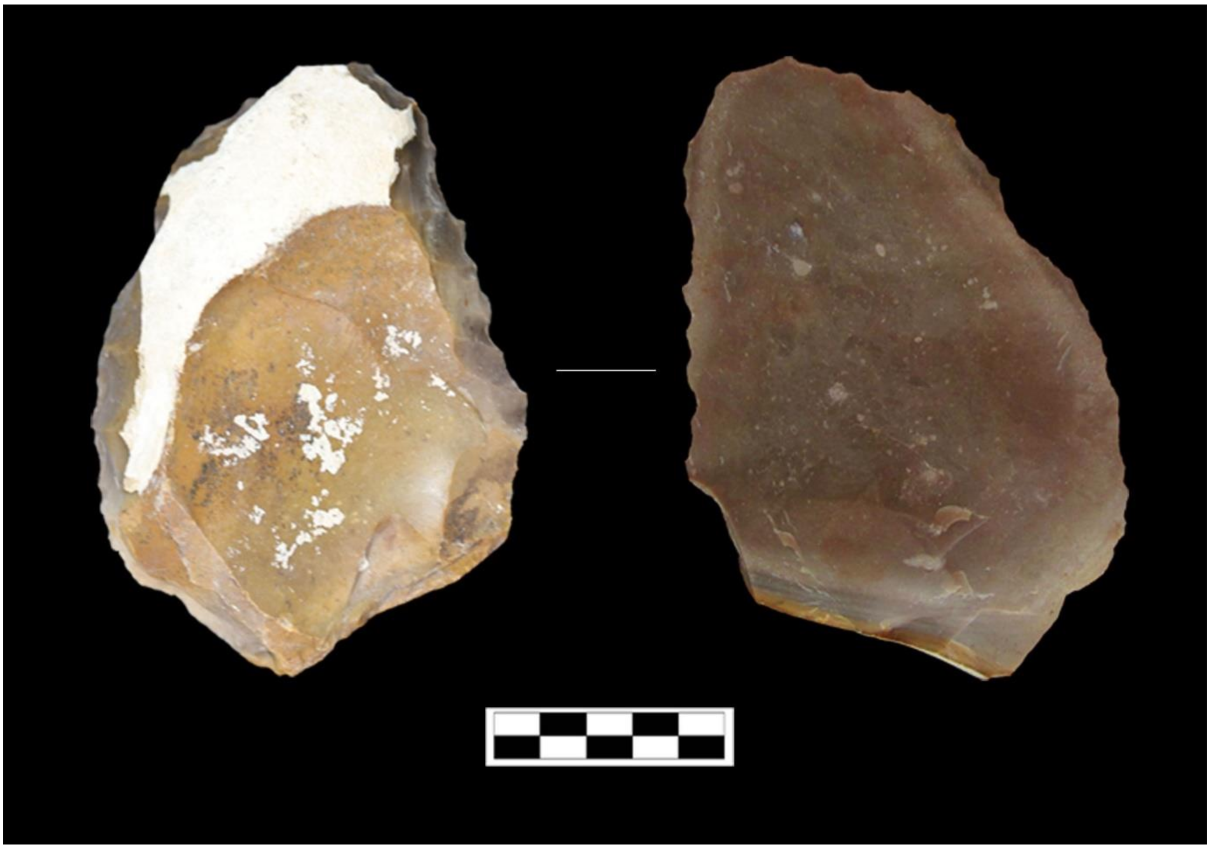

Retoucher ID: R9

Knapper: T.R.M.

Use area #1

N. of blows: 59

Notes: at the beginning of the retouch, the operator is aiming at an unifacial point-shape. During the retouch, the operator abrades the lithic edge a lot: differently from Y.P., which uses a ‘vertical tapping’, T.M. has a different movement of abrasion, more horizontal/transversal.

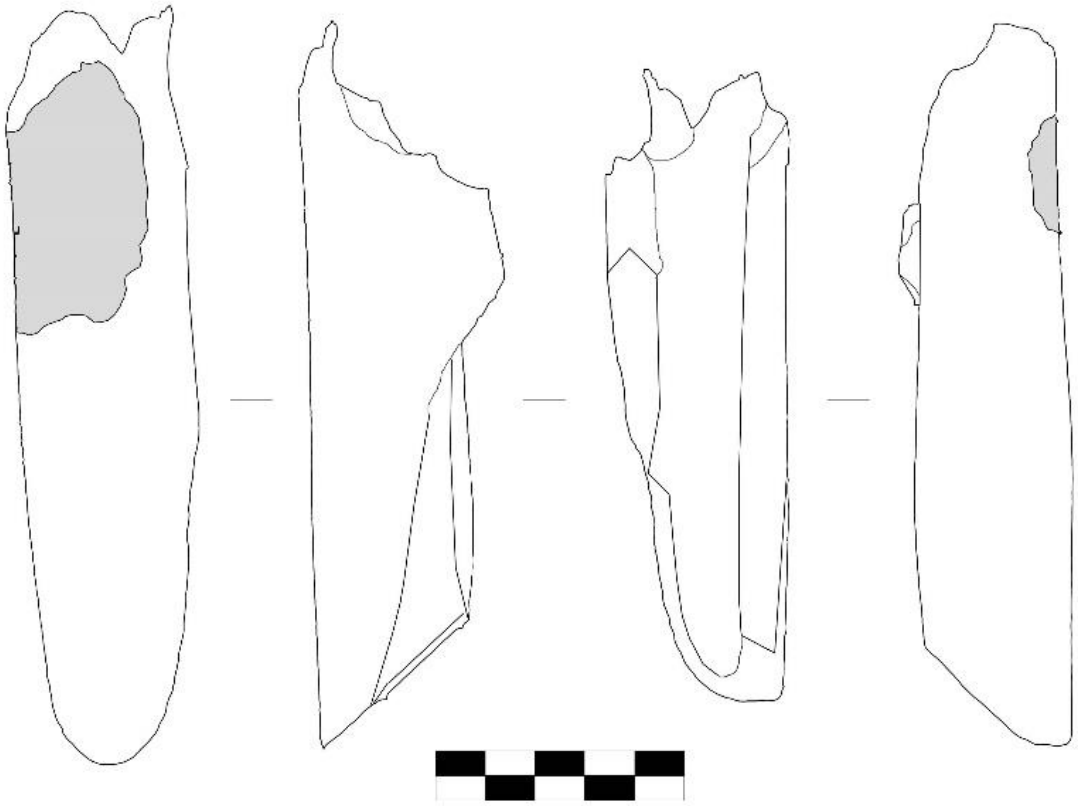

Flake code: F55

Description of the retouch:

Position: ☒direct ☐inverse ☐other

Delineation: ☐rectilinear ☐convex ☐concave ☒denticulated ☐other

Morphology: ☐scaled ☒stepped ☐parallel ☐sub-parallel

Localisation: ☒distal ☐mesial ☐proximal ☒right ☒left ☐basal

Distribution: ☒continuous ☐discontinuous ☐partial

Extent: ☐short ☐long ☒invasive

Angle: ☒abrupt (~90°) ☒semi-abrupt (~45°) ☐low (<30°)

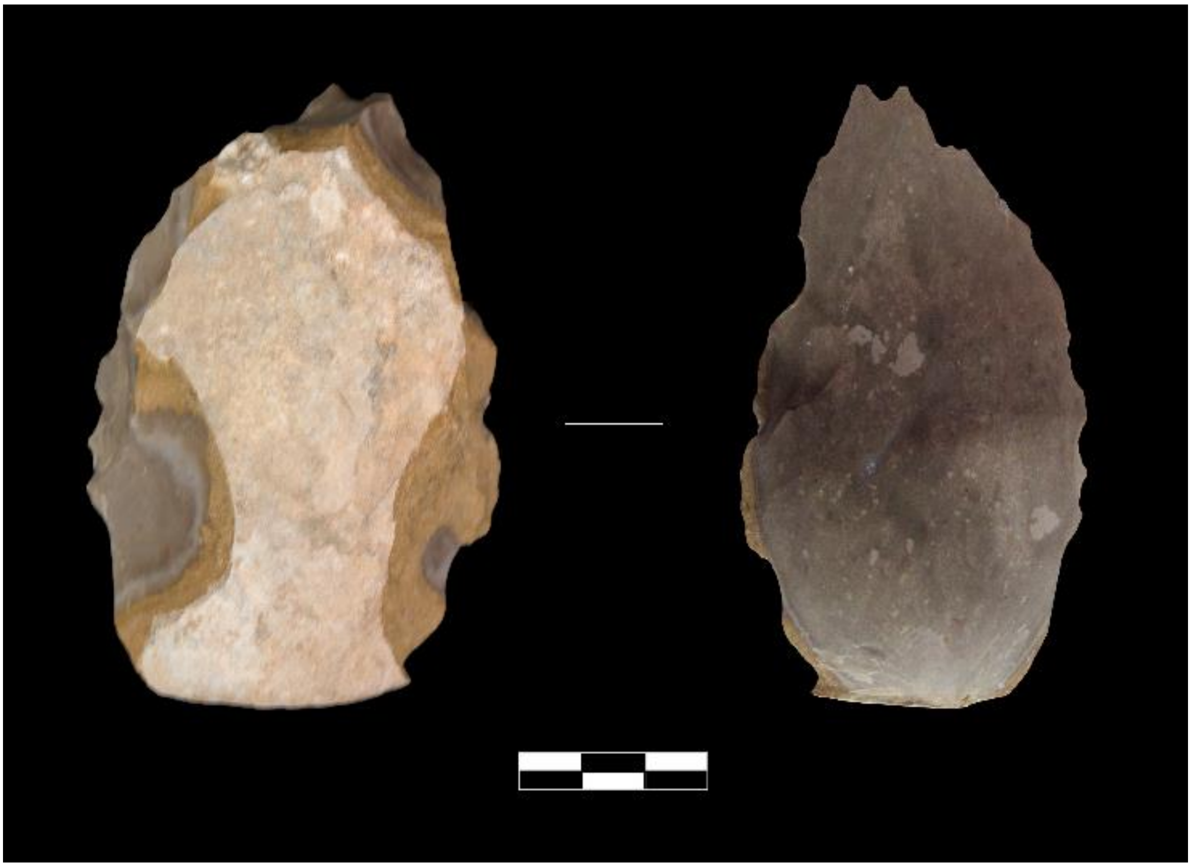

Retoucher ID: R10

Knapper: T.R.M.

Use area #1

N. of blows: 4

Notes: several abrasion motions. This area has been used only for few blows, then the operator looked for another portion of the bone.

Use area #2

N. of blows: 46

Notes: this area seems to be better, and it has been used until the completion of retouch.

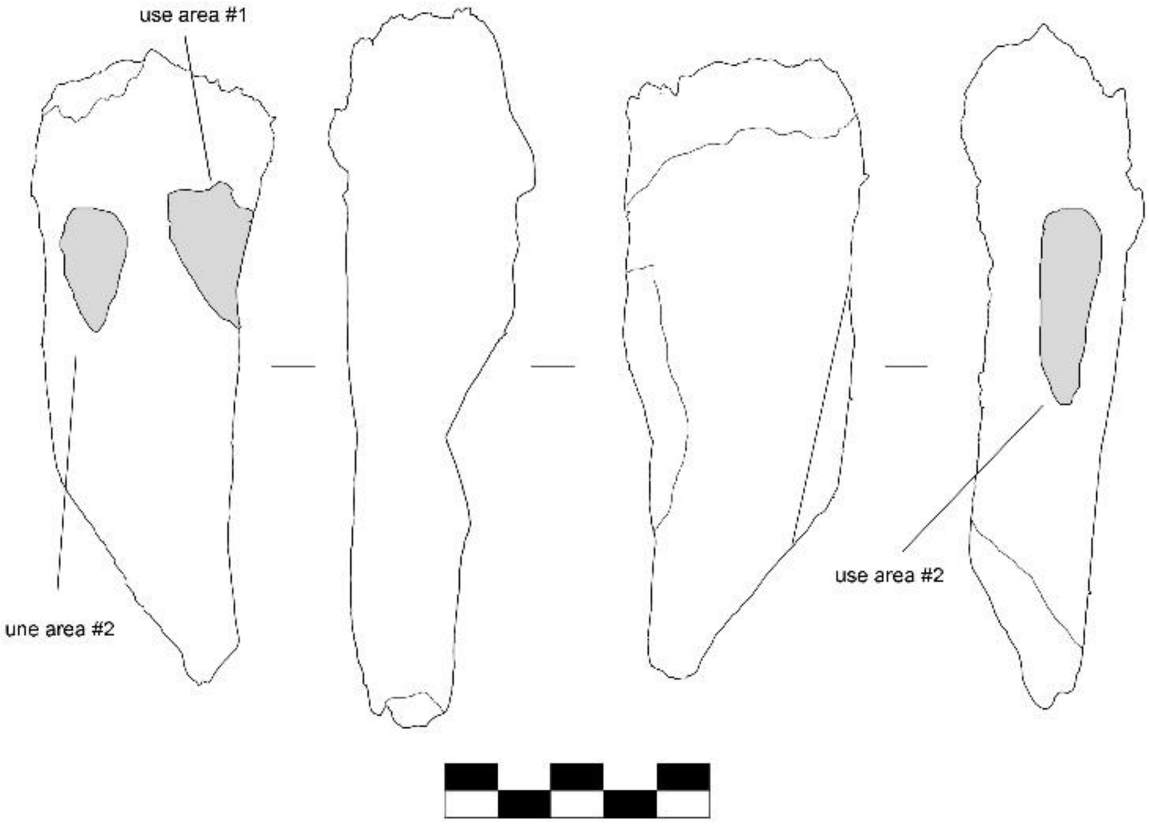

Flake code: F78

Description of the retouch:

Position: ☒direct ☐inverse ☐other

Delineation: ☐rectilinear ☐convex ☐concave ☒denticulated ☐other

Morphology: ☒scaled ☐stepped ☐parallel ☐sub-parallel

Localisation: ☒distal ☐mesial ☐proximal ☒right ☐left ☐basal

Distribution: ☒continuous ☐discontinuous ☐partial

Extent: ☐short ☒long ☐invasive

Angle: ☐abrupt (~90°) ☐semi-abrupt (~45°) ☒low (<30°)

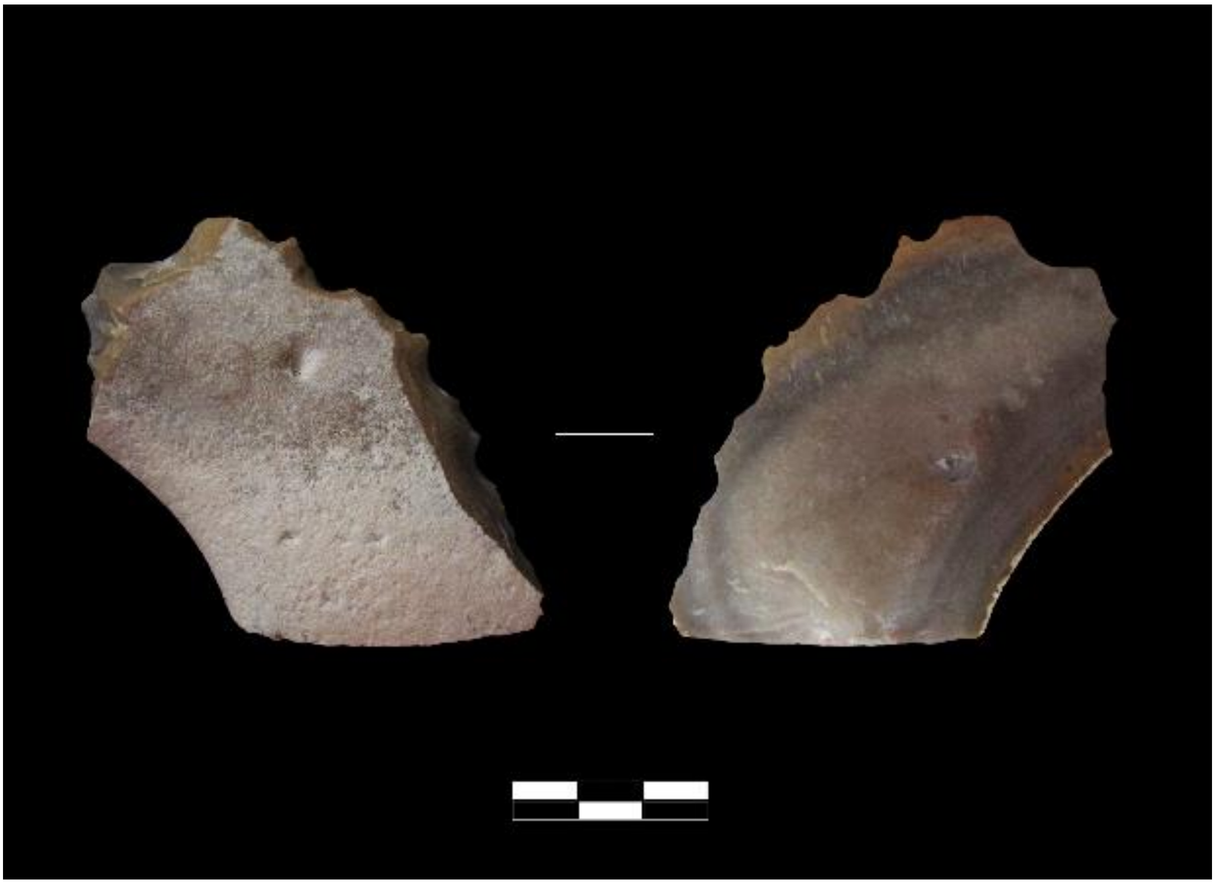

Retoucher ID: R11 **Knapper:** T.R.M.

Use area #1 **N. of blows:** 12

Notes: detachment of the apical portion of the retoucher. The operator abandoned this area because it was difficult to avoid the apical portion.

Use area #2 **N. of blows:** 25

Notes: during the percussion using this area, a portion of bone from the opposite end of the tool detached. After few blows, another apical portion (on the side of this use area) detached, so the operator abandoned this area.

Use area #3 **N. of blows: 31**

Notes: this area seems to be better, and it has been used until completion of the retouch.

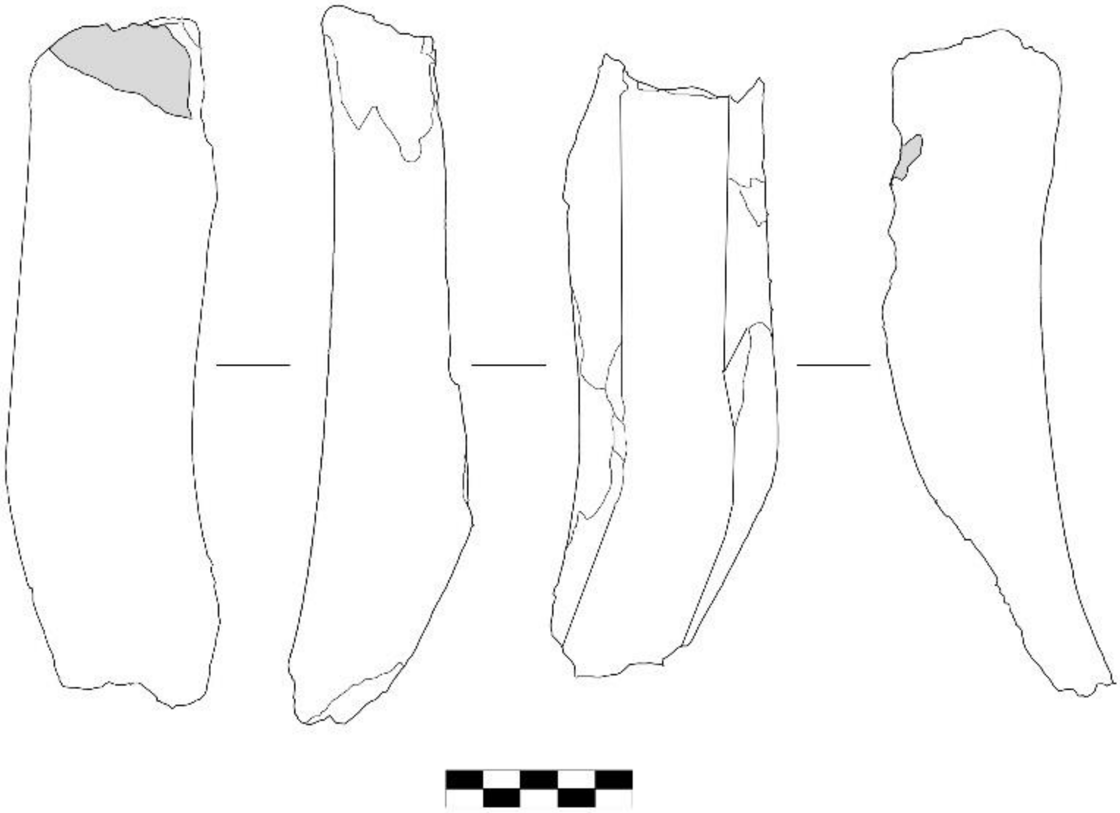

Flake code: F54

Description of the retouch:

Position: ☐direct ☐inverse ☒other (bifacial)

Delineation: ☐rectilinear ☐convex ☐concave ☐denticulated ☒other (irregular)

Morphology: ☒scaled ☐stepped ☒parallel ☐sub-parallel

Localisation: ☒distal ☐mesial ☐proximal ☒right ☐left ☐basal

Distribution: ☒continuous ☐discontinuous ☐partial

Extent: ☒short ☐long ☒invasive

Angle: ☐abrupt (~90°) ☐semi-abrupt (~45°) ☒low (<30°)

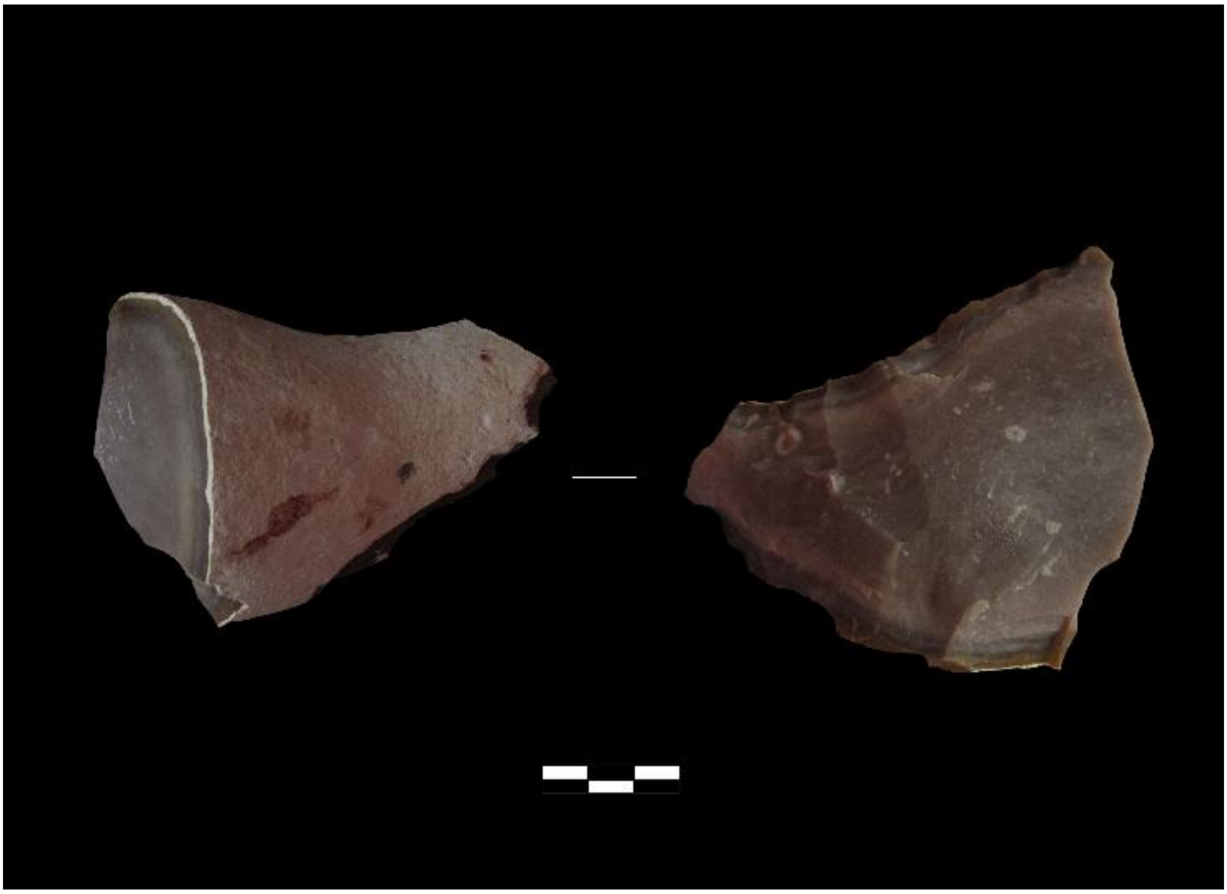

Retoucher ID: R12

Use area #1

Notes: none

Knapper: Y.L.P.

N. of blows: 50

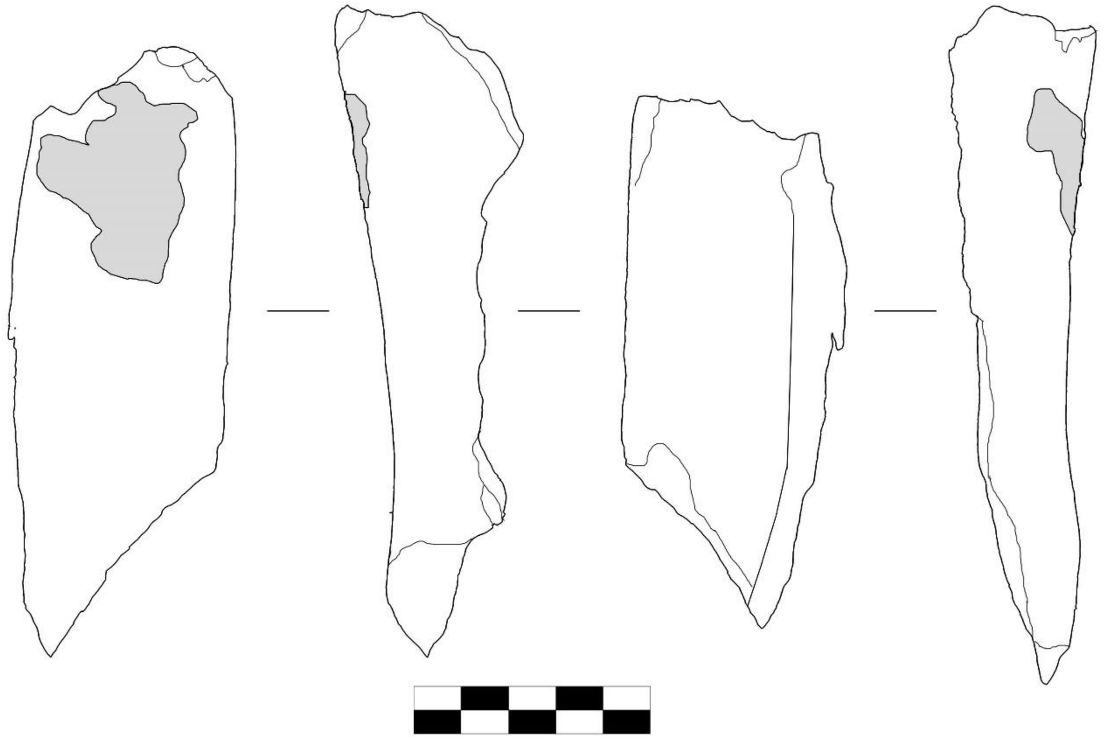

Flake code: F47a

Description of the retouch:

Position: ☒direct ☐inverse ☐other

Delineation: ☒rectilinear ☒convex ☐concave ☐denticulated ☐other

Morphology: ☒scaled ☐stepped ☒parallel ☐sub-parallel

Localisation: ☒distal ☐mesial ☐proximal ☒right ☐left ☐basal

Distribution: ☐continuous ☐discontinuous ☐partial

Extent: ☒short ☐long ☐invasive

Angle: ☐abrupt (~90°) ☐semi-abrupt (~45°) ☒low (<30°)

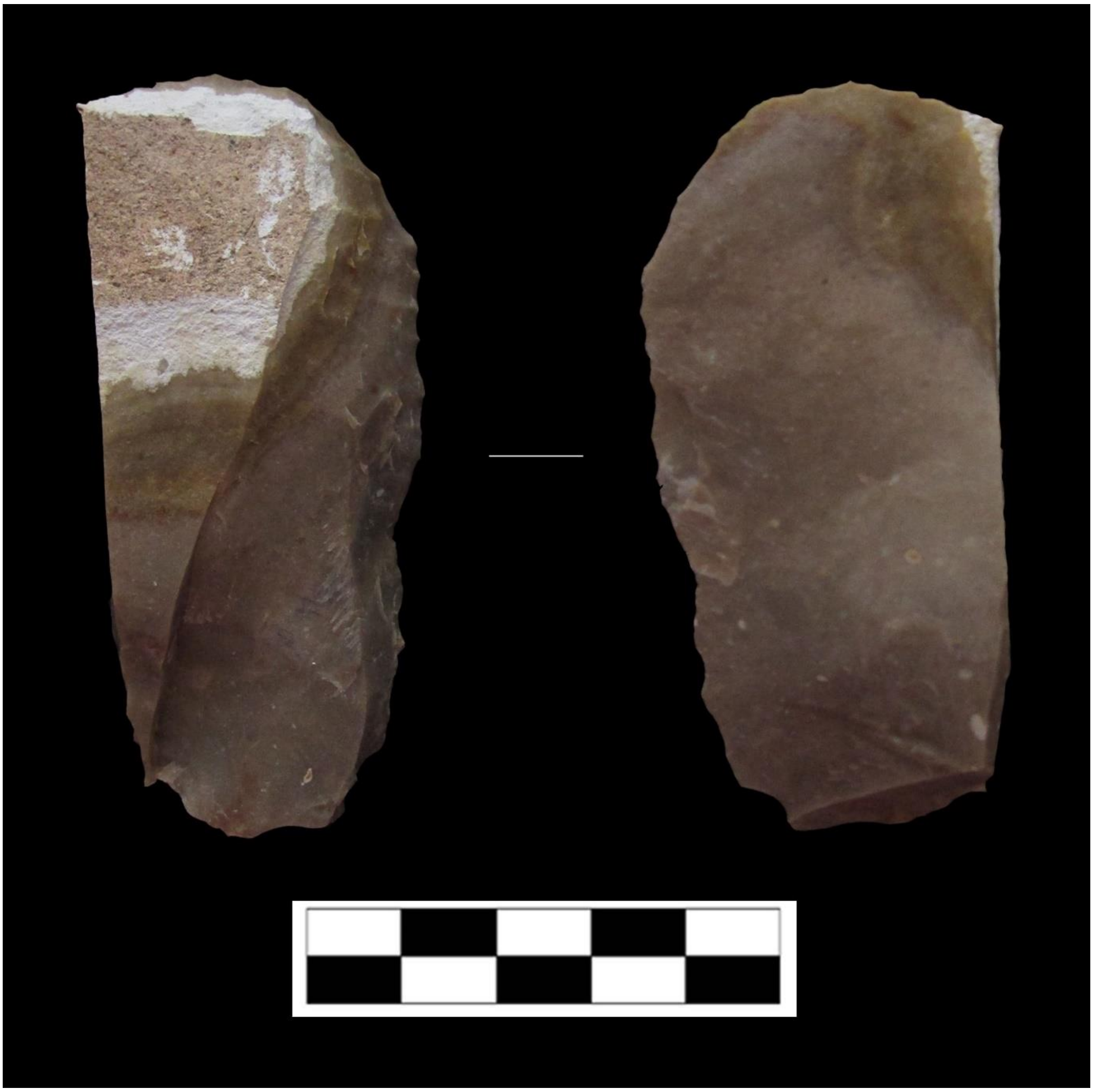

Retoucher ID: R15 **Knapper:** Y.L.P.

Use area #1 **N. of blows:** 27

Notes: the area has been abandoned because the surface is too concave

Use area #2 **N. of blows:** 76

Notes: this area seems to be better, although the operator complaints that the retoucher is uncomfortable because too small. After a while the retoucher seems to work better than expected (operator's opinion). The movement tends to 'brushing' – maybe because of the size.

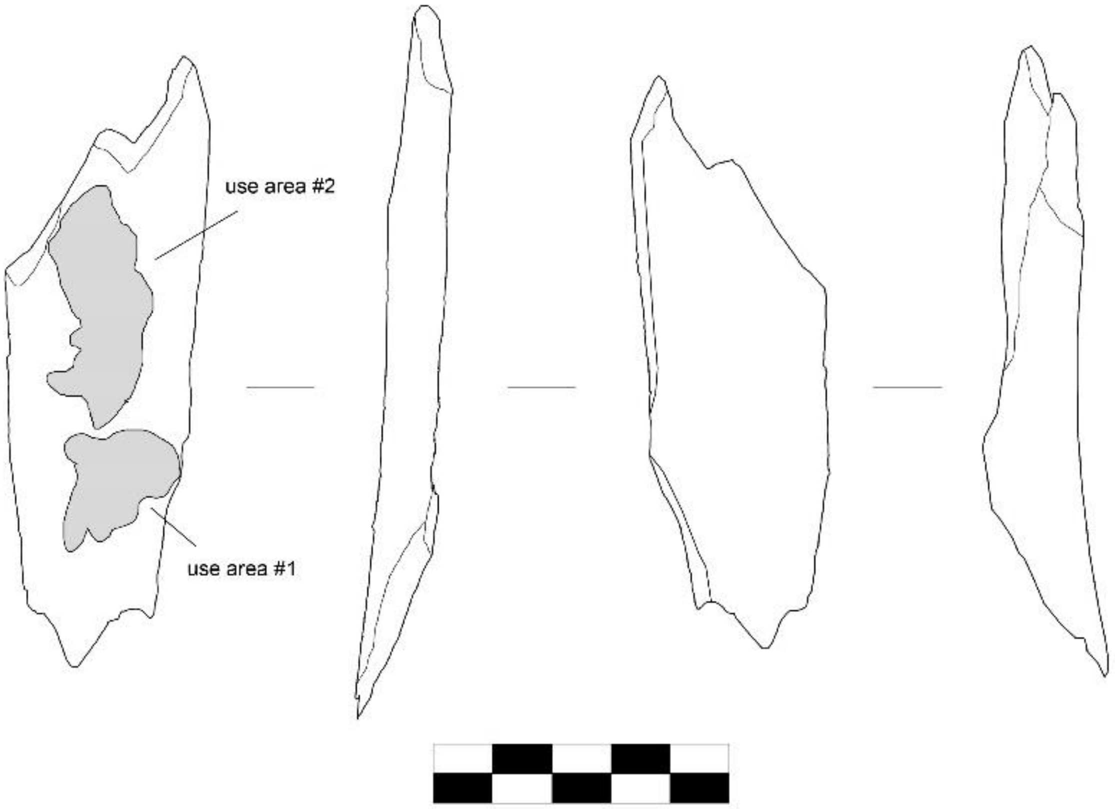

Flake code: F06

Description of the retouch:

Position: ☒direct ☐inverse ☐other

Delineation: ☒rectilinear ☒convex ☐concave ☐denticulated ☐other

Morphology: ☐scaled ☐stepped ☐parallel ☒sub-parallel

Localisation: ☒distal ☐mesial ☐proximal ☒right ☒left ☐basal

Distribution: ☒continuous ☐discontinuous ☐partial

Extent: ☒short ☐long ☐invasive

Angle: ☐abrupt (~90°) ☒semi-abrupt (~45°) ☐low (<30°)

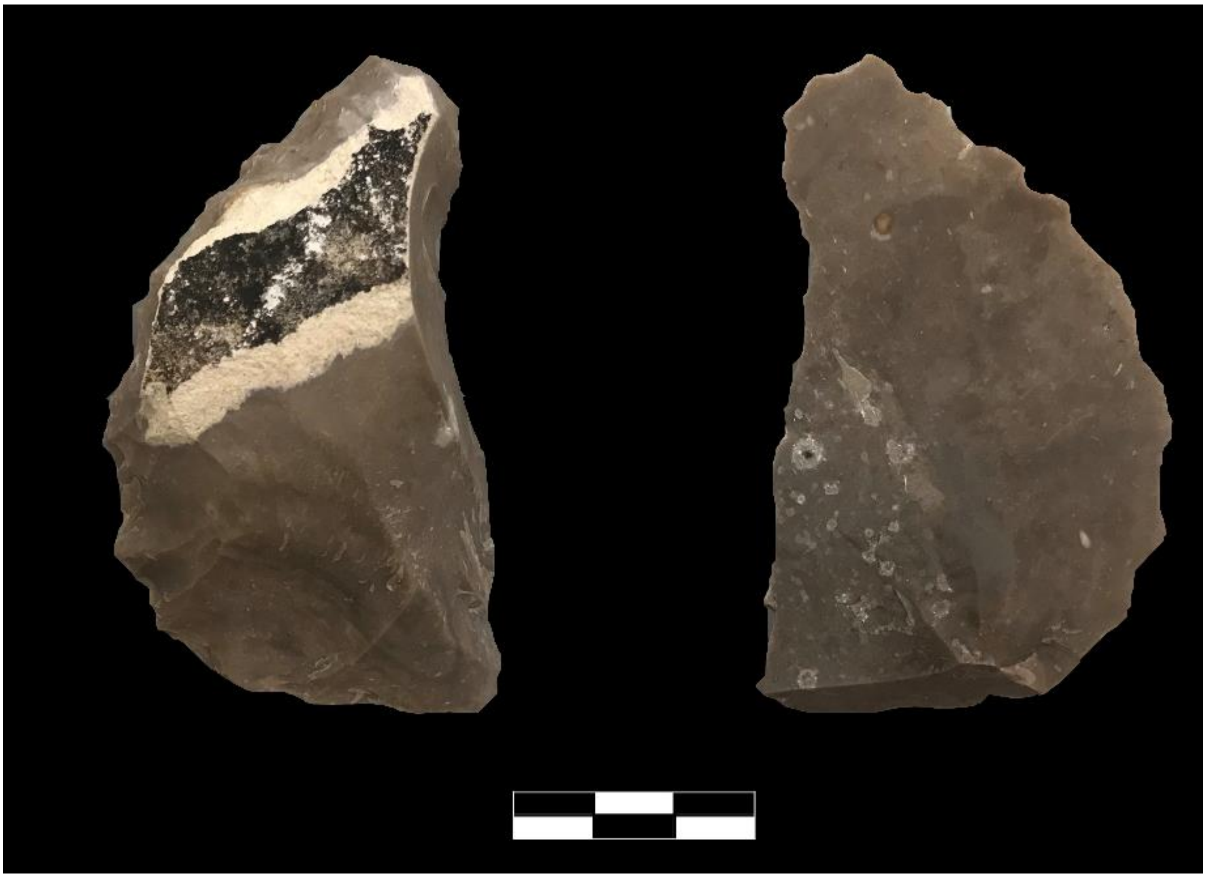

Retoucher ID: R17

Knapper: T.R.M.

Use area #1

N. of blows: 63

Notes: this is the first retoucher, and the operator is still learning the technique. The operator hits using the edge.

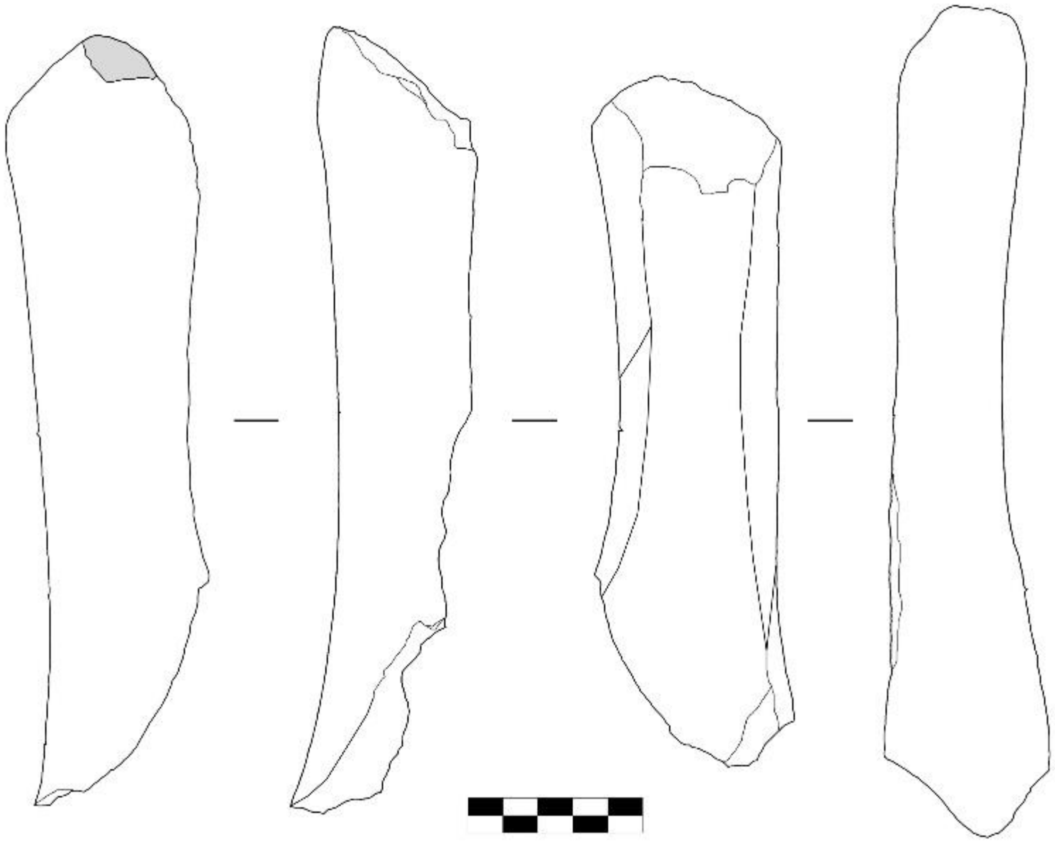

Flake code: F05

Description of the retouch:

Position: ☒direct ☐inverse ☐other

Delineation: ☐rectilinear ☐convex ☐concave ☒denticulated ☐other

Morphology: ☒scaled ☐stepped ☐parallel ☐sub-parallel

Localisation: ☒distal ☐mesial ☐proximal ☒right ☒left ☐basal

Distribution: ☒continuous ☐discontinuous ☐partial

Extent: ☒short ☐long ☐invasive

Angle: ☐abrupt (~90°) ☒semi-abrupt (~45°) ☐low (<30°)

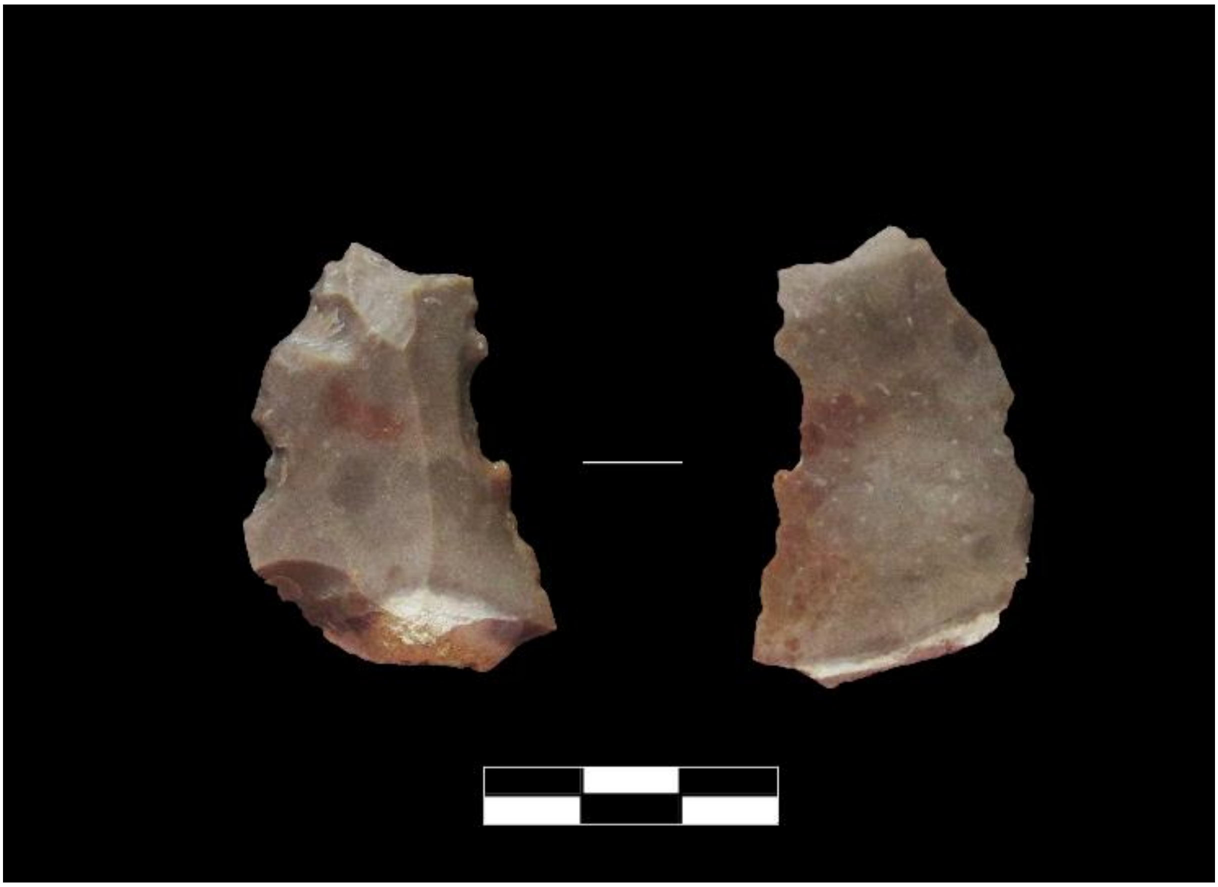

Retoucher ID: R18

Knapper: T.R.M.

Use area #1

N. of blows: 9

Notes: a big fragment of the lithic flake detaches – the flake is abandoned.

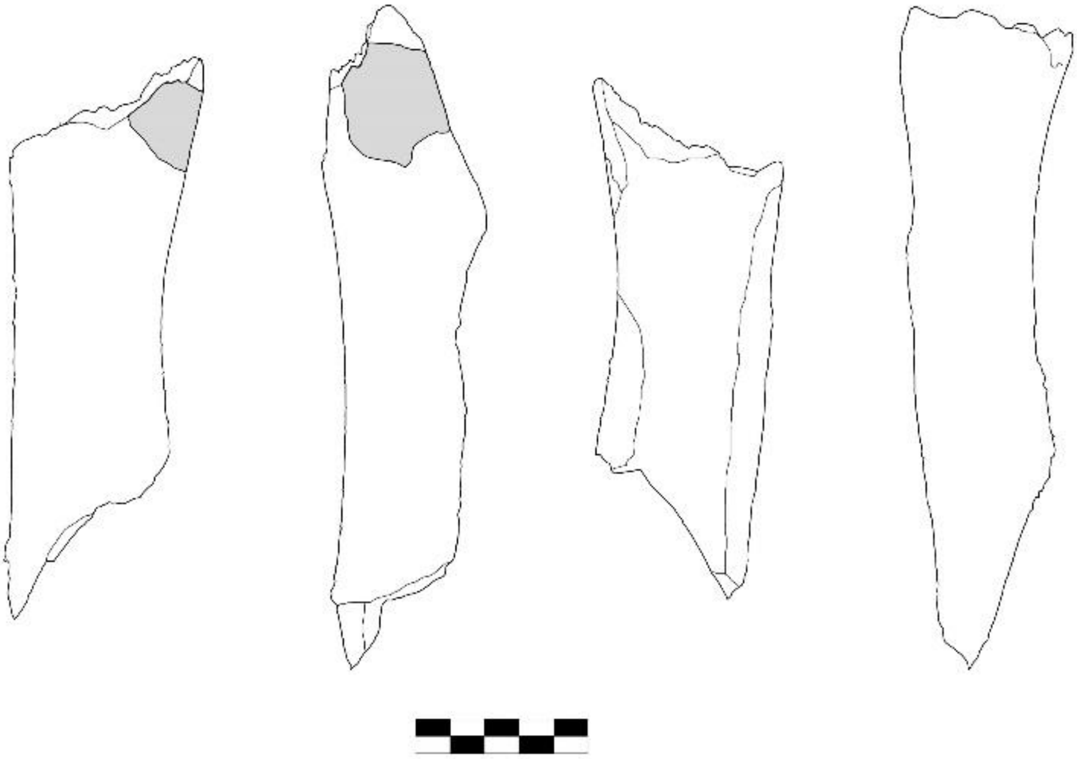

Flake code: F25

Description of the retouch:

Position: ☐direct ☒inverse ☐other

Delineation: ☒rectilinear ☐convex ☐concave ☐denticulated ☐other

Morphology: ☒scaled ☐stepped ☐parallel ☐sub-parallel

Localisation: ☐distal ☒mesial ☐proximal ☐right ☒left ☐basal

Distribution: ☐continuous ☐discontinuous ☒partial

Extent: ☒short ☐long ☐invasive

Angle: ☐abrupt (~90°) ☒semi-abrupt (~45°) ☐low (<30°)

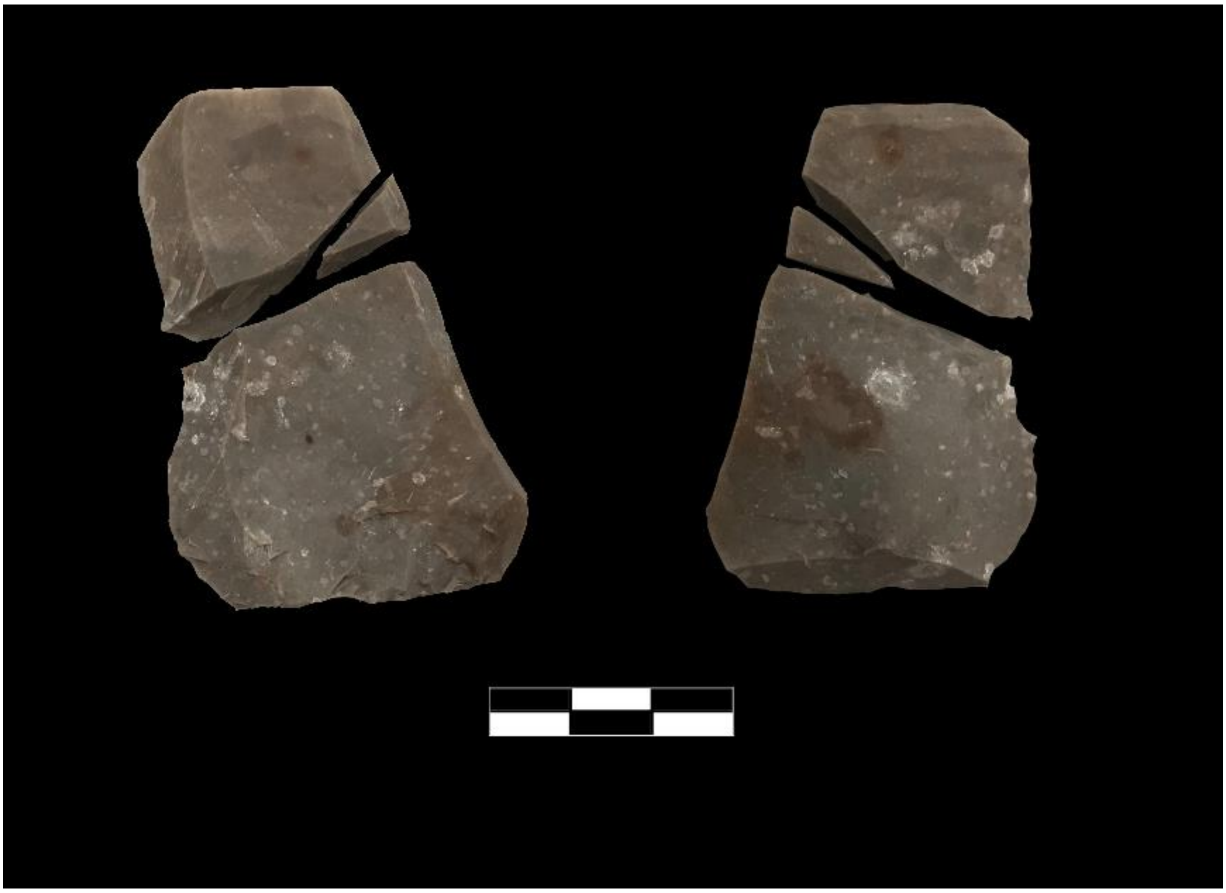

Retoucher ID: R19

Knapper: T.R.M.

Use area #1

N. of blows: 28

Notes: uncertain movements. A lot of abrasion.

Use area #2

N. of blows: 25

Notes: this area seems to work better, but some bone flakes are still detaching from the apical portion.

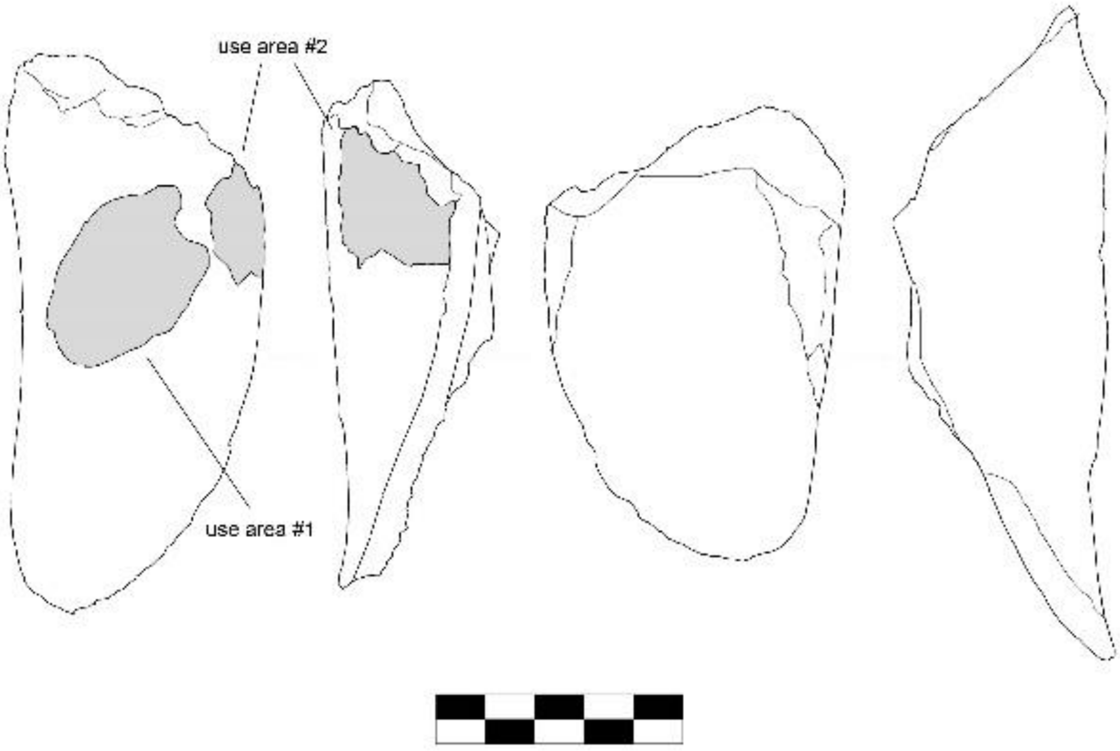

Flake code: F58

Description of the retouch:

Position: ☒direct ☐inverse ☐other

Delineation: ☐rectilinear ☐convex ☐concave ☒denticulated ☐other

Morphology: ☐scaled ☒stepped ☐parallel ☒sub-parallel

Localisation: ☒distal ☐mesial ☐proximal ☒right ☐left ☐basal

Distribution: ☒continuous ☐discontinuous ☒partial

Extent: ☒short ☒long ☐invasive

Angle: ☐abrupt (~90°) ☒semi-abrupt (~45°) ☐low (<30°)

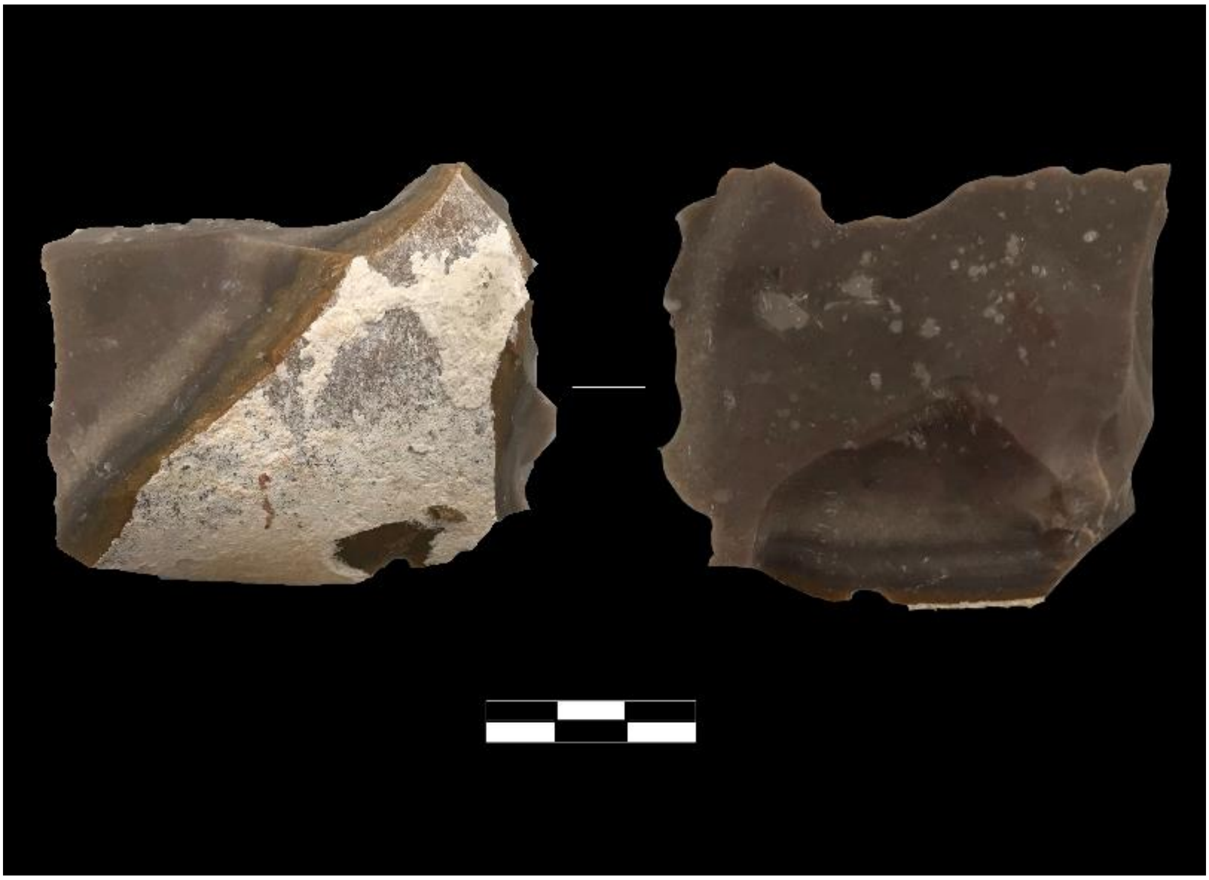

Retoucher ID: R20 **Knapper**: Y.L.P.

Use area #1 **N. of blows:** 100

Notes: the operator is aiming for larger retouch flakes. Several bending initiations on detached retouch-flakes (bulbs pronounced) – especially on larger ones. the operator describes the retoucher as 'a dense bone with very pronounced convex area, making it fairly easy to get a good blow'.

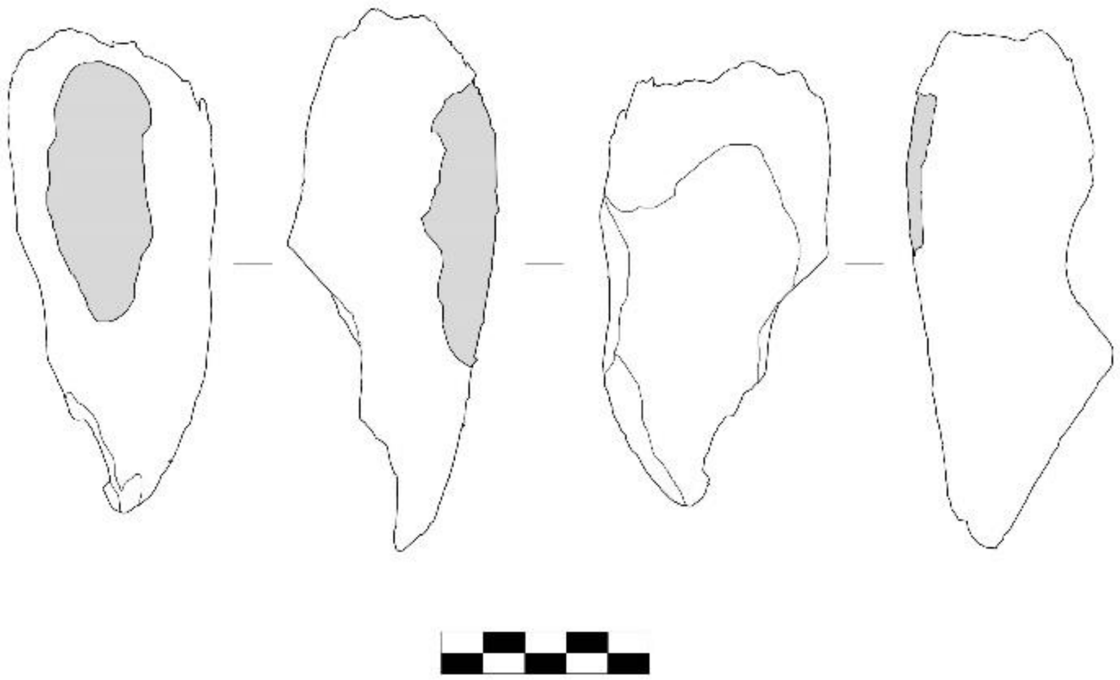

Flake code: F30

Description of the retouch:

Position: ☒direct ☐inverse ☐other

Delineation: ☐rectilinear ☐convex ☐concave ☐denticulated ☒other (irregular)

Morphology: ☐scaled ☒stepped ☒parallel ☐sub-parallel

Localisation: ☐distal ☐mesial ☐proximal ☒right ☒left ☐basal

Distribution: ☒continuous ☐discontinuous ☐partial

Extent: ☒short ☐long ☐invasive

Angle: ☐abrupt (~90°) ☒semi-abrupt (~45°) ☐low (<30°)

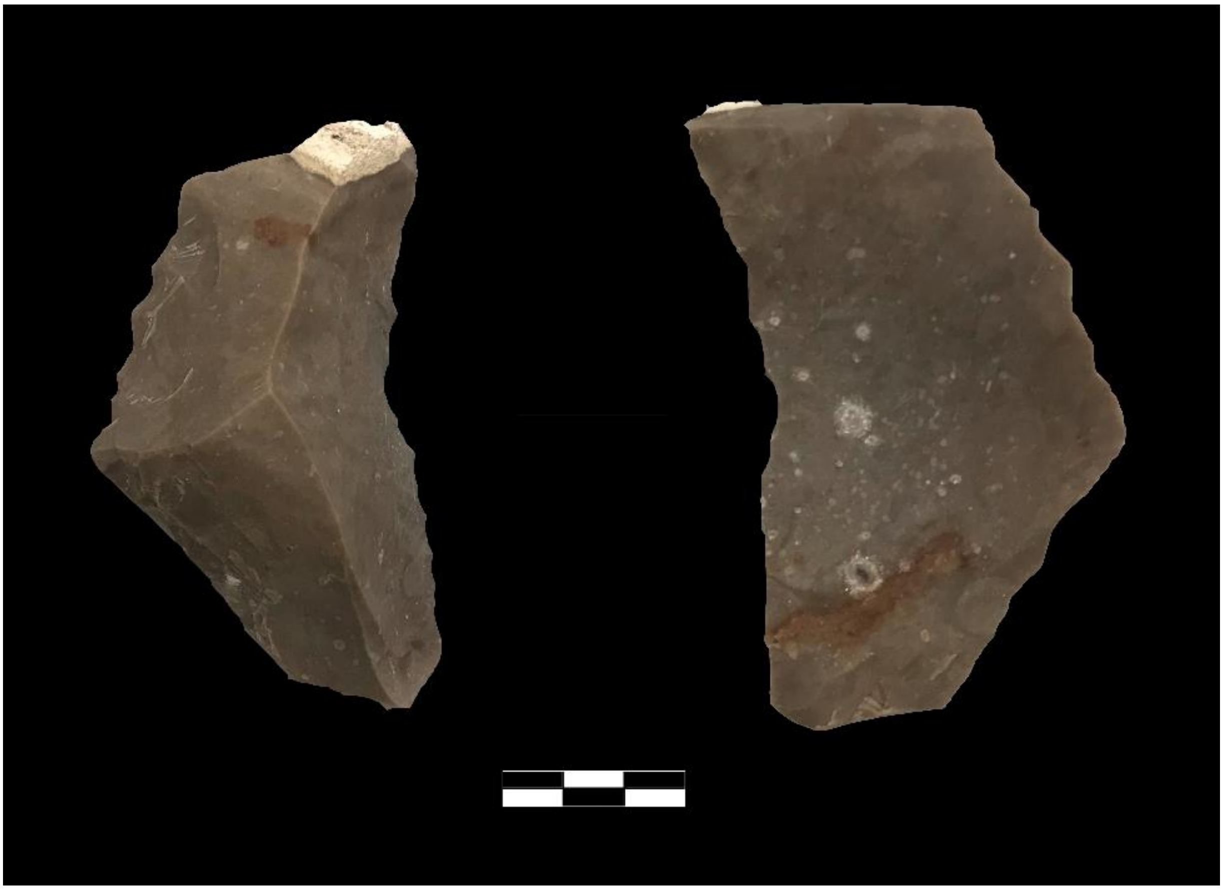

Retoucher ID: R21

Knapper: Y.L.P.

Use area #1

N. of blows: 118

Notes: this retoucher is highly efficient. The operator used an elliptical movement with a tangential contact, and they applied several regularisations of the lithic edge against the osseous surface.

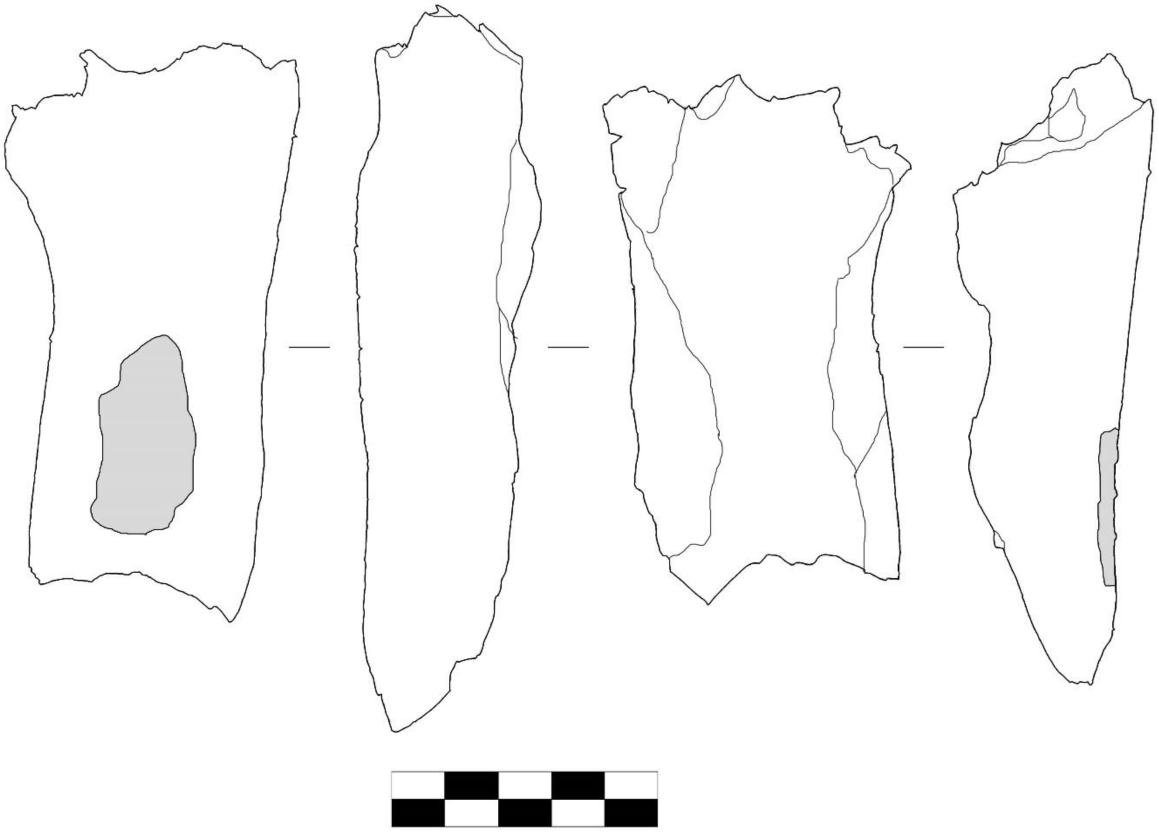

Flake code: F39

Description of the retouch:

Position: ☒direct ☐inverse ☐other

Delineation: ☒rectilinear ☐convex ☐concave ☐denticulated ☒other (irregular)

Morphology: ☐scaled ☒stepped ☒parallel ☐sub-parallel

Localisation: ☒distal ☐mesial ☐proximal ☒right ☒left ☐basal

Distribution: ☒continuous ☐discontinuous ☒partial

Extent: ☐short ☒long ☐invasive

Angle: ☐abrupt (~90°) ☒semi-abrupt (~45°) ☐low (<30°)

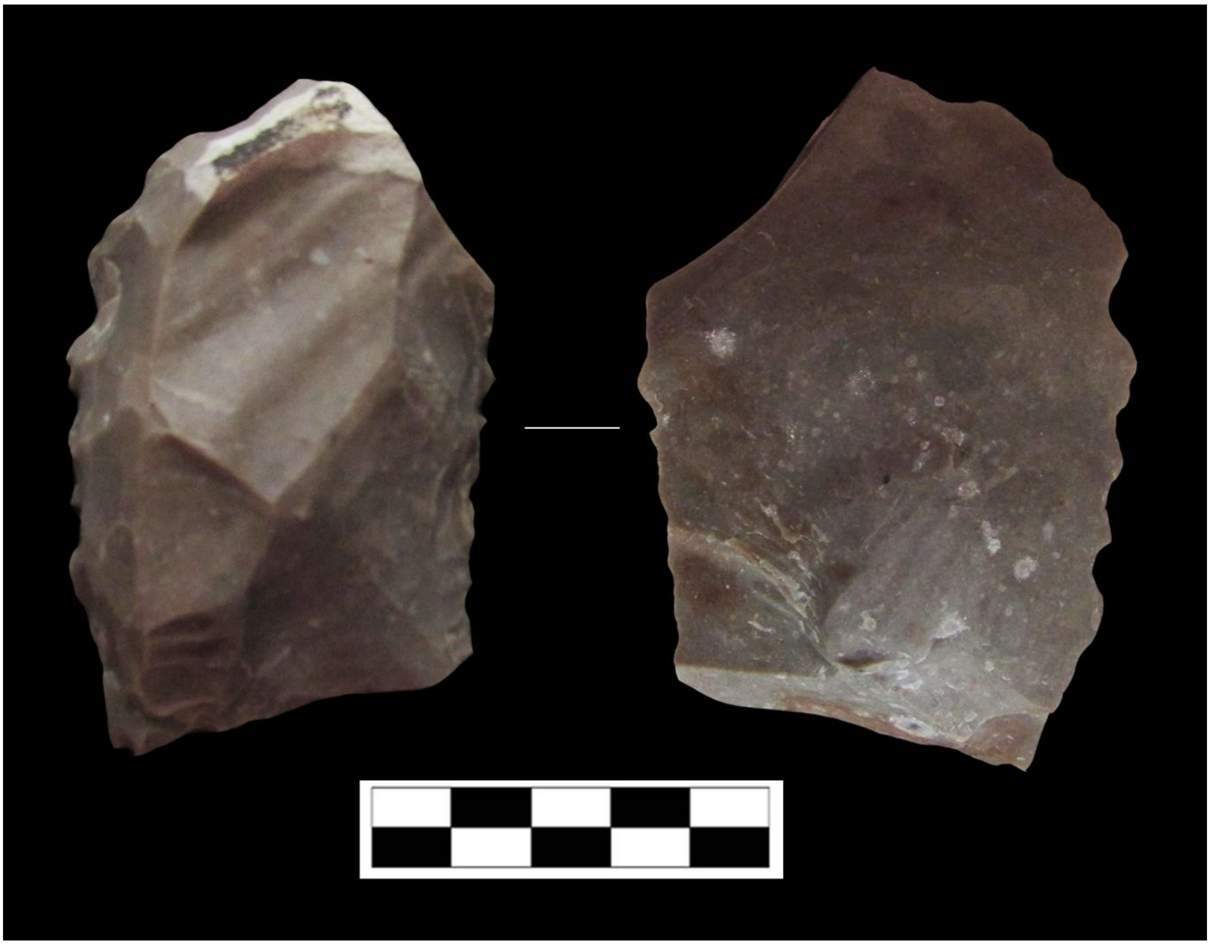

Supplement: S3 File — In each template, the first picture represents a 360° view of the bone retoucher and indication of the use area(s); the second picture is a photo of the retouched lithic flake. Retouchers described within the text (i.e., R6, R8, R12, R21) are excluded from this set (drawings and pictures by E. F. Martellotta). (PDF) [file pone.0273118.s011.pdf]
