# Supplementary material for: Beyond the main function: An experimental study of the use of hardwood boomerangs in retouching activities
Source: PLoS One. 2022 Aug 16;17(8):e0273118. doi: 10.1371/journal.pone.0273118 (PMC9380927; doi:10.1371/journal.pone.0273118)
Supplement: Attachment — Submitted filename: Review of PONE-D-22-05291 boomerang retouchers.pdf [file pone.0273118.s014.pdf]

Review of PONE-D-22-05291

This paper reports experiments in using boomerangs to retouch the edges of flakes, as described in various Australian ethnographic accounts. The paper compares the stigmata of retouching on wood boomerangs to the stigmata produced on bone retouching tools. The study addresses an interesting topic and has the potential to be an important contribution to the literature, but, as currently written, it suffers from a lack of a clear research question. This, in turn, has led to a disorganised manuscript that attempts to do too much. I will describe the two main issues I have with the paper to consider in revision, followed by detailed comments I wrote as I read through the paper.

One issue is that the purpose of the paper—the research question—is not clearly articulated. In the conclusion we learn that the experiment was apparently to test whether boomerangs 'can successfully function as retouchers'. But this is a non-question, as we already know from historical descriptions—reviewed in the senior author's cited publications—that boomerangs were certainly used successfully by Aboriginal people to retouch stone tools. This is not in itself an interesting or useful new observation, and an experimental approach adds nothing to it. Another purpose was to explore whether bone and wood indentors sustain similar stigmata when used to retouch tools. This is a novel and worthy research question, but the authors conclude at the outset of the study (e.g., Lines 224-225) that the stigmata categories are the same for bone and wood, so it is a logical fallacy to nominate this as an experimental goal (Line 75) or outcome (e.g., Line 681 and elsewhere). We also learn late in the paper that the experimental protocol aimed to explore the spatial distribution of the stigmata on the boomerang in relation to the knapper's gestures (e.g., Lines 655-657); again, an interesting and worth topic, but this is not developed in the methodology or background discussion. The lack of clarity at the outset of the paper results in a poor structure overall, which in turn leads to questionable decisions about what parts of the study are relevant (e.g., the bone retouching part of the study—this has already been done for European bone indentors; what is new here?) and which data need to be included in the main body of the text. These points are discussed in detail below, by line number. In revision, I suggest adapting some of the relatively more explicit statements of the intent of the study that appear in the conclusion, move them to the beginning, and be far more rigorous about what data is necessary to achieve the intent.

A second issue is that the paper's statement of originality appears to be based on a lack of engagement with the literature. Specifically, 'there is no archaeological, technological, or experimental evidence that wooden and osseous surfaces would have similar reactions to the percussive movement and the consequent impact with lithic edges associated with retouch activities.' Experimental work in using wood retouching tools—for both pressure and percussion flaking—dates back nearly 100 years, if not earlier. For instance, there is an early video online of Coutier using wooden percussion flakers (perhaps written about in his papers in French), and Leakey was experimenting with wood also. An important early example is Crabtree's 1970 manuscript (Crabtree, Don E. 1970 Flaking Stone with Wooden Implements. *Science* 169.3941: 146-153). There are many references to using wood indentors throughout the more recent experimental flintknapping literature, and some modern traditional flintknappers prefer them for some materials and techniques. And much of the published work explicitly compares bone (e.g., antler) and wood for the purpose of

retouching stone tools. In Australia, Moore (2004) anticipated the authors' research by describing boomerang retouching, and discussed the effects of soft-hammer (boomerang) retouching on the morphology of the resulting tula adze slugs. In revision, this literature—minimally, the Crabtree and Moore papers—should be cited, and the statement of originality revised accordingly. This paper is an important contribution, but it doesn't need to be the 'first' to assert this contribution.

Line specific comments: (see attachment)

Line 48. Awkward phrasing. E.g., 'retouching techniques is lacking in the current research interest'. The paper has awkward phrases periodically throughout, and could benefit from a careful language edit. Some of my confusion or misunderstanding may result from issues around expression.

Lines 45-50. 'Retouched lithic tools' needs defined. The claim about a lack of systematic investigation of 'tools shaped by means of percussion retouching techniques' is poorly justified; there are lots of systematic investigations of just this. Indeed, it seems to me that the vast majority of lithic studies in Australia are focused entirely or in part on retouching, either in shaping tool types or gauging reduction intensity by retouching throughout a tool's uselife. The authors have not demonstrated a gap in the literature, as written. It seems to me that part of the issue here is that the study may be adopting a wider European definition of retouching, rather than a more narrow Australian/North American definition.

Line 68: no experimental evidence that wooden and bone would react similarly in percussion flaking. Crabtree 1970 and maybe elsewhere in his work. Coutier, Leakey, Clark, possibly Pelegrin, Bradley, Newcomer. Kamminga?

Lines 74, 76: How is 'function well' as retouchers to be measured in this study? Also, how is 'to easily reproduce' to be measured? Neither are, in fact, measured or evaluated by this study that I could see.

Line 83: The reference to Palaeolithic Europe is a bit of a clanger which points to a Eurocentric bias. Why not Africa? South Asia? America? This also brings up another niggling doubt I have about the usefulness of the paper beyond wooden percussion-flaking tools, which are, to my knowledge, absent from the archaeological record and only known from the Australian ethnographic record world-wide. How can detailed knowledge of the stigmata on wood be bootstrapped to a larger issue (one obvious way is correlating with the 'stimata' on stone flakes and retouched edges, as already done by Moore 2004 for boomerang-retouched adzes)?

Line 87. Methodology is inadequate. How do these articulate with the research question? What exactly is the research question? Why is the manufacturing process for bone retouchers (e.g., Line 116 onwards) important to the research, but manufacture of the boomerangs apparently is not? Also, methodologically-relevant material occurs very late in the text, rather than up front where it belongs. There are vague indications of evaluating various things, but with little information on how (or if) this was done (e.g., see above, Lines 74, 76).

Line 94. The company name is 'Flintknapping Supplies'

Line 102. The authors clam that, in relation to material types, 'variations in stigmata's features are statistically and qualitatively not relevant' (Lines 102-103). I was surprised by this comment, as the authors of the cited reference conclude the precise opposite: 'the marks produced on retouchers differs depending on whether they were used to strike flint or quartzite' (Mallye et al. 2012, quote in the abstract). Also, 'In effect, the scores with smooth surfaces are mainly associated with objects used to retouch flint flakes while those with rough surfaces are associated with objects used to retouch quartzite flakes (chi-square calc . 26.98; $P < 0.001$)' (Mallye et al. 2012:1136). And 'we found that the combination of the morphology of the traces and the types of use areas allows us to deduct the type of raw material retouched' (Mallye et al. 2012:1137). This is a key finding of the Mallye et al. study. Also, material types aside, the authors' study is comparing the marks on wood to bone, so assuming that the experimental results can be transferred from bone to wood at the outset is not very useful and undermines the paper's logic.

Line 151. What is meant by a 'low dash and a crescent number'? Clarify.

Line 160-161. The boomerangs were made 'using traditional methods'. This needs to be expanded and clarified. I doubt very much that they were made using stone tools (in the archaeological terms relevant to this study, this seems to be the criteria for 'traditional' manufacture). To my knowledge, manufacture of boomerangs with stone tools no longer occurs in Australia. If that is correct, then the experiment's boomerangs were made with iron or even power tools (which in my experience is universal for modern boomerang manufacture in eastern Australia)—and the marks on the boomerangs in the photographs certainly look like power tool marks, combined with wood rasp marks. This is an exceptionally important issue, as I have found that boomerang manufacture with stone tools—particularly with tula adzes used in the traditional adzing gesture—can create stigmata just like those documented in this study, including chert microflakes embedded in the adze chop marks. In some ways, boomerangs made by modern tools might be an experimental advantage, as it eliminates this source of variation by creating a smoother, unambiguous surface. Conversely, it makes applying the observations in this paper to archaeological or ethnographic tools more difficult, because the issue of sorting stone tool manufacturing marks from retouching usewear stigmata is not addressed.

Linte 166. Mr Dennis is descended from a coastal SE NSW group (Yuin) and a western NSW group (Wailwaan), but the words suggested for boomerang range from different western NSW groups (Ualaroi, or Gamilaraay/Yuwaalaraay, and Wiradjuri). The 'of this area' in line 167 might be changed to 'of Mr Dennis's people' or similar, as it presently implies that the language names are from SE NSW. The language names for boomerang are not relevant to the study in any case, so this section could be deleted entirely.

Line 181. use 'varies' rather than 'goes'

Line 186. Description of the retouching gesture(s) is necessary here. For instance, Mallye et al. (2012:1133) describe a 'circular percussion trajectory' in their experiments with bone

retouchers. (I return to this several times below). Is this the same gesture used in this study?

Line 191. The knappers must have had some effect they were attempting to achieve in retouching. At what point was their 'arbitrary satisfaction' met? Was it when the edge was completely retouched, for instance? Expand this a bit. (I see that this is covered in the concluding sections of the paper. Move it up front, to the methodology).

Table 3. I began to become confused here, as Table 3 is the result of the experiments but it appears in the methodology section. This is premature, as not all of the methods and definitions reported in the table have been presented. Move Table 3 to the results section. I think tables 1 and 2 can stay where they are, as they are describing the tools used in the experiments, not the results of the experiments per se.

Line 198. Some detail is necessary here on the types of gestures used by YLP to retouch the chert flakes using the boomerang. I see in the Fig 1 caption referent to a 'tangential movement of retouch', but I'm not sure what this means. The figure suggests that the movement was what I would describe as 'flat' with the face of the boomerang slapped onto the edge to be retouched, with minimal follow-through. This is different from the gesture photographed and described in Roth, and Roth's photos in Moore (2004:Fig 2; not cited in the ms, and the only photograph I am aware of showing the use of a boomerang in retouching a stone tool—it should be cited), where the edge or face of the boomerang is swung and an oblique orientation to the tool's edge, thus allowing follow-through. I suggest a new figure, or an addition to Fig 1, that schematically represents the gestures used by YLP in the experiments, and the terms used to describe those gestures. (The video shows YLP using a gesture somewhere between 'slapping' implied by Fig 1 and the very oblique method implied by the historical photograph in Moore 2004:Fig 1. Indeed, the somewhat puzzling technique in Camooweal of hafting the tula at right angles to the handle, specifically for boomerang retouching, may in fact have been done to allow this rather oblique gesture, thus allowing more follow-through than allowed by YLP's gesture).

Lines 224-225. The authors state that the categories of usewear documented are 'valid for both bone retouchers and boomerangs used as retouchers', but isn't this what the experiments were meant to assess? There are some problems with the epistemology here. If the marks are known to be the same *a priori*, then what was the point of the experiments? This is a fundamental issue with the paper, here and elsewhere.

Lines 225-229. I suggest a new composite figure here as a guide, with an example of each mark labelled. The definitions are vague, using descriptors such as 'deep' and 'wide', so some photographs would help here.

Lines 230-238. More clarity is needed here. How was the 3D modelling done? Was it generated by the microscope by image stacking, or what? What was the instrument used? What scales were used? What is the margin of error for measurements? What are the 'equal distances' referred to?

Lines 240-241. Describe these categories of intensity, and how they are recognised/defined.

Line 257. I am unsure why bone retouching was necessary for this study, as the stigmata on bone retouchers have been extensively described and analysed in prior archaeological and experimental studies. It does not appear that anything new emerged from this exercise for the bone tools, particularly since the results were analysed using the conventions reported previously in these various studies. Table 4 seems superfluous to the aim of the study.

Line 268. How were blows counted? This is not described in the methodology. Was this determined by watching the videos, or by some other method?

Table 4. The various terms in the footnote of the table need to be described, as it is not immediately obvious what they refer to. However, many or most appear to be not particularly relevant to the aim of the manuscript, which is about stigmata on wood indentors from retouching. The paper is not about the stones per se, and indeed, they do not figure in any substantive way in the discussions or conclusions. The table could be made much simpler, leaving out most of this data.

Lines 289-290. Again we see something about the intentions of a knapper (see also comment for line 191). In this case, the intent was to remove long, thin flakes, which seems different from seeking to produce a certain type of edge. That is, the focus is on detaching certain types of flakes rather than creating an edge morphology. This seems more akin to core reduction. Some clarification/discussion is necessary, probably in the methodology section.

Line 292. What is meant by a 'stepped in morphology'? Does this mean the flake scars ended in step terminations? This doesn't fit comfortably with the description of the flakes as long and thin, combined with the retouched edges described as 'semi-abrupt' and 'abrupt'. I also don't know what is meant by 'the retouch is located in a direct position'. More clarity is necessary.

Line 296. Here we read that the bone retouching gesture is 'tangential', the same as with the boomerang. Given the great difference in morphology of these indentors types, is it appropriate to use the identical term for the gesture(s) used in employing them? More clarity in description of the gestures, and variants of the gestures, is necessary.

Line 297. What are 'soft taps', and how do these relate to the tangential 'retouching movement'?

Line 321. 'Struck', not 'stroke'

Line 323. I don't understand 'the radius of the arch results in 7.4 mm on average'. Is this a thickness? By convention, it is good practice to list the standard deviation and sample size (i.e., 13 +/- __ mm, N=__).

Line 324-325. Here is a bit more information on the gestures used in the bone retouching and the boomerang retouching, but it is still unclear to me. How are the two related exactly? How are they different/similar?

Line 336. What is meant by 'scaled on the left and right edges'? Define the terminology used, or at least identify the source for a definition, with page number. What is the convention used to identify left and right edges (e.g., proximal end up, dorsal surface up, or proximal end down, dorsal surface up, or some other convention)? This comment applies across much of the description of results. Alternatively, much of this information is not strictly germane to the research question, so might be deleted.

Line 341. What does 'cross-section in correspondence of stigmata' mean? I think this could be simplified. This is not the normal convention for showing a cross section. The profile line across the object should be shown with tick marks and keyed to the cross section drawing.

Line 358. Here is another reference to the knapping gesture, this time referring to a parallel trajectory and contrasting it with a tangential trajectory. If the movement is parallel to the edge, how is a flake detached? Again, I think a figure is necessary to untease these variations, rather than describing them in words.

Line 381. 'the retouch is considered completed'. This suggests an intention on the part of the flintknapper, which should be described in the methodology.

Line 389. Again, this refers to an unspecified goal on the part of the flintknapper. This needs to be discussed in the methodology.

Line 466 and thereafter. Rephrase to remove the word 'goes'. Perhaps 'varies' is a better word to use here. Rote data descriptions like this are best put into summary tables rather than described in text.

Lines 475-476. The authors assert empirical differences between boomerangs and bone retouchers. This must be accompanied by statistical tests. If there appears intuitively to be a difference, then test this with statistics. All the data is there I think, so it shouldn't be an onerous task.

Lines 480-482. The authors discuss partial differences in lengths and widths (not sure what that means), but greater differences in surfaces and perimeters, but surely the perimeters are a function of the lengths and widths? This whole discussion is important and should be expanded and documented using statistical tests. (This comment applies for most of the analysis which follows—statistical tests are appropriate to support the various assertions).

Line 485. I don't think the categories are defined anywhere. I do not understand how you can have concentrated and dispersed distributions superposed without it resulting in a concentrated distribution.

Line 493. The authors suggest that the retouching intensity on the retouchers varied according to the flintknapper's techniques, but the cited data (S9 Fig 2c) don't support this conclusion because the data is not comparable. It is possibly that YLP's technique varied from TRM's because YLP used both types of retouchers, and TRM used only bone retouchers. The variation is very likely related to the radically different nature of these

types of retouchers, and the adjustments to gesture necessary to make them work properly. That is, they might have used identical gestures, but TRM didn't have to adjust his because they didn't take part in the boomerang experiment. I also note that YLP was naïve to using boomerang retouchers prior to the experiment, which is a complicating factor when attempting to characterise YLP's technique.

Line 562. Is it possible that some of these marks (e.g., a-d) were created during the manufacture of the boomerangs using wood rasps and mechanical/abrasive power tools?

Line 576. I note that Mallye et al. (2012: Fig 2) identified 'scaled areas' among pits and scores in their bone retoucher experiments that seem analogous to the 'small detachments' identified by the authors on the boomerang retouchers. This might be a more likely correlation than the peeling marks cited, and it should be mentioned and discussed. See also lines 737-738.

Lines 579-583. This is not necessary here, as a whole section on this appears from Line 600.

Line 585. Again, lots of assertions based on data, but no statistical tests to back up these intuitive assessments. Some statistical tests are warranted here.

Line 586. The authors should move more of this important data out of the supplementary information and into the main text. This whole section, for instance, is carried by data in the supplementary information. Supplementary information should be for supplementary data or raw data, not for the data which is key to the argument.

Line 625-627. I strongly dispute this assertion. Ethnographic and anthropological accounts of boomerangs in Australia frequently describe how boomerangs are used for many, many things, both functionally and ritually. I would go so far as to suggest that ethnographic accounts of boomerangs in traditional societies almost always described (or implied) how they functioned in various roles, not just for throwing at animals. Archaeologists have also been explicit about the use of boomerangs for stone tool manufacture (e.g., Moore 2004, and possibly some of the earlier 20th century research on tool types). It would appear that this paragraph is a way of positioning or promoting the authors' research, which is not inherently a bad thing, but it should be done thoughtfully and self-reflexively, and not by ignoring decades of prior research.

Lines 636-639. This succinct summary of the method is just appearing here for the first time. I am a little confused at how the descriptions of the retouched edges related to 'functionality', because the latter term is not defined. Boomerangs can be used to retouch, but we know this from ethnographic reports spanning over 100 years. So what is this study attempting to achieve exactly? I also note that the flintknapper's feedback is not presented systematically, and there is nothing in the methodological description about how this feedback was collected. And traceological similarities between bone and wood doesn't inform us about functionality per se, depending on how that is defined.

Line 641. Here we have something more explicit on the intent of the flintknappers. This should appear in the methodology, not the conclusions. How did the flintknappers

differentiate between the retouched Australian tools, and the Discoid/Quina Mousterian tools? Was there a perception of different functional criteria for the cutting/scraping tools in these two industries? This is important for discerning when the flintknapper decided a tool was 'finished', which in turn relates directly to the nature and extent of the stigmata distribution on the boomerang retouchers in particular.

Line 655-657. Something new appears here re the experimental protocol, about the spatial distribution of wear and the knapper's gesture(s). This should have been in the methodology.

Line 675. The traceological section is the strongest part of the paper, and has the most to contribute. I suggest that with this section in mind, the authors should revise the introduction and methodology to lead more seamlessly to this discussion. Some of the weaker parts of the manuscript can be deleted, with a clear and single-minded focus on the conclusions that appear in this section.

Line 681. The authors say that their data matches prior research on bone retouchers, but this is not surprising as they already concluded this at the outset of the study (Lines 224-225).

Line 707. Typo

Lines 707-709. This is good, but how applicable is it to Australia, where archaeological boomerangs are exceptionally rare and (probably) too poorly preserved to retain evidence of retouching? Identifying boomerang retouchers is most relevant to ethnographic collections in museums, unless their use can be related more specifically to the stone artefacts, as Moore (2004) has done in identifying soft-hammer retouched tulas.

Line 764. The authors claimed to have 'minimised the variables' but this isn't discussed explicitly in the methodology. What variables were minimised, and how was this accomplished?
